# Synthesis and Biological Evaluation of 1,3,5-Triazine Derivatives for *GPR119* Agonistic Activity

**DOI:** 10.3390/molecules31183289

**Published:** 2026-09-16

**Authors:** Bui Hoang Huu Nhan, Seong Ho Jeon, Sujin Lee, Seung Hwan Son, Young Duk Yang, Seok-Ho Kim

**Affiliations:** 1 College of Pharmacy, Kangwon National University, Chuncheon 24341, Republic of Korea; 202116517@kangwon.ac.kr (B.H.H.N.); shson@kangwon.ac.kr (S.H.S.); 2 Department of Pharmacy, College of Pharmacy and Institute of Pharmaceutical Sciences, CHA University, Pocheon 11160, Republic of Korea; cmb_jsh@naver.com (S.H.J.); 3 College of Pharmacy, Daegu Catholic University, Gyungsan 38430, Republic of Korea; sujinlee20@cu.ac.kr (S.L.)

**Keywords:** diabetes, *GPR119* agonist, 1,3,5-triazine, GLP-1, molecular docking

## Abstract

*GPR119* is a promising target for the treatment of type 2 diabetes mellitus. Stimulating this receptor leads to the induction of glucose-dependent insulinotropic peptide and glucagon-like peptide 1, which improves systemic glucose homeostasis. Encouraged by our previous research, we replaced 5-nitropyrimidine with 1,3,5-triazine to reduce hepatotoxicity. A new series of compounds with a 1,3,5-triazine scaffold was synthesized and evaluated for their agonistic activities against human *GPR119*. Thirty-three compounds were synthesized and evaluated for their potential *GPR119* agonist activities. Analogs with a 4-cyano and a 4-methylsulfonyl group in the aryl ring exhibited more potent activation than those analogs with a 4-trifluoromethyl group. Compound **3c** had the most favorable EC_50_ value (hEC_50_ = 2.39 µM) and significantly induced glucagon-like peptide-1 secretion. Furthermore, docking simulations showed that compound 3c stably binds to the active site of *GPR119*.

## 1. Introduction

Diabetes, the seventh leading cause of fatality worldwide, is caused by either the inability of the pancreas to produce sufficient insulin or the inability of body cells to respond properly to insulin [1]. Diabetes is categorized into three types: type 1 diabetes mellitus results from the immune system destroying the pancreatic β-cells; type 2 diabetes results from the body cells losing their sensitivity to insulin, sometimes even causing absolute insulin deficiency; and gestational diabetes, the third main form, occurs during pregnancy without a previous diagnosis of diabetes [2,3]. Type 2 diabetes mellitus (T2DM), more specifically, has been a heavy burden for every patient because the disease is typically associated with a 10-year reduction in life expectancy and other complications such as cardiovascular disease, infection, cognitive impairment, and dementia [4,5,6]. Therefore, the development of new options for anti-diabetic drugs has been a top priority for medicinal chemists. Currently, multiple clinical therapies for T2DM have been developed, including insulin-targeting routes such as biguanides [7,8], thiazolidinediones [9,10], sulfonylurea derivatives [11], incretin-modulating agents such as glucagon-like peptide-1 (GLP-1) analogs [12,13], and dipeptidyl peptidase 4 inhibitors [14,15], as well as increasing glucose excretion through sodium–glucose cotransporter 2 inhibitors [16].

*GPR119*, which is mostly expressed on enteroendocrine cells (L- and K-cells) in the intestine and on β-cells within the islets of Langerhans in the pancreas, has gained attention as a new target for T2DM treatment [17]. Ligation of *GPR119* by an agonist leads to the activation of adenylate cyclase and a rise in cyclic adenosine monophosphate (cAMP) levels, thus triggering the secretion of incretin hormones such as glucose-dependent insulinotropic peptide (GIP), GLP-1, or insulin from K-cells, L-cells, and β-cells. Additionally, GIP and GLP-1 can interact with their corresponding receptors on the pancreatic β-cell to elicit insulin production. As a result, *GPR119* agonists enhance insulin release during food intake through indirect and direct mechanisms. Because GLP-1 (and presumably GIP) also promotes the viability of β-cells, it is possible that *GPR119* agonists can influence the secretory activity and preservation of β-cells, resulting in improved glucose homeostasis in T2DM patients [18].

**Figure 1:**
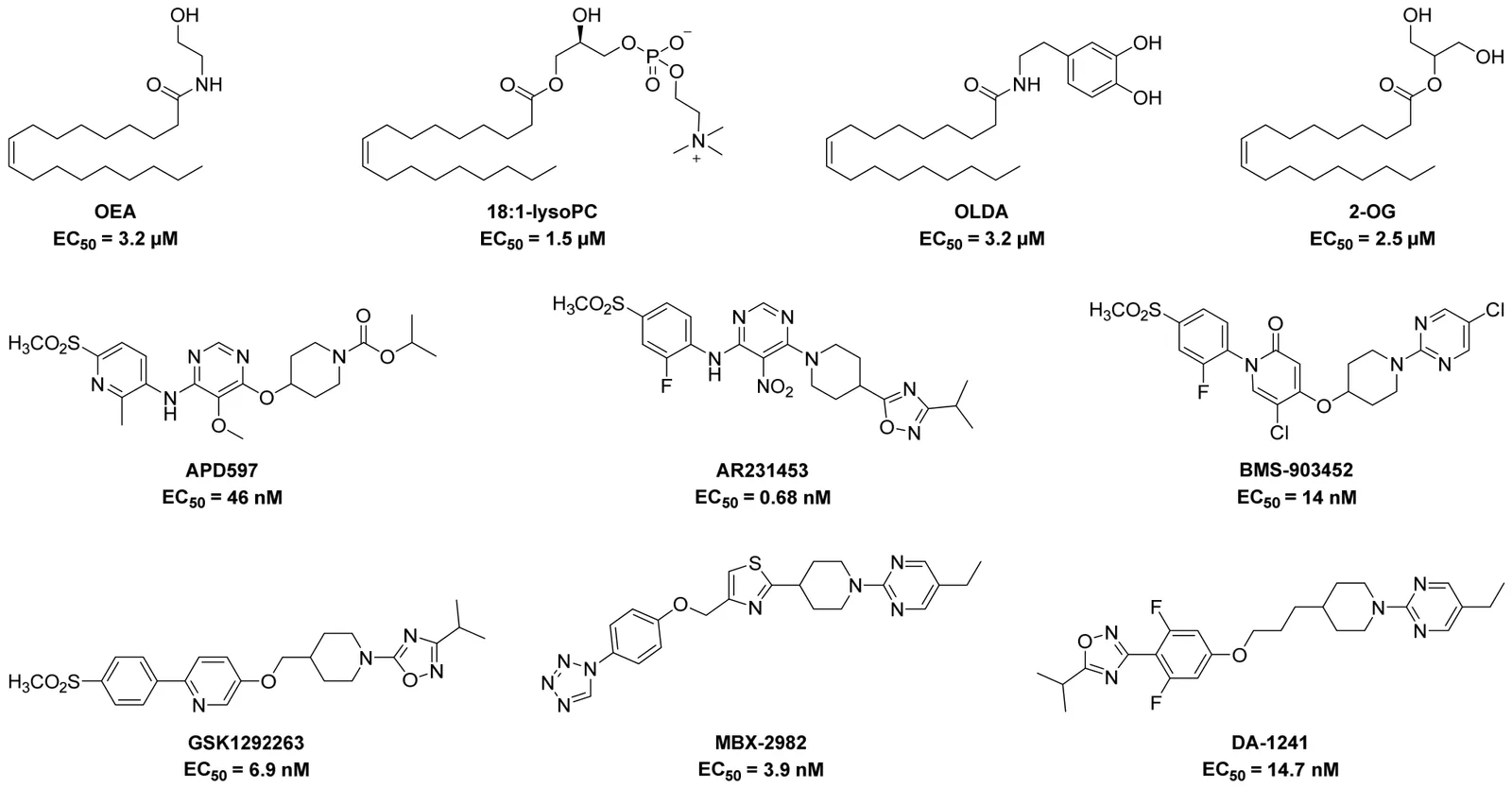
Structures of known *GPR119* agonists.

Several endogenous lipid metabolites exhibit agonistic effects on *GPR119*, including oleoylethanolamide (OEA) [19,20], 1-oleoyl-lysophosphatidylcholine [21], *N*-oleoyl-dopamine [22], and 2-oleoylglycerol [18,23]. Other studies have also confirmed that *GPR119* agonists can improve glucose tolerance and preserve β cell function in rodents and humans. The first potent and orally efficacious small molecule agonist of *GPR119*, AR231453, was discovered by Semple’s group [24]. Compound AR231453 exhibited potent agonistic activity (EC_50_ = 0.68 nM) and improved oral glucose tolerance in wild-type mice, but not in *GPR119*-deficient mice. Another breakthrough was DA-1241, a compound first reported in 2019 with potent agonistic activity against human *GPR119*. The compound exhibited an increase in cAMP levels in HIT-T15 insulinoma cells (EC_50_ = 14.7 nM) and an increase in insulin secretion (EC_50_ = 22.3 nM). DA-1241, under the generic name “Vanoglipel”, is currently under clinical trials [25,26,27]. Following these discoveries, several pharmaceutical companies and academic institutes have been pursuing *GPR119* agonists as potential drug candidates for T2DM treatment. As a result, several *GPR119* agonists have been developed as clinical treatments for T2DM, as shown in Figure 1 [28,29,30,31,32,33].

**Figure 2:**
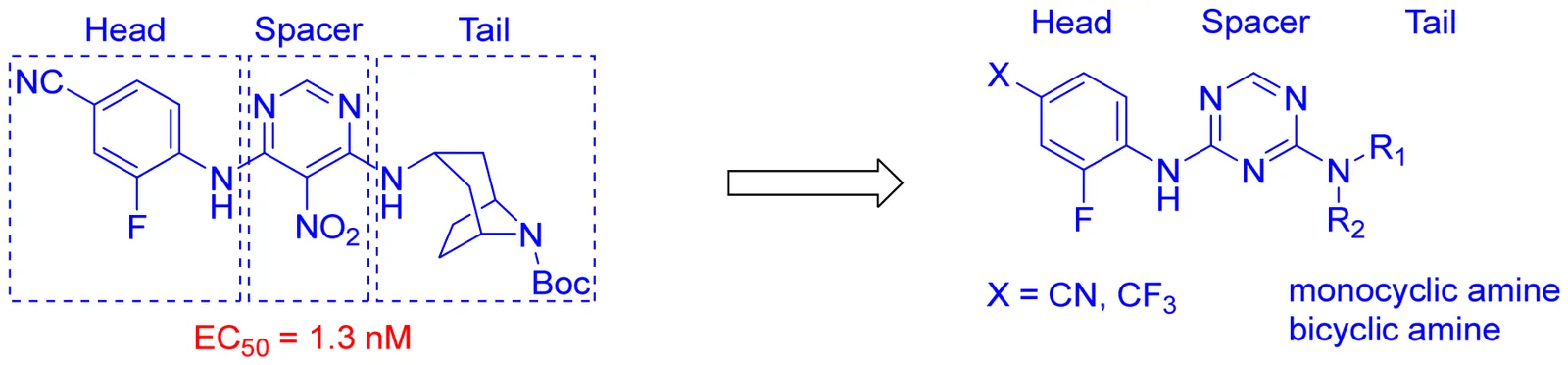
Representative *GPR119* agonist and three key pharmacophores.

Based on the reported structure–activity relationship (SAR) analysis data, most *GPR119* agonists consist of three crucial pharmacophores. The “head” is an aryl or heteroaryl ring substituted with electron-withdrawing groups (methylsulfonyl, nitrile, tetrazolyl), and the “tail” contains a piperidine or piperazine *N*-capped with a hydrogen-bond acceptor group (carbamates, substituted oxadiazoles or a 5-substituted pyrimidine ring) (Figure 2). Connecting these two pharmacophores, the “spacer” usually contains an aryl or heteroaryl structure that can ensure optimal distance and orientation [34,35].

In our efforts to discover a novel small molecule agonist of *GPR119*, we built on the results of our previous study, choosing 4,6-disubstituted 5-nitropyrimidine with 4-amino-3-fluorobenzonitrile and a conformationally restricted 4-aminopiperidine ring as the lead structure [35]. As disclosed in our previous papers, SAR analysis studies have indicated that: (1) the 5-nitropyrimidine scaffold possesses strong agonistic activity against *GPR119*; (2) the endo-azabicyclic conformation could be considered the “agonist conformation”; (3) a fluorine atom at the ortho position of the aniline moiety greatly improves agonistic activity against *GPR119*; (4) electron-withdrawing groups (EWGs) such as methylsulfonyl and the nitrile group at the para position of the aniline moiety can enhance agonistic activity against *GPR119* [35,36]. In our attempts to develop highly potent *GPR119* agonists, hepatotoxicity has always been observed in compounds with a nitro group [37,38]. In addition, we observed that the fused 2,4-disubstituted 1,3,5-triazine core could be a bioisostere for the 5-nitropyrimidine due to their similar structures and polarities [39]. The 1,3,5-triazine can be easily synthesized from commercially available cyanuric chloride **1**. Therefore, we chose 1,3,5-triazine as the core structure. Then, various anilines substituted with EWGs were provided as the head moiety linked to the novel scaffold. Finally, we report the synthesis of a series of 1,3,5-triazine derivatives containing monocyclic piperidine and monocyclic or bicyclic piperazine moieties, including endo/exo structures, as potential *GPR119* agonists for T2DM treatment [40,41].

## 2. Results and Discussion

### 2.1. Chemistry

**Scheme 1:**
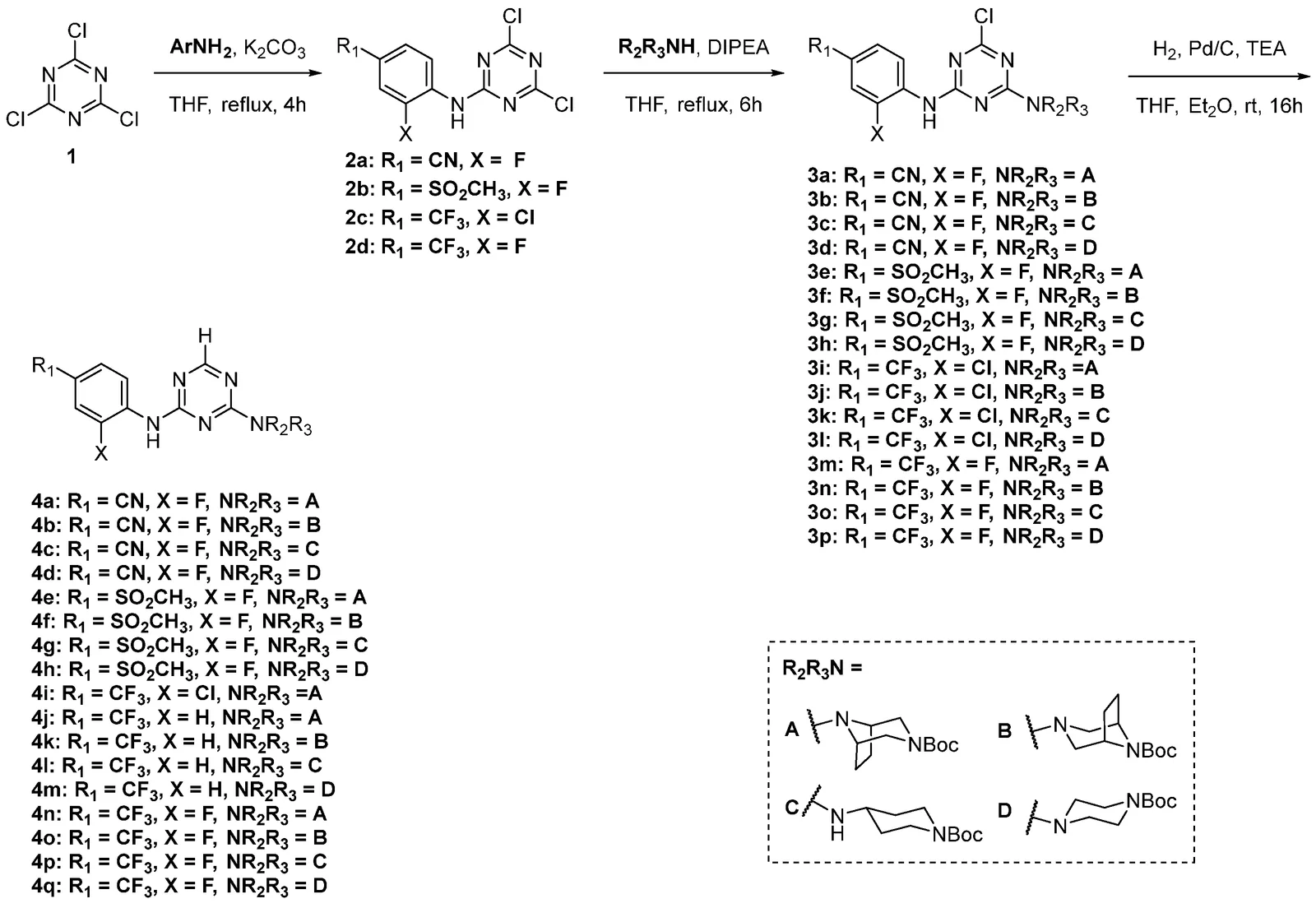
Synthetic route of compounds with 1,3,5-triazine core.

The synthetic scheme of the targeted compounds is depicted in Scheme 1. Commercially available cyanuric chloride **1** was treated with aniline derivatives to obtain the mono-substituted intermediates **2a**–**2d**. **3a**–**3p** were obtained by treatment with piperidine (**C**) or piperazine derivatives (**A**, **B**, **D**) (see Table 1). **4a**–**4q** were also successfully synthesized from the dehalogenation of the corresponding chlorine analogs (**3a**–**3p**) under a hydrogen atmosphere in the presence of Pd/C and triethylamine (see Section 3). During this step, the chlorine atom of the 2-chloro-4-trifluoromethyl aniline moiety of **3i**–**3l** was also removed under the same conditions, which led to the formation of compounds **4j**–**4m**. **4i** was also isolated from the reaction of **3i** together with **4j** (see Experimental Section 3.9). Detailed synthetic procedures are described in the Experimental Section.

### 2.2. In Vitro Assay

**Figure 3:**
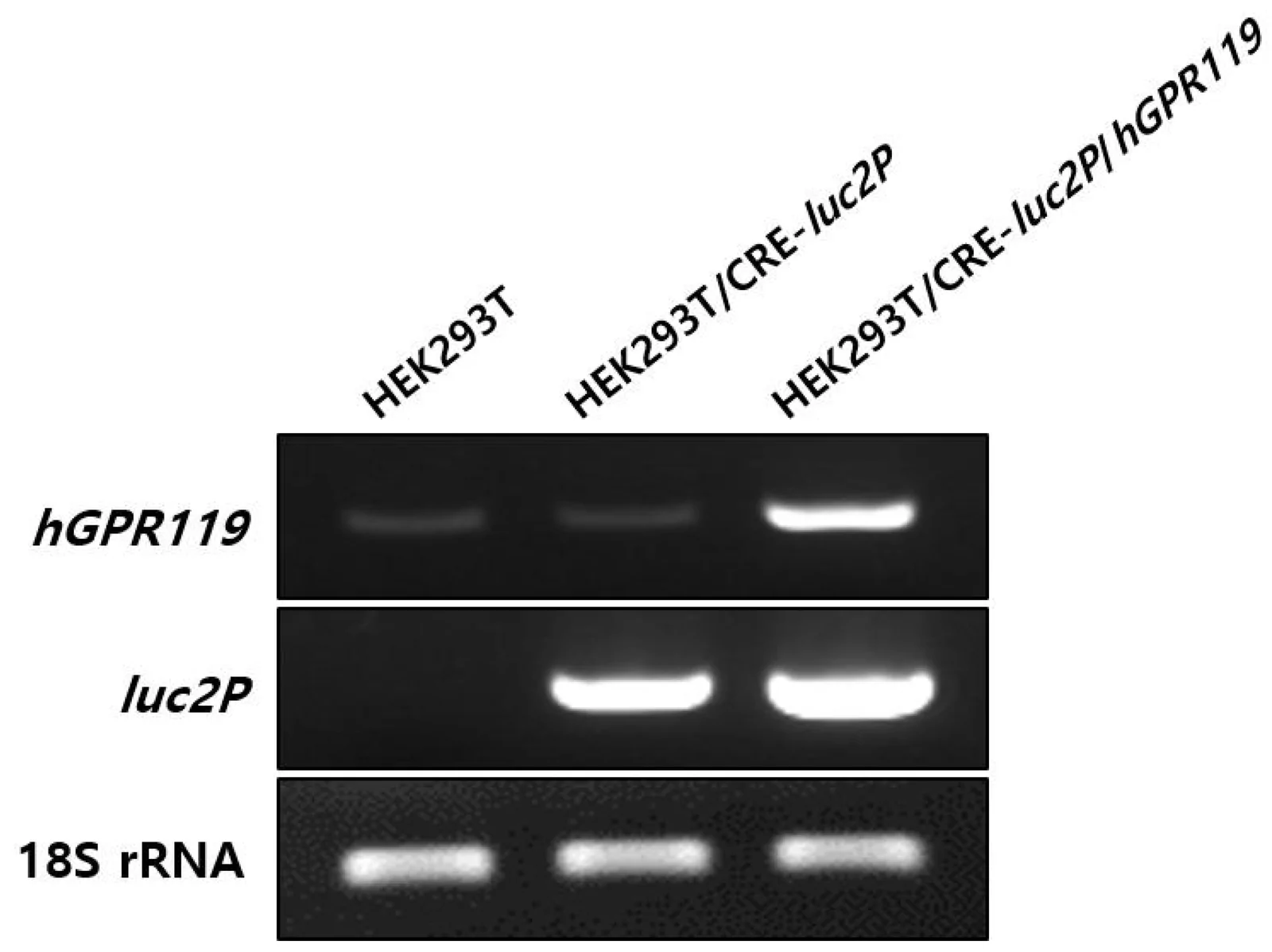
Establishment of stable cell lines for *GPR119* agonist screening. A dual stable cell line was established from parental HEK293T cells through stepwise random integration into the host cell genome. First, the HEK293T/CRE-*luc2P* stable cell line was established using the pGL4.29 vector containing a cyclic adenosine monophosphate response element (CRE) driving the expression of the luciferase reporter gene *luc2P*. Subsequently, the HEK293T/CRE-*luc2P*/*hGPR119* dual stable cell line was generated by transfection of the pCMV6-*hGPR119*-Myc-DDK Tag vector encoding human *GPR119*. The mRNA expression of *hGPR119* and *luc2P* in the established stable cell lines was confirmed by reverse transcription polymerase chain reaction.

To establish a cell-based screening system for identifying novel *GPR119* agonists, we constructed a dual-expression stable HEK293T cell line that simultaneously encodes the human-derived *GPR119* gene and the *luc2P* gene, transcribed through the interaction of the cAMP response element (CRE) and the cAMP response element-binding protein (CREB) (see Figure 3). The *Photinus pyralis*-derived *luc2P* gene is a human codon-optimized luciferase reporter designed for high expression and reduced aberrant transcription. It contains an hPEST destabilization sequence that enables rapid signal turnover. Increased intracellular cAMP activates CREB, and activated CREB subsequently binds to CREs, thereby inducing CRE-dependent *luc2P* transcription [42]. Therefore, this screening system can be applied such that *GPR119* accumulates cAMP in response to agonists, and the CRE/CREB signaling mediated by cAMP accumulation subsequently promotes *luc2P* transcription.

We screened thirty-three compounds using the established stable reporter cell lines to identify novel agonists for human-derived *GPR119*. Here, OEA and AS1269574, known *GPR119* agonists, were used as reference controls for *GPR119* activation. Additionally, Forskolin, which accumulates cAMP independently of *GPR119* activation by directly activating adenylyl cyclase and consequently activates cAMP-dependent protein kinase, was used as a positive control to verify whether this screening system appropriately responds to intracellular cAMP levels.

In the primary screening, the HEK293T/CRE-*luc2P* and HEK293T/CRE-*luc2P*/*hGPR119* stable cell lines were seeded to approximately 80–90% confluence, and then cells were treated with each compound at a concentration of 10 μM. After 6 h of treatment, the results were validated by measuring luciferase activity.

**Figure 4:**
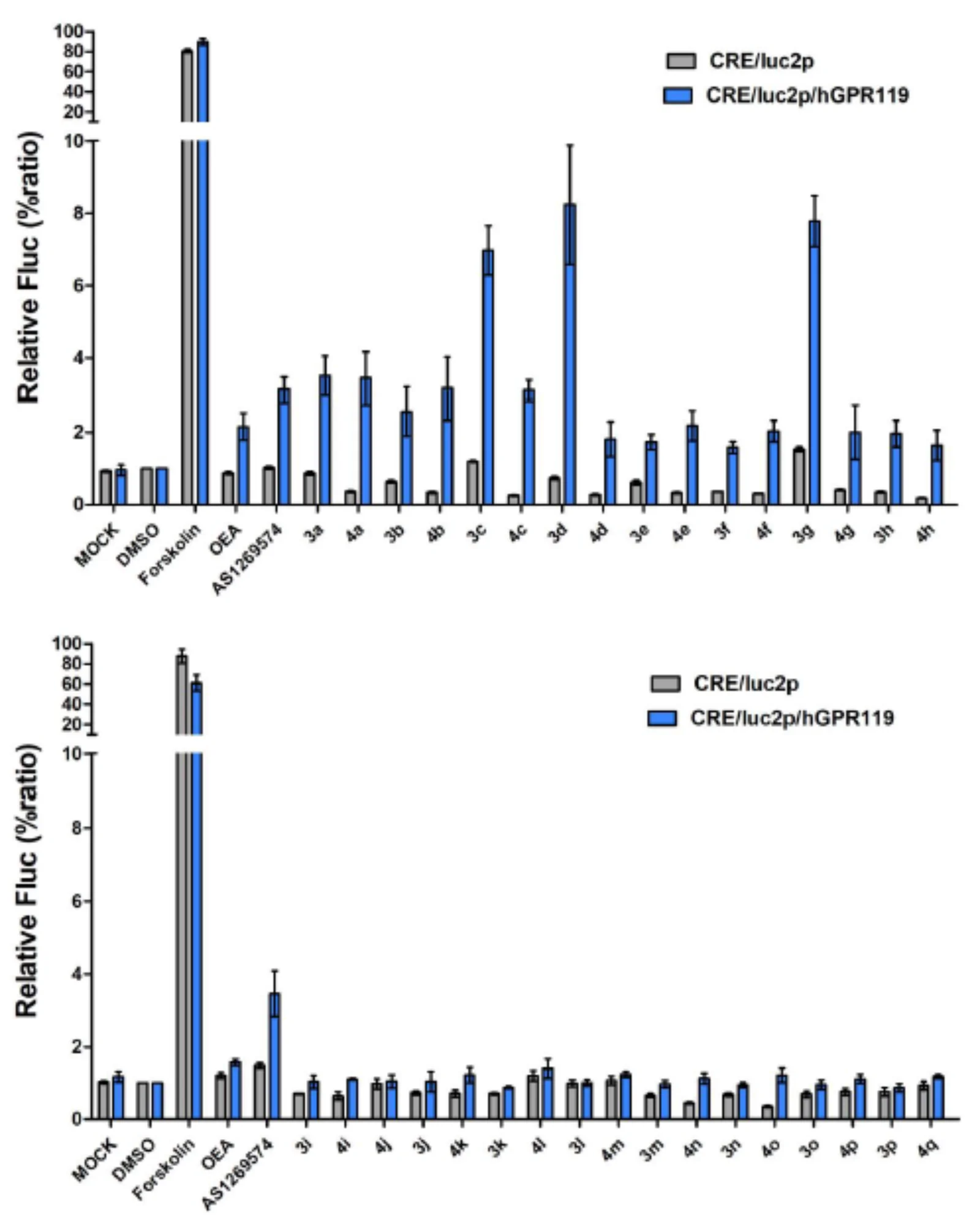
Primary screening of compounds for *GPR119* agonistic activity. HEK293T/CRE-*luc2P* and HEK293T/CRE-*luc2P*/*hGPR119* stable cell lines were treated with 33 compounds at 10 μM for 6 h, followed by measurement of luciferase activity. Forskolin was used as a positive control for the cyclic adenosine monophosphate (cAMP)-responsive reporter system, whereas oleoylethanolamide (OEA) and AS1269574 were used as reference controls for *GPR119* activation. CRE: cAMP response element.

As expected, the high luciferase activity level observed upon Forskolin treatment demonstrated that this screening system responds sensitively to increased intracellular cAMP levels. This result suggests that it is an appropriate method for screening intracellular cAMP levels (see Figure 4). Moreover, when the cells were treated with AS1269574 and OEA, which were used as reference controls for *GPR119* agonistic activity, the luciferase activity level was significantly increased in the CRE-*luc2P*/*hGPR119* stable cell line relative to that in the CRE-*luc2P* stable cell line. This indicates that the dual-expression stable cell line exhibits superior responsiveness to *GPR119* agonists (see Figure 4).

In the screening of thirty-three compounds, compounds **3a**–**3h** and **4a**–**4h**, which bear a cyano or mesyl substituent at the *para* position of the aniline moiety, generally exhibited agonistic effects against *GPR119*, whereas compounds **3i–3p** and **4i–4q**, which have a trifluoromethyl group, exhibited no significant differences in luciferase activity compared with the Mock and DMSO treatments in the control cells. Based on their relative luciferase responses in HEK293T/CRE-*luc2P*/*hGPR119* cells (expressed as fold changes relative to the DMSO control, mean ± SEM from three independent experiments), the compounds were categorized into four groups: strong, active, moderate, or inactive (Table 2). In particular, compounds **3c**, **3d**, and **3g** demonstrated stronger agonistic effects on *GPR119* than the known agonists OEA and AS1269574. This high potency may be attributed to the flexible monocyclic amine groups (R_2_) facilitating optimal accommodation within the binding pocket, while rigid bicyclic groups led to activity loss. Furthermore, the luciferase activity level specifically increased in the dual-expression stable cell line overexpressing *hGPR119* compared with that in the CRE-*luc2P*-single stable cell line. These results suggest that compounds **3c**, **3d**, and **3g** exhibit selectivity for agonistic activity against *GPR119* and demonstrate greater efficacy than the known agonists. Thus, we initially selected compounds **3c**, **3d**, and **3g** as potential novel agonist candidates against *GPR119* and conducted follow-up research (see Figure 4 and Table 2).

**Figure 5:**
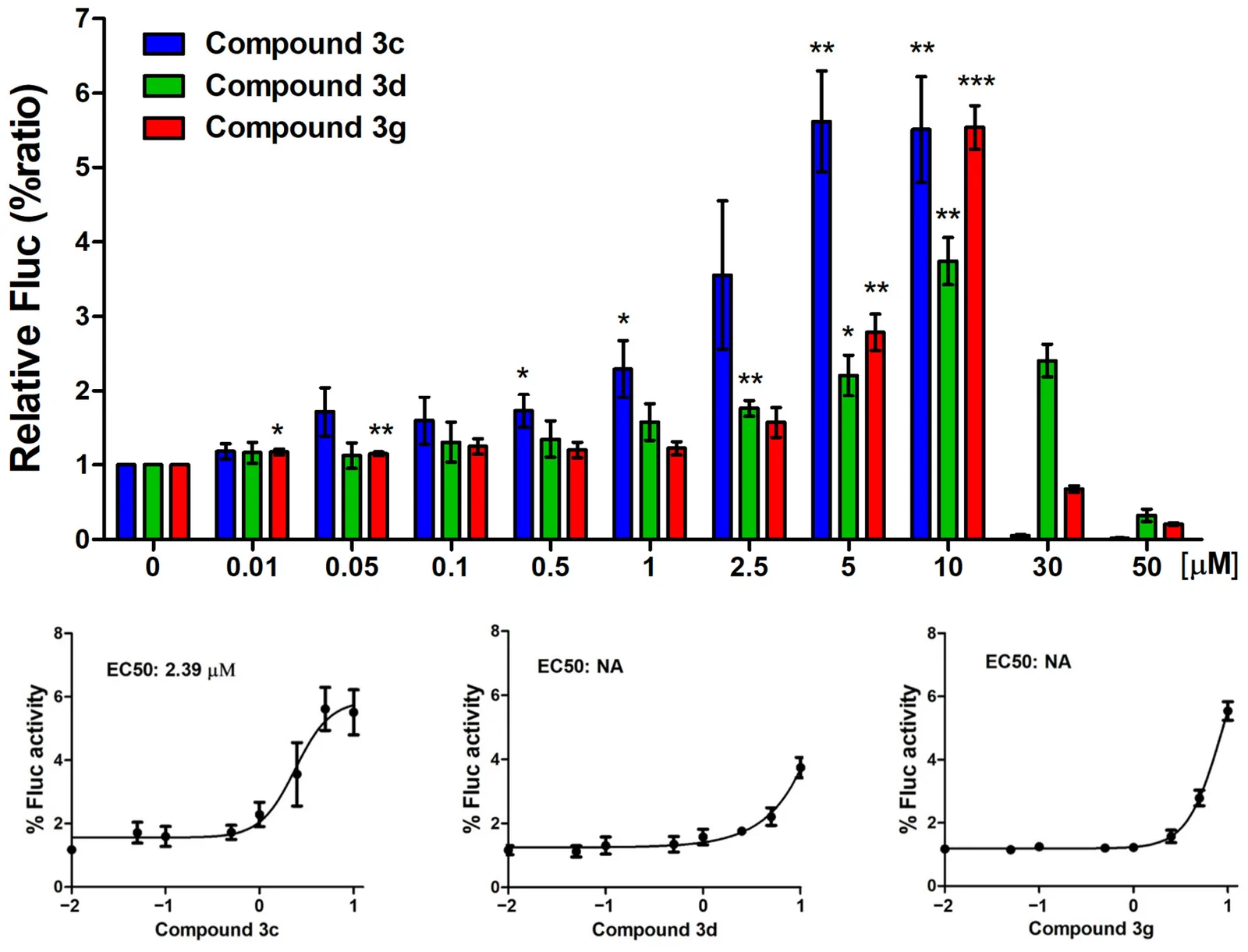
Determination of EC_50_ values of selected compounds for *GPR119*-mediated luciferase activity. HEK293T/CRE-*luc2P*/*hGPR119* stable cells were treated with compounds **3c**, **3d**, and **3g** in a concentration-dependent manner for 6 h, followed by measurement of luciferase activity. Concentration–response curves were generated to determine the EC_50_ values. Compound **3c** exhibited an EC_50_ value of 2.39 μM, whereas the EC_50_ values for compounds **3d** and **3g** could not be determined. Data are presented as the mean ± SEM from three independent experiments. * *p* < 0.05, ** *p* < 0.01, and *** *p* < 0.001 compared with the DMSO control. CRE: cyclic adenosine monophosphate response element.

We evaluated the concentration-dependent agonistic efficacy of the three selected compounds (**3c**, **3d**, and **3g**) on *GPR119* using a dual-expression stable cell line. The data were normalized to those of untreated control cells, and the compounds were tested at concentrations ranging from 0.01 to 50 µM. After treating the cells with the compounds for 6 h, compound **3c** demonstrated the highest efficacy within the concentration range of 5 to 10 µM, and the EC_50_ value was determined to be 2.39 µM. Meanwhile, compounds **3d** and **3g** exhibited the highest efficacy at a concentration of 10 µM; however, their EC_50_ values could not be determined, possibly due to the observed cytotoxicity at higher concentrations (see Figure S1). Therefore, further structural optimization aimed at reducing cytotoxicity will be required to develop these compounds as novel *GPR119* agonists (see Figure 5).

**Figure 6:**
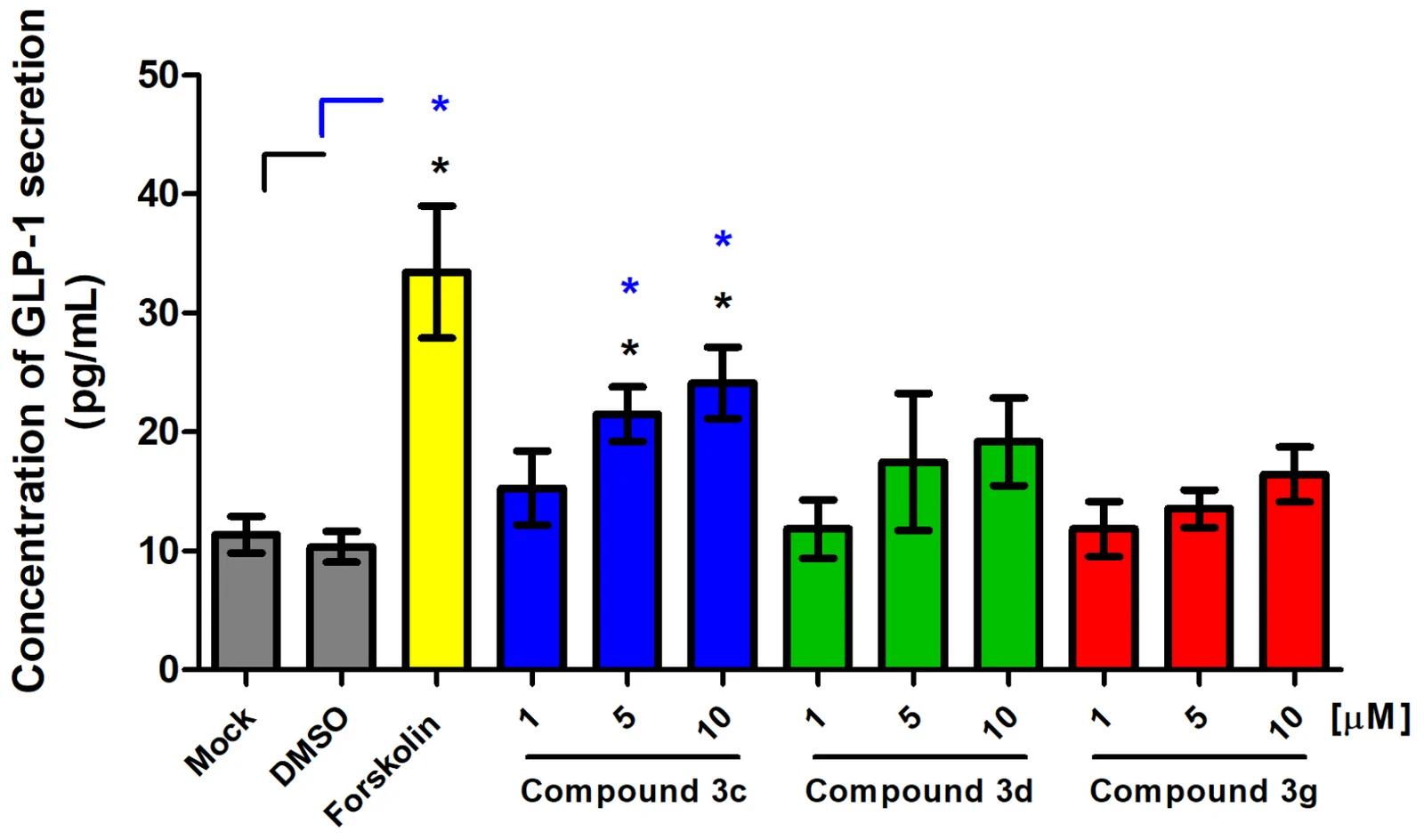
Effects of selected compounds on GLP-1 secretion in NCI-H716 cells. NCI-H716 cells were treated with compounds **3c**, **3d**, and **3g** at concentrations of 1, 5, and 10 μM for 2 h, and glucagon-like peptide-1 (GLP-1) concentrations in the culture supernatants were measured using a human GLP-1 enzyme-linked immunosorbent assay kit. Forskolin was used as a positive control. Data are presented as the mean ± standard error of the mean from three independent experiments (*n* = 3). Statistical significance was determined using a two-tailed paired *t*-test. * *p* < 0.05 versus the Mock group (black asterisks) or the DMSO group (blue asterisks).

To evaluate how the indicated compounds affect GLP-1 secretion, differentiated NCI-H716 cells were treated with each compound at concentrations of 1, 5, and 10 μM for 2 h, after which GLP-1 concentrations in the culture supernatants were measured by enzyme-linked immunosorbent assay. Forskolin, used as a positive control, significantly enhanced GLP-1 secretion compared with the untreated (Mock) or DMSO (NC) controls. Among the tested compounds, compound **3c** significantly upregulated GLP-1 secretion at 5 and 10 μM, whereas a modest increase was observed at 1 μM. In contrast, compounds **3d** and **3g** enhanced GLP-1 secretion in a concentration-dependent manner, although the effects were not statistically significant. These results suggest that compound **3c** significantly promotes GLP-1 secretion in NCI-H716 cells at concentrations ranging from 5 to 10 μM (see Figure 6).

**Figure 7:**
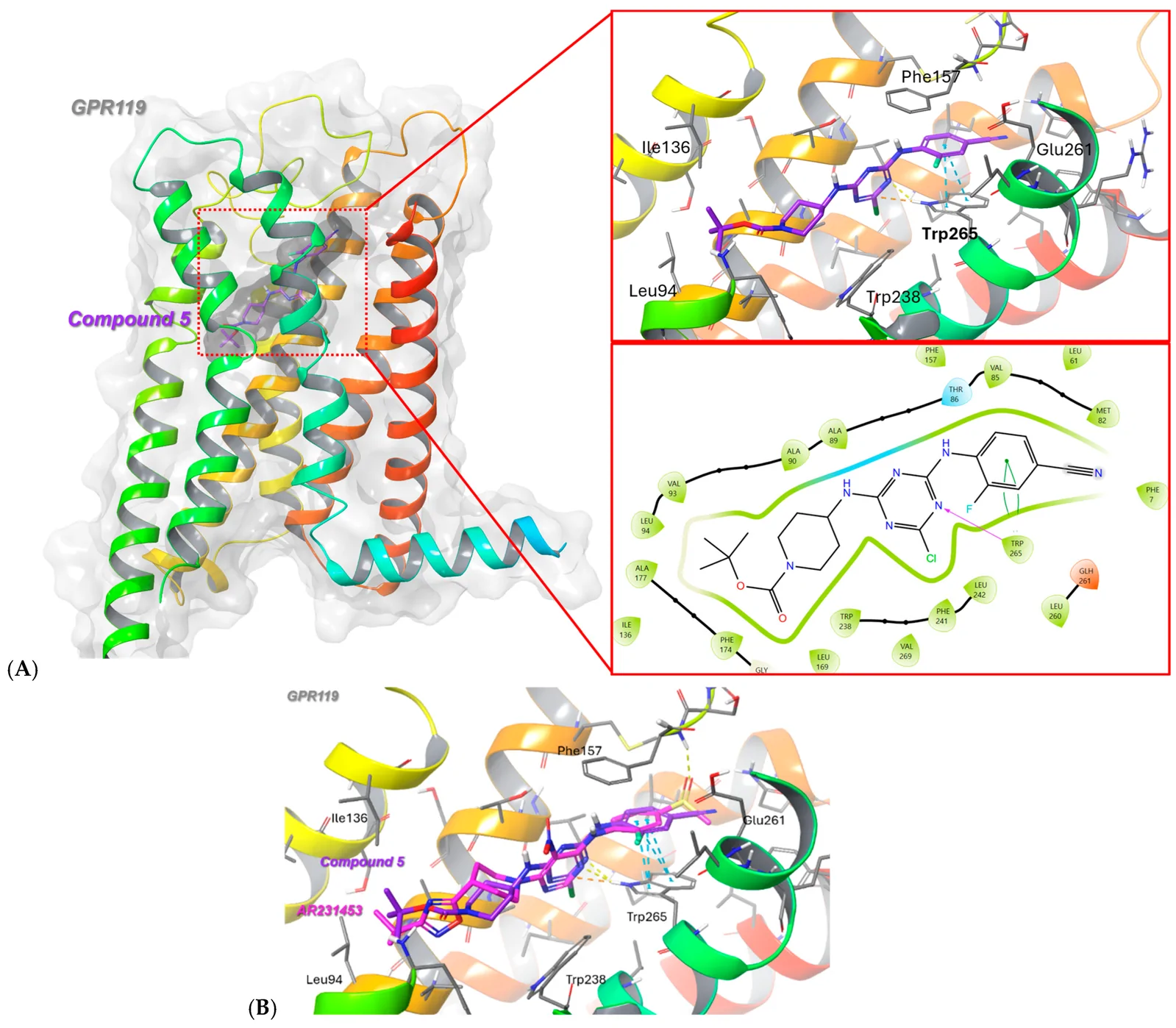
(**A**) Predicted binding mode of compound **3c** within the *GPR119* binding pocket. The *GPR119* structure and compound **3c** are colored cyan and purple, respectively. Key interacting residues in *GPR119* are depicted as sticks, with their respective residue numbers indicated. (**B**) Superimposition of the docking poses of compound **3c** (purple) and AR231453 (magenta) in the *GPR119* binding pocket.

To predict the binding affinity of compound **3c** toward the *GPR119* active site, we conducted a molecular docking study using AR231453 as a positive control. Compound **3c** exhibited a docking score of −12.095 kcal/mol, representing a notably more favorable binding energy than that of AR231453 (−10.883 kcal/mol). To elucidate the binding mode, we investigated the specific interactions of compound **3c** within the *GPR119* transmembrane binding pocket. The analysis demonstrated that compound **3c** anchors stably in the active site, driven primarily by a strong hydrogen bond and π–π stacking interactions with Trp265 (see Figure 7A). Additionally, a structural superimposition of the docking poses illustrated that compound **3c** shares a highly similar binding orientation with AR231453 (see Figure 7B). The close alignment of their core scaffolds within the binding pocket further supports the validity of this binding model. Consequently, compound **3c** was selected for subsequent biological evaluations.

## 3. Materials and Methods

All chemicals were obtained from commercial suppliers and used without further purification. All solvents used for all experiments were commercially purchased and then freshly distilled from proper dehydrating agents. All solvents used for chromatography were purchased and directly applied without further purification. ^1^H-NMR and ^13^C-NMR spectra were recorded on a 400 MHz JNM-ECZ400S/L1 (JEOL, Tokyo, Japan) and a 500 MHz JNM-ECZR (JEOL, Tokyo, Japan) spectrometer. Chemical shifts are reported in parts per million (ppm) downfield relative to tetramethylsilane as an internal standard. Peak splitting patterns are abbreviated as m (multiplet), s (singlet), bs (broad singlet), d (doublet), bd (broad doublet), t (triplet), dd (doublet of doublets), ddd (doublet of doublet of doublets), dt (doublet of triplets), td (triplet of doublets), q (quartet), and quint (quintet). High-resolution mass spectra were recorded on a JMS-700 ESI mass spectrometer (JEOL, Tokyo, Japan) and Voyager DE STR MALDI-TOF mass spectrometer. Low-resolution mass spectra were recorded on API 3200 (AB SCIEX, Toronto, ON, Canada). Analytical thin-layer chromatography (TLC) was performed using a commercial glass plate with silica gel 60F 254 purchased from Merck (Darmstadt, Germany).

### 3.1. General Procedure for Synthesizing the Intermediate Compounds 2a–2d

Cyanuric chloride **1** (1.50 equiv.), an aniline derivative (200 mg, 1.00 equiv.), and anhydrous potassium carbonate (1.50 equiv.) were suspended in 10 mL of freshly distilled tetrahydrofuran (THF). The mixture was stirred under reflux conditions for 4 h. After confirming the reaction completion via thin layer chromatography (TLC), the reaction mixture was quenched by adding 10 mL of ethyl acetate, then filtered, concentrated under vacuum, and purified by flash column chromatography.

The following compounds were prepared using the procedure above, unless otherwise noted.

#### 3.1.1. 4-((4,6-Dichloro-1,3,5-triazin-2-yl)amino)-3-fluorobenzonitrile (**2a**)

The above procedure was applied for 4-amino-3-fluorobenzonitrile (200 mg, 1.47 mmol, 1.00 equiv.), cyanuric chloride **1** (406 mg, 2.20 mmol, 1.50 equiv.), and anhydrous potassium carbonate (305 mg, 2.20 mmol, 1.50 equiv.). The intermediate **2a** was obtained as a white solid (380 mg, 1.34 mmol, 92%) via flash column chromatography on silica gel (100–200) using hexane/ethyl acetate/triethylamine (79.5:20.0:0.5); ^1^H-NMR (500 MHz, CDCl_3_) *δ* 8.57 (t, *J* = 8.3 Hz, 1H, ArH), 7.85 (s, 1H, NH), 7.56 (d, *J* = 8.7 Hz, 1H, ArH), 7.47 (dd, *J* = 10.5, 1.9 Hz, 1H, ArH, H *ortho*-CF); ^13^C-NMR (126 MHz, DMSO-*d_6_*) *δ* 165.3 (s, 3C, 1,3,5-triazine), 155.0 (d, *J* = 251.2 Hz, 1C, Ar, C-F), 129.8 (d, *J* = 24.4 Hz, 1C, Ar, C *ortho*-CF), 129.7 (s, 1C, Ar), 127.8 (s, 1C, Ar), 120.8 (d, *J* = 23.4 Hz, 1C, Ar, C *ortho*-CF), 118.1 (d, *J* = 1.8 Hz, 1C, CN), 109.9 (d, *J* = 9.6 Hz, 1C, Ar, NC-C *meta*-CF); MS (ESI) calcd for C_10_H_3_Cl_2_FN_5_ [M − H]^−^ 281.98, found 282.00.

#### 3.1.2. 4,6-Dichloro-*N*-(2-fluoro-4-(methylsulfonyl)phenyl)-1,3,5-triazin-2-amine (**2b**)

The above procedure was applied for 2-fluoro-4-(methylsulfonyl)aniline (200 mg, 1.06 mmol, 1.00 equiv.), cyanuric chloride **1** (292 mg, 1.59 mmol, 1.50 equiv.), and anhydrous potassium carbonate (219 mg, 1.59 mmol, 1.50 equiv.). The intermediate **2b** was obtained as a white solid (257 mg, 0.762 mmol, yield 72%) via flash column chromatography on silica gel (100–200) using hexane/ethyl acetate/triethylamine (49.5:50.0:0.5); ^1^H-NMR (500 MHz, DMSO-*d_6_*) *δ* 11.32 (s, 1H, NH), 7.89–7.84 (m, 2H, ArH), 7.79 (dd, *J* = 8.4, 1.9 Hz, 1H, ArH), 3.26 (s, 3H, SO_2_CH_3_); ^13^C-NMR (126 MHz, DMSO-*d_6_*) *δ* 165.4 (s, 3C, 1,3,5-triazine), 155.1 (d, *J* = 254.0 Hz, 1C, C-F), 139.9 (d, *J* = 6.0 Hz, 1C, C-SO_2_CH_3_), 129.6 (d, *J* = 12.1 Hz, 1C, C *meta*-CF), 127.9 (s, 1C, Ar), 124.0 (d, *J* = 2.4 Hz, 1C, Ar), 115.8 (d, *J* = 23.0 Hz, 1C, C *ortho*-CF), 43.8 (s, 1C, SO_2_CH_3_). MS (ESI) calcd for C_10_H_6_Cl_2_FN_4_O_2_S [M − H]^−^ 334.96, found 334.93.

#### 3.1.3. 4,6-Dichloro-*N*-(2-chloro-4-(trifluoromethyl)phenyl)-1,3,5-triazin-2-amine (**2c**)

The above procedure was applied for 2-chloro-4-(trifluoromethyl)aniline (200 mg, 1.023 mmol, 1.00 equiv.), cyanuric chloride **1** (283 mg, 1.54 mmol, 1.50 equiv.), and anhydrous potassium carbonate (212 mg, 1.54 mmol, 1.50 equiv.). The intermediate **2c** was obtained as a white solid (316 mg, 0.920 mmol, 90%) via flash column chromatography on silica gel (100–200) using hexane/dichloromethane/triethylamine (49.9:50.0:0.1); ^1^H-NMR (500 MHz, CDCl_3_) *δ* 8.52 (d, *J* = 8.6 Hz, 1H, ArH), 8.05 (s, 1H, NH), 7.71 (d, *J* = 1.7 Hz, 1H, ArH), 7.63 (dd, *J* = 8.6, 1.7 Hz, 1H, ArH); ^13^C-NMR (126 MHz, CDCl_3_) *δ* 164.2 (s, 3C, 1,3,5-triazine), 136.0 (s, 1C, Ar), 127.9 (d, *J* = 33.9 Hz, 1C, Ar, C-CF_3_), 126.8 (d, *J* = 3.6 Hz, 1C, Ar, C *meta*-CCF_3_), 125.1 (d, *J* = 3.6 Hz, 1C, Ar, C *meta*-CCF_3_), 124.3 (s, 1C, Ar), 123.1 (d, *J* = 272.1 Hz, 1C, CF_3_) 122.3 (s, 1C, Ar); MS (ESI) C_10_H_5_Cl_3_F_3_N_4_: *m*/*z* [M − H]^−^ calculated 342.95, found 343.00. MS (ESI) calcd for C_10_H_3_Cl_3_F_3_N_4_ [M − H]^−^ 340.94, found 341.00.

#### 3.1.4. 4,6-Dichloro-*N*-(2-fluoro-4-(trifluoromethyl)phenyl)-1,3,5-triazin-2-amine (**2d**)

The above procedure was applied for 2-fluoro-4-(trifluoromethyl)aniline (200 mg, 1.12 mmol, 1.00 equiv.), cyanuric chloride **1** (309 mg, 1.68 mmol, 1.50 equiv.), and anhydrous potassium carbonate (232 mg, 1.68 mmol, 1.50 equiv.). The intermediate **2d** was obtained as a white solid (340 mg, 1.04 mmol, 93%) via flash column chromatography on silica gel (100–200) using hexane/dichloromethane/triethylamine (59.9:40.0:0.1); ^1^H-NMR (500 MHz, CDCl_3_) *δ* 8.48 (t, *J* = 8.2 Hz, 1H, ArH), 7.85 (s, 1H, NH), 7.51 (d, *J* = 8.4 Hz, 1H, ArH), 7.43 (dd, *J* = 10.9, 1.7 Hz, 1H, ArH, H *ortho*-CF); ^13^C-NMR (126 MHz, CDCl_3_) *δ* 164.2 (s, 3C, 1,3,5-triazine), 152.3 (d, *J* = 247.9 Hz, 1C, Ar, C-F), 128.0 (d, *J* = 9.7 Hz, 1C, Ar, C *ortho*-CF), 127.7 (dd, *J* = 26.6, 7.3 Hz, 1C, C C-CF_3_ *meta*-CF), 124.2 (m, 1C, CF_3_), 122.5 (s, 1C, Ar), 122.2 (t, *J* = 3.6 Hz, 1C, Ar, C *meta*-CF), 113.0 (dd, *J* = 23.0, 3.6 Hz, 1C, C *ortho*-C-CF_3_ *ortho*-CF); MS (ESI) calcd for C_10_H_3_Cl_2_F_4_N_4_ [M − H]^−^ 324.97, found 324.80.

### 3.2. General Procedure for the Targeted Compounds 3a–3p

The intermediate compound (1.01 equiv.), corresponding amine (150 mg, 1.00 equiv.), and diisopropylethylamine (1.01 equiv.) were suspended in 5 mL of freshly distilled tetrahydrofuran (THF). The mixture was stirred under reflux conditions for 6 h. After confirming the reaction completion via thin layer chromatography (TLC), the reaction mixture was quenched by adding 20 mL ethyl acetate, then filtered, concentrated under vacuum, and purified by flash chromatography.

The following compounds were prepared using the procedure above, unless otherwise noted.

#### 3.2.1. (1*R*,5*S*)-*tert*-butyl 8-(4-chloro-6-((4-cyano-2-fluorophenyl)amino)-1,3,5-triazin-2-yl)-3,8-diazabicyclo[3.2.1]octane-3-carboxylate (**3a**)

The above procedure was applied for intermediate **2a** (205 mg, 0.717 mmol, 1.01 equiv.), (1*R*,5*S*)-*tert*-butyl 3,8-diazabicyclo[3.2.1]octane-3-carboxylate (150 mg, 0.707 mmol, 1.00 equiv.), and diisopropylethylamine (0.124 mL, 0.717 mmol, 1.01 equiv.). The product **3a** was obtained as a white solid (197 mg, 0.428 mmol, 61%) via flash column chromatography on silica gel (100–200) using hexane/ethyl acetate/triethylamine (66.6:33.3:0.1); ^1^H-NMR (500 MHz, CDCl_3_) *δ* 8.53 (t, *J* = 8.2 Hz, 1H, ArH), 7.48 (d, *J* = 8.4 Hz, 1H, ArH), 7.43 (s, 1H, NH), 7.39 (dd, *J* = 10.7, 1.9 Hz, 1H, ArH, H *ortho*-CF), 4.84–4.79 (m, 1H, NCH), 4.71–4.67 (m, 1H, NCH), 4.00–3.97 (m, 1H, NCH_2_), 3.88–3.79 (m, 1H, NCH_2_), 3.15–3.12 (m, 1H, NCH_2_), 3.05–3.01 (m, 1H, NCH_2_), 1.99 (d, *J* = 6.5 Hz, 2H, CCH_2_), 1.91–1.83 (m, 2H, CCH_2_), 1.45 (s, 9H, Boc); ^13^C-NMR (126 MHz, CDCl_3_) *δ* 170.1 (s, 1C, 1,3,5-triazine), 163.8 (s, 1C, 1,3,5-triazine), 161.8 (s, 1C, 1,3,5-triazine), 156.0–155.8 (m, 1C, Boc), 151.3 (d, *J* = 247.9 Hz, 1C, Ar, C-F), 131.6 (d, *J* = 8.5 Hz, 1C, Ar, C *meta*-CF), 129.4 (s, 1C, Ar), 121.2 (s, 1C, Ar), 118.6 (d, *J* = 23.0 Hz, 1C, Ar, C *ortho*-CF), 117.9 (s, 1C, CN), 106.0 (d, *J* = 3.6 Hz, 1C, NC-C *meta*-CF), 80.5 (s, 1C, Boc), 53.7–53.2 (m, 2C, NCH), 50.2–48.6 (m, 2C, NCH_2_), 28.5 (s, 3C, Boc), 26.7 (s, 2C, CCH_2_); MS (ESI) calcd for C_21_H_24_ClFN_7_O_2_ [M + H]^+^ 460.17, found 460.10; for C_21_H_22_ClFN_7_O_2_ [M − H]^−^ 458.15, found 458.30.

#### 3.2.2. (1*R*,5*S*)-*tert*-butyl 3-(4-chloro-6-((4-cyano-2-fluorophenyl)amino)-1,3,5-triazin-2-yl)-3,8-diazabicyclo[3.2.1]octane-8-carboxylate (**3b**)

The above procedure was applied for intermediate **2a** (205 mg, 0.714 mmol, 1.01 equiv.), (1*R*,5*S*)-*tert*-butyl 3,8-diazabicyclo[3.2.1]octane-8-carboxylate (150 mg, 0.707 mmol, 1.00 equiv.), and diisopropylethylamine (0.124 mL, 0.714 mmol, 1.01 equiv.). The product **3b** was obtained as a white solid (170 mg, 0.370 mmol, 52%) via flash column chromatography on silica gel (100–200) using hexane/ethyl acetate/triethylamine (66.6:33.3:0.1); ^1^H-NMR (500 MHz, CDCl_3_) *δ* 8.51 (t, *J* = 7.8 Hz, 1H, ArH), 7.48 (d, *J* = 8.8 Hz, 1H, ArH), 7.45 (s, 1H, NH), 7.39 (dd, *J* = 10.7, 1.9 Hz, 1H, ArH, H *ortho*-CF), 4.47–4.28 (m, 4H, NCH, NCH_2_), 3.26–3.19 (m, 2H, NCH_2_), 1.99–1.92 (m, 2H, CCH_2_), 1.67–1.60 (m, 2H, CCH_2_), 1.48 (s, 9H, Boc); ^13^C-NMR (126 MHz, CDCl_3_) *δ* 169.8 (s, 1C, 1,3,5-triazine), 166.1 (s, 1C, 1,3,5-triazine), 163.5 (s, 1C, 1,3,5-triazine), 153.5 (s, 1C, Boc), 151.4 (d, *J* = 247.9 Hz, 1C, Ar, C-F), 131.5 (d, *J* = 9.7 Hz, 1C, Ar, C *meta*-CF), 129.4 (d, *J* = 2.4 Hz, 1C, Ar), 121.3 (s, 1C, Ar), 118.6 (d, *J* = 23.0 Hz, 1C, Ar, C *ortho*-CF), 117.9 (s, 1C, CN), 106.1 (d, *J* = 9.7 Hz, 1C, Ar, C *meta*-CF), 80.4 (s, 1C, Boc), 53.5–52.5 (m, 2C, NCH), 49.9–49.5 (m, 2C, NCH_2_), 28.5 (s, 3C, Boc), 27.7–26.6 (m, 2C, CCH_2_); MS (ESI) calcd for C_21_H_24_ClFN_7_O_2_ [M + H]^+^ 460.17, found 460.10; for C_21_H_22_ClFN_7_O_2_ [M − H]^−^ 458.15, found 458.10.

#### 3.2.3. *tert*-butyl 4-((4-chloro-6-((4-cyano-2-fluorophenyl)amino)-1,3,5-triazin-2-yl)amino)piperidine-1-carboxylate (**3c**)

The above procedure was applied for intermediate **2a** (215 mg, 0.756 mmol, 1.01 equiv.), *tert*-butyl 4-aminopiperidine-1-carboxylate (150 mg, 0.749 mmol, 1.00 equiv.), and diisopropylethylamine (0.132 mL, 0.756 mmol, 1.01 equiv.). The product **3c** was obtained as a white solid (237 mg, 0.529 mmol, 71%) via flash column chromatography on silica gel (100–200) using hexane/ethyl acetate/triethylamine (66.6:33.3:0.1); ^1^H-NMR (500 MHz, CDCl_3_) (mixture of rotamers) *δ* 8.60–8.55 (m, 1H, ArH), 7.57–7.49 (m, 1H, NH), 7.46–7.41 (m, 1H, ArH), 7.41–7.35 (m, 1H, ArH), 5.73–5.53 (m, 1H, NH piperidine), 4.12–4.05 (m, 2H, NCH_2_), 3.98–3.91 (m, 1H, NCH), 2.93 (t, *J* = 11.4 Hz, 2H, NCH_2_), 2.00 (dd, *J* = 12.4, 2.5 Hz, 2H, CCH_2_), 1.48–1.32 (m, 11H, CCH_2_, Boc); ^13^C-NMR (126 MHz, CDCl_3_) (mixture of rotamers) *δ* 170.3–169.6 (m, 1C, 1,3,5-triazine), 165.2–165.1 (m, 1C, 1,3,5-triazine), 164.0–163.5 (m, 1C, 1,3,5-triazine), 154.8–154.7 (m, 1C, Boc), 151.4 (d, *J* = 250.4 Hz, 1C, Ar, C-F), 131.4 (d, *J* = 9.7 Hz, 1C, Ar, C *meta*-CF), 129.2 (s, 1C, Ar), 121.6–121.5 (m, 1C, Ar), 118.6 (d, *J* = 21.8 Hz, 1C, Ar, C *ortho*-CF), 117.8 (s, 1C, CN), 106.3 (d, *J* = 8.5 Hz, 1C, Ar, C *meta*-CF), 80.1–80.0 (m, 1C, Boc), 48.9–48.5 (m, 2C, NCH_2_), 42.9–42.1 (m, 1C, NCH), 32.0–31.5 (m, 2C, CCH_2_), 28.5 (s, 3C, Boc). HRMS (ESI) calcd for C_20_H_23_ClFN_7_O_2_ [M]^+^ 447.1586; found, 447.1585.

#### 3.2.4. *tert*-butyl 4-(4-chloro-6-((4-cyano-2-fluorophenyl)amino)-1,3,5-triazin-2-yl)piperazine-1-carboxylate (**3d**)

The above procedure was applied for intermediate **2a** (231 mg, 0.813 mmol, 1.01 equiv.), *tert*-butyl piperazine-1-carboxylate (150 mg, 0.805 mmol, 1.00 equiv.), and diisopropylethylamine (0.142 mL, 0.813 mmol, 1.01 equiv.). The product **3d** was obtained as a white solid (175 mg, 0.403 mmol, 50%) via flash column chromatography on silica gel (100–200) using hexane/ethyl acetate/triethylamine (74.9:25.0:0.1); ^1^H-NMR (400 MHz, CDCl_3_) *δ* 8.51 (t, *J* = 8.3 Hz, 1H, ArH), 7.48 (d, *J* = 8.6 Hz, 1H, ArH), 7.45 (s, 1H, NH), 7.39 (dd, *J* = 10.7, 1.5 Hz, 1H, ArH, H *ortho*-CF), 3.86 (t, *J* = 9.8 Hz, 2H, NCH_2_), 3.80 (t, *J* = 9.8 Hz, 2H, NCH_2_), 3.51 (quint, *J* = 5.1 Hz, 4H, NCH_2_), 1.47 (s, 9H, Boc); ^13^C-NMR (101 MHz, CDCl_3_) *δ* 170.0 (s, 1C, 1,3,5-triazine), 164.5 (s, 1C, 1,3,5-triazine), 163.7 (s, 1C, 1,3,5-triazine), 154.6 (s, 1C, Boc), 151.4 (d, *J* = 248.7 Hz, Ar, C-F), 131.5 (d, *J* = 9.7 Hz, 1C, Ar, C *meta*-CF), 129.4 (d, *J* = 2.9 Hz, Ar), 121.3 (s, 1C, Ar), 118.6 (d, *J* = 23.3 Hz, 1C, Ar, C *ortho*-CF), 117.9 (d, *J* = 1.9 Hz, CN), 106.1 (d, *J* = 8.7 Hz, 1C, Ar), 80.6 (s, 1C, Boc), 43.9 (s, 2C, NCH_2_), 43.7 (s, 2C, NCH_2_), 28.5 (s, 3C, Boc). HRMS (ESI) calcd for C_19_H_21_ClFN_7_O_2_ [M]^+^ 433.1429; found, 433.1430.

#### 3.2.5. (1*R*,5*S*)-*tert*-butyl 8-(4-chloro-6-((2-fluoro-4-(methylsulfonyl)phenyl)amino)-1,3,5-triazin-2-yl)-3,8-diazabicyclo[3.2.1]octane-3-carboxylate (**3e**)

The above procedure was applied for intermediate **2b** (241 mg, 0.714 mmol, 1.01 equiv.), (1*R*,5*S*)-*tert*-butyl 3,8-diazabicyclo[3.2.1]octane-3-carboxylate (150 mg, 0.707 mmol, 1.00 equiv.), and diisopropylethylamine (0.124 mL, 0.714 mmol, 1.01 equiv.). The product **3e** was obtained as a white solid (241 mg, 0.470 mmol, 66%) via flash column chromatography on silica gel (100–200) using hexane/ethyl acetate/triethylamine (66.6:33.3:0.1); ^1^H-NMR (500 MHz, CDCl_3_) *δ* 8.60 (t, *J* = 8.0 Hz, 1H, ArH), 7.75 (d, *J* = 8.4 Hz, 1H, ArH), 7.67 (dd, *J* = 10.1, 2.1 Hz, 1H, ArH, H *ortho*-CF), 7.47 (s, 1H, NH), 4.83–4.79 (m, 1H, NCH), 4.71–4.68 (m, 1H, NCH), 3.99–3.97 (m, 1H, NCH_2_), 3.87–3.79 (m, 1H, NCH_2_), 3.14–3.01 (m, 5H, NCH_2_, SO_2_CH_3_), 2.01–1.98 (m, 2H, CCH_2_), 1.87 (d, *J* = 15.6 Hz, 2H, CCH_2_), 1.45 (s, 9H, Boc); ^13^C-NMR (126 MHz, CDCl_3_) (mixture of rotamers) *δ* 170.1 (s, 1C, 1,3,5-triazine), 163.9 (s, 1C, 1,3,5-triazine), 161.9 (s, 1C, 1,3,5-triazine), 155.9–155.8 (m, 1C, Boc), 151.5 (d, *J* = 251.6 Hz, 1C, Ar, C-F), 134.7 (d, *J* = 4.8 Hz, 1C, Ar, C-SO_2_CH_3_), 132.0 (d, *J* = 8.5 Hz, 1C, Ar, C *meta*-CF), 124.4 (s, 1C, Ar), 121.3 (s, 1C, Ar), 114.5 (d, *J* = 23.0 Hz, 1C, Ar, C *ortho*-CF), 80.4 (s, 1C, Boc), 53.6–53.3 (m, 2C, NCH), 50.2–48.6 (m, 2C, NCH_2_), 44.7 (s, 1C, SO_2_CH_3_), 28.5 (s, 3C, Boc), 26.9–26.6 (m, 2C, CCH_2_); MS (ESI) calcd for C_21_H_25_ClFN_6_O_4_S [M − H]^−^ 511.13, found 511.00.

#### 3.2.6. (1*R*,5*S*)-*tert*-butyl 3-(4-chloro-6-((2-fluoro-4-(methylsulfonyl)phenyl)amino)-1,3,5-triazin-2-yl)-3,8-diazabicyclo[3.2.1]octane-8-carboxylate (**3f**)

The above procedure was applied for intermediate **2b** (241 mg, 0.714 mmol, 1.01 equiv.), (1*R*,5*S*)-*tert*-butyl 3,8-diazabicyclo[3.2.1]octane-8-carboxylate (150 mg, 0.707 mmol, 1.00 equiv.), and diisopropylethylamine (0.124 mL, 0.714 mmol, 1.01 equiv.). The product **3f** was obtained as a white solid (229 mg, 0.446 mmol, 63%) via flash column chromatography on silica gel (100–200) using hexane/ethyl acetate/triethylamine (59.9:40.0:0.1); ^1^H-NMR (400 MHz, CDCl_3_) *δ* 8.58 (t, *J* = 7.9 Hz, 1H, ArH), 7.76 (d, *J* = 8.6 Hz, 1H, ArH), 7.68 (dd, *J* = 9.8, 1.8 Hz, 1H, Ar, H *ortho*-CF), 7.47 (s, 1H, NH), 4.48–4.33 (m, 4H, NCH, NCH_2_), 3.22–3.18 (m, 2H, NCH_2_), 3.05 (s, 3H, SO_2_CH_3_), 1.96–1.91 (m, 2H, CCH_2_), 1.68–1.63 (m, 2H, CCH_2_), 1.48 (s, 9H, Boc); ^13^C-NMR (101 MHz, CDCl_3_) *δ* 169.8 (s, 1C, 1,3,5-triazine), 166.2 (s, 1C, 1,3,5-triazine), 163.5 (s, 1C, 1,3,5-triazine), 153.5 (s, 1C, Boc), 151.5 (d, *J* = 250.7 Hz, 1C, Ar, C-F), 134.7 (d, *J* = 5.8 Hz, 1C, Ar, C-SO_2_CH_3_), 132.0 (d, *J* = 9.7 Hz, 1C, Ar, C *meta*-CF), 124.4 (d, *J* = 3.9 Hz, 1C, Ar), 121.3 (s, 1C, Ar), 114.6 (d, *J* = 22.3 Hz, 1C, Ar, C *ortho*-CF), 80.4 (s, 1C, Boc), 53.6–52.6 (m, 2C, NCH), 49.9–49.5 (m, 2C, NCH_2_), 44.7 (s, 1C, SO_2_CH_3_), 28.5 (s, 3C, Boc), 27.6–26.7 (m, 2C, CCH_2_); MS (ESI) calcd for C_21_H_25_ClFN_6_O_4_S [M − H]^−^ 511.13, found 511.09.

#### 3.2.7. *tert*-butyl 4-((4-chloro-6-((2-fluoro-4-(methylsulfonyl)phenyl)amino)-1,3,5-triazin-2-yl)amino)piperidine-1-carboxylate (**3g**)

The above procedure was applied for intermediate **2b** (255 mg, 0.749 mmol, 1.01 equiv.), *tert*-butyl 4-aminopiperidine-1-carboxylate (150 mg, 0.749 mmol, 1.00 equiv.), and diisopropylethylamine (0.132 mL, 0.756 mmol, 1.01 equiv.). The product **3g** was obtained as a white solid (211 mg, 0.421 mmol, 56%) via flash column chromatography on silica gel (100–200) using hexane/ethyl acetate/triethylamine (66.6:33.3:0.1); ^1^H-NMR (400 MHz, CDCl_3_) (mixture of rotamers) *δ* 8.66 (td, *J* = 8.1, 4.7 Hz, 1H, ArH), dt7.74–7.64 (m, 2H, ArH), 7.59–7.50 (m, 1H, NH), 5.76–5.57 (m, 1H, NH piperidine), 4.13–3.93 (m, 3H, NCH, NCH_2_), 3.06–3.04 (m, 3H, SO_2_CH_3_), 2.98–2.89 (m, 2H, NCH_2_), 2.02–1.99 (m, 2H, CCH_2_), 1.45–1.42 (m, 11H, CCH_2_, Boc); ^13^C-NMR (101 MHz, CDCl_3_) (mixture of rotamers) *δ* 170.3–169.6 (m, 1C, 1,3,5-triazine), 165.3–165.1 (m, 1C, 1,3,5-triazine), 164.1–163.6 (m, 1C, 1,3,5-triazine), 154.8–154.7 (m, 1C, Boc), 151.5 (d, *J* = 250.7 Hz, 1C, Ar, C-F), 134.9–134.6 (m, 1C, Ar), 131.9–131.8 (m, 1C, Ar), 124.3 (s, 1C, Ar), 121.6–121.2 (m, 1C, Ar), 114.8–114.4 (m, 1C, Ar), 80.1–80.0 (m, 1C, Boc), 48.9–48.5 (m, 2C, NCH_2_), 44.8 (s, 1C, SO_2_CH_3_), 42.8–42.3 (m, 1C, NCH), 32.0–31.5 (m, 2C, CCH_2_), 28.5 (s, 3C, Boc); HRMS (EI-MS) calcd for C_20_H_26_ClFN_6_O_4_S [M]^+^ 500.1409; found, 500.1404.

#### 3.2.8. *tert*-butyl 4-(4-chloro-6-((2-fluoro-4-(methylsulfonyl)phenyl)amino)-1,3,5-triazin-2-yl)piperazine-1-carboxylate (**3h**)

The above procedure was applied for intermediate **2b** (274 mg, 0.813 mmol, 1.01 equiv.), *tert*-butyl piperazine-1-carboxylate (150 mg, 0.805 mmol, 1.00 equiv.), and diisopropylethylamine (0.142 mL, 0.813 mmol, 1.01 equiv.). The product **3h** was obtained as a white solid (248 mg, 0.509 mmol, 63%) via flash column chromatography on silica gel (100–200) using hexane/ethyl acetate/triethylamine (59.9:40.0:0.1); ^1^H-NMR (400 MHz, CDCl_3_) *δ* 8.58 (t, *J* = 7.9 Hz, 1H, ArH), 7.75 (d, *J* = 9.2 Hz, 1H, ArH), 7.68 (dd, *J* = 10.4, 1.8 Hz, 1H, ArH, H *ortho*-CF), 7.48 (s, 1H, NH), 3.86–3.80 (m, 4H, NCH_2_), 3.53–3.48 (m, 4H, NCH_2_), 3.05 (s, 3H, SO_2_CH_3_), 1.47 (s, 9H, Boc); ^13^C-NMR (101 MHz, CDCl_3_) *δ* 170.0 (s, 1C, 1,3,5-triazine), 164.5 (s, 1C, 1,3,5-triazine), 163.8 (s, 1C, 1,3,5-triazine), 154.6 (s, 1C, Boc), 151.6 (d, *J* = 251.6 Hz, 1C, Ar, C-F), 134.7 (d, *J* = 5.8 Hz, 1C, Ar, C-SO_2_CH_3_), 131.9 (d, *J* = 9.7 Hz, 1C, Ar, C *meta*-CF), 124.4 (d, *J* = 2.9 Hz, 1C, Ar), 121.3 (s, 1C, Ar), 114.6 (d, *J* = 22.3 Hz, 1C, Ar, C *ortho*-CF), 80.6 (s, 1C, Boc), 44.7 (s, 1C, SO_2_CH_3_), 43.9 (s, 2C, NCH_2_), 43.7 (s, 2C, NCH_2_), 28.5 (s, 3C, Boc); MS (ESI) calcd for C_19_H_23_ClFN_6_O_4_S [M − H]^−^ 485.12, found 485.07.

#### 3.2.9. (1*R*,5*S*)-*tert*-butyl 8-(4-chloro-6-((2-chloro-4-(trifluoromethyl)phenyl)amino)-1,3,5-triazin-2-yl)-3,8-diazabicyclo[3.2.1]octane-3-carboxylate (**3i**)

The above procedure was applied for intermediate **2c** (245 mg, 0.714 mmol, 1.01 equiv.), (1*R*,5*S*)-*tert*-butyl 3,8-diazabicyclo[3.2.1]octane-3-carboxylate (150 mg, 0.707 mmol, 1.00 equiv.), and diisopropylethylamine (0.124 mL, 0.714 mmol, 1.01 equiv.). The product **3i** was obtained as a white solid (227 mg, 0.437 mmol, 62%) via flash column chromatography on silica gel (100–200) using hexane/ethyl acetate/triethylamine (89.9:10.0:0.1); ^1^H-NMR (500 MHz, CDCl_3_) *δ* 8.54 (d, *J* = 8.4 Hz, 1H, ArH), 7.65 (d, *J* = 1.5 Hz, 1H, ArH), 7.61 (s, 1H, NH), 7.55 (dd, *J* = 8.8, 1.9 Hz, 1H, ArH), 4.83–4.78 (m, 1H, NCH), 4.72–4.69 (m, 1H, NCH), 3.99–3.97 (m, 1H, NCH_2_), 3.87–3.79 (m, 1H, NCH_2_), 3.14 (d, *J* = 12.6 Hz, 1H, NCH_2_), 3.04 (t, *J* = 10.9 Hz, 1H, NCH_2_), 1.99 (d, *J* = 6.1 Hz, 2H, CCH_2_), 1.90–1.84 (m, 2H, CCH_2_), 1.45 (s, 9H, Boc); ^13^C-NMR (126 MHz, CDCl_3_) (mixture of rotamers) *δ* 170.1 (s, 1C, 1,3,5-triazine), 163.9 (s, 1C, 1,3,5-triazine), 161.9 (s, 1C, 1,3,5-triazine), 156.0–155.9 (m, 1C, Boc), 137.9 (s, 1C, Ar), 126.6 (d, *J* = 3.6 Hz, Ar, C *meta*-CCF_3_), 126.2–125.7 (m, 1C, Ar), 124.7 (s, 1C, Ar), 123.4 (d, *J* = 272.1 Hz, 1C, CF_3_), 123.1 (s, 1C, Ar), 121.1 (s, 1C, Ar), 80.4 (s, 1C, Boc), 53.6–53.1 (m, 2C, NCH), 50.2–48.6 (m, 2C, NCH_2_), 28.5 (s, 3C, Boc), 26.7–26.6 (m, 2C, CCH_2_); MS (ESI) calcd for C_21_H_22_Cl_2_F_3_N_6_O_2_ [M − H]^−^ 517.11, found 517.02.

#### 3.2.10. (1*R*,5*S*)-*tert*-butyl 3-(4-chloro-6-((2-chloro-4-(trifluoromethyl)phenyl)amino)-1,3,5-triazin-2-yl)-3,8-diazabicyclo[3.2.1]octane-8-carboxylate (**3j**)

The above procedure was applied for intermediate **2c** (245 mg, 0.714 mmol, 1.01 equiv.), (1*R*,5*S*)-*tert*-butyl 3,8-diazabicyclo[3.2.1]octane-8-carboxylate (150 mg, 0.707 mmol, 1.00 equiv.), and diisopropylethylamine (0.124 mL, 0.714 mmol, 1.01 equiv.). The product **3j** was obtained as a white solid (201 mg, 0.387 mmol, 55%) via flash column chromatography on silica gel (100–200) using hexane/ethyl acetate/triethylamine (89.9:10.0:0.1); ^1^H-NMR (500 MHz, CDCl_3_) *δ* 8.51 (d, *J* = 8.8 Hz, 1H, ArH), 7.65 (d, *J* = 1.5 Hz, 1H, ArH), 7.61 (s, 1H, NH), 7.56 (d, *J* = 8.8 Hz, 1H, ArH), 4.48–4.31 (m, 4H, NCH, NCH_2_), 3.20–3.18 (m, 2H, NCH_2_), 1.99–1.91 (m, 2H, CCH_2_), 1.70–1.60 (m, 2H, CCH_2_), 1.48 (s, 9H, Boc); ^13^C-NMR (126 MHz, CDCl_3_) (mixture of rotamers) *δ* 169.7 (s, 1C, 1,3,5-triazine), 166.2 (s, 1C, 1,3,5-triazine), 163.6 (s, 1C, 1,3,5-triazine), 153.5 (s, 1C, Boc), 137.9 (s, 1C, Ar), 126.6 (d, *J* = 4.8 Hz, 1C, Ar, C *meta*-CCF_3_), 125.9 (d, *J* = 33.9 Hz, 1C, Ar, C *ortho*-CCF_3_), 124.7 (d, *J* = 3.6 Hz, 1C, Ar, C *meta*-CCF_3_), 123.4 (d, *J* = 272.1 Hz, 1C, CF_3_), 123.2 (s, 1C, Ar), 121.1 (s, 1C, Ar), 80.3 (s, 1C, Boc), 53.6–52.6 (m, 2C, NCH), 49.8–49.4 (m, 2C, NCH_2_), 28.5 (s, 3C, Boc), 27.7–26.8 (m, 2C, CCH_2_); MS (ESI) calcd for C_21_H_22_Cl_2_F_3_N_6_O_2_ [M − H]^−^ 517.11, found 511.12.

#### 3.2.11. *tert*-butyl 4-((4-chloro-6-((2-chloro-4-(trifluoromethyl)phenyl)amino)-1,3,5-triazin-2-yl)amino)piperidine-1-carboxylate (**3k**)

The above procedure was applied for intermediate **2c** (260 mg, 0.756 mmol, 1.01 equiv.), *tert*-butyl 4-aminopiperidine-1-carboxylate (150 mg, 0.749 mmol, 1.00 equiv.), and diisopropylethylamine (0.132 mL, 0.756 mmol, 1.01 equiv.). The product **3k** was obtained as a white solid (188 mg, 0.371 mmol, 74%) via flash column chromatography on silica gel (100–200) using hexane/ethyl acetate/triethylamine (85.6:14.3:0.1); ^1^H-NMR (400 MHz, CDCl_3_) (mixture of rotamers) *δ* 8.58 (t, *J* = 9.8 Hz, 1H, ArH), 7.68 (d, *J* = 10.4 Hz, 1H, ArH), 7.64–7.58 (m, 1H, NH), 7.52 (d, *J* = 8.6 Hz, 1H, ArH), 5.56–5.38 (m, 1H, NH piperidine), 4.14–4.06 (m, 2H, NCH_2_), 4.01–3.93 (m, 1H, NCH), 2.98–2.89 (m, 2H, NCH_2_), 2.03–2.00 (m, 2H, CCH_2_), 1.49–1.38 (m, 11H, CCH_2_, Boc); ^13^C-NMR (101 MHz, CDCl_3_) (mixture of rotamers) *δ* 170.3–169.6 (m, 1C, 1,3,5-triazine), 165.2–165.1 (m, 1C, 1,3,5-triazine), 164.1–163.6 (m, 1C, 1,3,5-triazine), 154.8–154.7 (m, 1C, Boc), 137.7 (s, 1C, Ar), 126.7–126.5 (m, 1C, Ar), 126.3–125.8 (m, 1C, Ar), 124.6–124.5 (m, 1C, Ar), 123.4 (d, *J* = 272.9 Hz, 1C, CF_3_), 123.3–123.2 (m, 1C, Alkyl), 121.5–121.2 (m, 1C, Alkyl), 80.1–80.0 (m, 1C, Boc), 48.8–48.4 (m, 2C, NCH_2_), 43.0–42.2 (m, 1C, NCH), 32.1–31.6 (m, 2C, CCH_2_), 28.5 (s, 3C, Boc); MS (ESI) calcd for C_20_H_22_Cl_2_F_3_N_6_O_2_ [M − H]^−^ 505.11, found 505.16.

#### 3.2.12. *tert*-butyl 4-(4-chloro-6-((2-chloro-4-(trifluoromethyl)phenyl)amino)-1,3,5-triazin-2-yl)piperazine-1-carboxylate (**3l**)

The above procedure was applied for intermediate **2c** (279 mg, 0.813 mmol, 1.01 equiv.), *tert*-butyl piperazine-1-carboxylate (150 mg, 0.805 mmol, 1.00 equiv.), and diisopropylethylamine (0.142 mL, 0.813 mmol, 1.01 equiv.). The product **3l** was obtained as a white solid (168 mg, 0.341 mmol, 42%) via flash column chromatography on silica gel (100–200) using hexane/ethyl acetate/triethylamine (85.6:14.3:0.1); ^1^H-NMR (400 MHz, CDCl_3_) *δ* 8.51 (d, *J* = 8.6 Hz, 1H, ArH), 7.65 (s, 1H, ArH), 7.60 (s, 1H, NH), 7.56 (d, *J* = 9.2 Hz, 1H, ArH), 3.86–3.79 (m, 4H, NCH_2_), 3.53–3.50 (m, 4H, NCH_2_), 1.48 (s, 9H, Boc); ^13^C-NMR (101 MHz, CDCl_3_) *δ* 170.0 (s, 1C, 1,3,5-triazine), 164.6 (s, 1C, 1,3,5-triazine), 163.8 (s, 1C, 1,3,5-triazine), 154.6 (s, 1C, Boc), 137.8 (s, 1C, Ar), 126.6 (d, *J* = 3.9 Hz, 1C, Ar, C *meta*-CCF_3_), 125.9 (d, *J* = 33.9 Hz, 1C, Ar, C *ortho*-CCF_3_), 124.9 (d, *J* = 2.9 Hz, 1C, Ar, C *meta*-CCF_3_), 123.4 (d, *J* = 272.9 Hz, 1C, CF_3_), 123.2 (s, 1C, Ar), 121.2 (s, 1C, Ar), 80.6 (s, 1C, Boc), 43.8 (s, 2C, NCH_2_), 43.7 (s, 2C, NCH_2_), 28.5 (s, 3C, Boc); MS (ESI) calcd for C_19_H_20_Cl_2_F_3_N_6_O_2_ [M − H]^−^ 491.10, found 491.04.

#### 3.2.13. (1*R*,5*S*)-*tert*-butyl 8-(4-chloro-6-((2-fluoro-4-(trifluoromethyl)phenyl)amino)-1,3,5-triazin-2-yl)-3,8-diazabicyclo[3.2.1]octane-3-carboxylate (**3m**)

The above procedure was applied for intermediate **2d** (234 mg, 0.714 mmol, 1.01 equiv.), (1*R*,5*S*)-*tert*-butyl 3,8-diazabicyclo[3.2.1]octane-3-carboxylate (150 mg, 0.707 mmol, 1.00 equiv.), and diisopropylethylamine (0.124 mL, 0.714 mmol, 1.01 equiv.). The product **3m** was obtained as a white solid (253 mg, 0.503 mmol, 71%) via flash column chromatography on silica gel (100–200) using hexane/ethyl acetate/triethylamine (89.9:10.0:0.1); ^1^H-NMR (500 MHz, CDCl_3_) *δ* 8.48 (t, *J* = 8.0 Hz, 1H, ArH), 7.44 (d, *J* = 8.8 Hz, 1H, ArH), 7.37–7.35 (m, 2H, ArH, NH), 4.83–4.78 (m, 1H, NCH), 4.72–4.68 (m, 1H, NCH), 3.99–3.97 (m, 1H, NCH_2_), 3.87–3.78 (m, 1H, NCH_2_), 3.14 (d, *J* = 12.6 Hz, 1H, NCH_2_), 3.04 (t, *J* = 11.3 Hz, 1H, NCH_2_), 1.99 (d, *J* = 5.7 Hz, 2H, CCH_2_), 1.90–1.83 (m, 2H, CCH_2_), 1.45 (s, 9H, Boc); ^13^C-NMR (126 MHz, CDCl_3_) (mixture of rotamers) *δ* 170.0 (s, 1C, 1,3,5-triazine), 163.9 (s, 1C, 1,3,5-triazine), 162.0 (s, 1C, 1,3,5-triazine), 156.0–155.9 (m, 1C, Boc), 151.8 (d, *J* = 246.7 Hz, 1C, Ar, C-F), 130.0–129.9 (m, 1C, Ar), 125.5 (m, 1C, Ar), 123.4 (d, *J* = 272.1 Hz, 1C, CF_3_), 121.8 (s, 1C, Ar), 121.2 (s, 1C, Ar), 112.6 (d, *J* = 21.8 Hz, 1C, Ar, C *ortho*-CF), 80.4 (s, 1C, Boc), 53.7–53.1 (m, 2C, NCH), 50.2–48.6 (m, 2C, NCH_2_), 28.5 (s, 3C, Boc), 26.7–26.6 (m, 2C, CCH_2_); MS (ESI) calcd for C_21_H_22_ClF_4_N_6_O_2_ [M − H]^−^ 501.14, found 501.15.

#### 3.2.14. (1*R*,5*S*)-*tert*-butyl 3-(4-chloro-6-((2-fluoro-4-(trifluoromethyl)phenyl)amino)-1,3,5-triazin-2-yl)-3,8-diazabicyclo[3.2.1]octane-8-carboxylate (**3n**)

The above procedure was applied for intermediate **2d** (234 mg, 0.714 mmol, 1.01 equiv.), (1*R*,5*S*)-*tert*-butyl 3,8-diazabicyclo[3.2.1]octane-8-carboxylate (150 mg, 0.707 mmol, 1.00 equiv.), and diisopropylethylamine (0.124 mL, 0.714 mmol, 1.01 equiv.). The product **3n** was obtained as a white solid (340 mg, 0.676 mmol, 96%) via flash column chromatography on silica gel (100–200) using hexane/ethyl acetate/triethylamine (89.9:10.0:0.1); ^1^H-NMR (500 MHz, CDCl_3_) *δ* 8.44 (t, *J* = 7.8 Hz, 1H, ArH), 7.44 (d, *J* = 8.4 Hz, 1H, ArH), 7.40 (s, 1H, NH), 7.36 (dd, *J* = 11.1, 1.5 Hz, 1H, ArH, H *ortho*-CF), 4.48–4.31 (m, 4H, NCH, NCH_2_), 3.33–3.10 (m, 2H, NCH_2_), 1.95 (t, *J* = 5.9 Hz, 2H, CCH_2_), 1.70–1.63 (m, 2H, CCH_2_), 1.48 (s, 9H, Boc); ^13^C-NMR (126 MHz, CDCl_3_) (mixture of rotamers) *δ* 169.7 (s, 1C, 1,3,5-triazine), 166.2 (s, 1C, 1,3,5-triazine), 163.6 (s, 1C, 1,3,5-triazine), 153.5 (s, 1C, Boc), 151.9 (d, *J* = 246.7 Hz, 1C, Ar, C-F), 129.9 (d, *J* = 9.7 Hz, 1C, Ar, C *ortho*-CF), 125.4 (d, *J* = 6.0 Hz, 1C, Ar), 123.4 (d, *J* = 270.9 Hz, 1C, CF_3_), 121.8 (s, 1C, Ar), 121.3 (s, 1C, Ar), 112.6 (d, *J* = 26.6 Hz, 1C, Ar, C *ortho*-CF), 80.3 (s, 1C, Boc), 53.6–52.7 (m, 2C, NCH), 49.9–49.5 (m, 2C, NCH_2_), 28.5 (s, 3C, Boc), 27.5–26.6 (m, 2C, CCH_2_); MS (ESI) calcd for C_21_H_22_ClF_4_N_6_O_2_ [M − H]^−^ 501.14, found 501.13.

#### 3.2.15. *tert*-butyl 4-((4-chloro-6-((2-fluoro-4-(trifluoromethyl)phenyl)amino)-1,3,5-triazin-2-yl)amino)piperidine-1-carboxylate (**3o**)

The above procedure was applied for intermediate **2d** (247 mg, 0.756 mmol, 1.01 equiv.), *tert*-butyl 4-aminopiperidine-1-carboxylate (150 mg, 0.749 mmol, 1.00 equiv.), and diisopropylethylamine (0.132 mL, 0.756 mmol, 1.01 equiv.). The product **3o** was obtained as a white solid (268 mg, 0.546 mmol, 73%) via flash column chromatography on silica gel (100–200) using hexane/ethyl acetate/triethylamine (66.6:33.3:0.1); ^1^H-NMR (400 MHz, CDCl_3_) (mixture of rotamers) *δ* 8.54–8.48 (m, 1H, ArH), 7.51–7.33 (m, 3H, ArH, NH), 5.62–5.43 (m, 1H, NH piperidine), 4.13–4.08 (m, 2H, NCH_2_), 4.01–3.93 (m, 1H, NCH), 2.97–2.88 (m, 2H, NCH_2_), 2.01 (d, *J* = 12.8 Hz, 2H, CCH_2_), 1.53–1.38 (m, 11H, CCH_2_, Boc); ^13^C-NMR (101 MHz, CDCl_3_) (mixture of rotamers) *δ* 170.3–169.5 (m, 1C, 1,3,5-triazine), 165.2–165.1 (m, 1C, 1,3,5-triazine), 164.1–163.6 (m, 1C, 1,3,5-triazine), 154.8–154.7 (m, 1C, Boc), 151.9 (d, *J* = 247.8 Hz, 1C, Ar, C-F), 129.8–129.6 (m, 1C, Ar), 126.0–125.6 (m, 1C, Ar), 123.4 (d, *J* = 272.9 Hz, 1C, CF_3_), 121.8 (s, 1C, Ar), 121.5 (d, *J* = 22.3 Hz, 1C, Ar, C *meta*-CF), 112.7–112.4 (m, 1C, Ar), 80.1–79.9 (m, 1C, Boc), 48.9–48.4 (m, 2C, NCH_2_), 42.8–42.0 (m, 1C, NCH), 32.0–31.6 (m, 2C, CCH_2_), 28.5 (s, 3C, Boc); MS (ESI) calcd for C_20_H_22_ClF_4_N_6_O_2_ [M − H]^−^ 489.14, found 489.16.

#### 3.2.16. *tert*-butyl 4-(4-chloro-6-((2-fluoro-4-(trifluoromethyl)phenyl)amino)-1,3,5-triazin-2-yl)piperazine-1-carboxylate (**3p**)

The above procedure was applied for intermediate **2d** (266 mg, 0.813 mmol, 1.01 equiv.), *tert*-butyl piperazine-1-carboxylate (150 mg, 0.805 mmol, 1.00 equiv.), and diisopropylethylamine (0.142 mL, 0.813 mmol, 1.01 equiv.). The product **3p** was obtained as a white solid (362 mg, 0.759 mmol, 94%) via flash column chromatography on silica gel (100–200) using hexane/ethyl acetate/triethylamine (89.9:10.0:0.1); ^1^H-NMR (400 MHz, CDCl_3_) *δ* 8.44 (t, *J* = 7.9 Hz, 1H, ArH), 7.45–7.35 (m, 3H, ArH, NH), 3.86–3.79 (m, 4H, NCH_2_), 3.53–3.51 (m, 4H, NCH_2_), 1.48 (s, 9H, Boc); ^13^C-NMR (101 MHz, CDCl_3_) *δ* 169.9 (s, 1C, 1,3,5-triazine), 164.6 (s, 1C, 1,3,5-triazine), 163.7 (s, 1C, 1,3,5-triazine), 154.6 (s, 1C, Boc), 151.9 (d, *J* = 247.8 Hz, 1C, Ar, C-F), 129.8 (d, *J* = 9.7 Hz, 1C, Ar, C *ortho*-CF), 125.8–125.4 (m 1C, Ar), 123.4 (dd, *J* = 272.9, 2.0 Hz, 1C, CF_3_), 121.8 (s, 1C, Ar), 121.4 (s, 1C, Ar), 112.6 (dd, *J* = 22.3, 3.9 Hz, 1C, Ar, C *ortho*-CF *ortho*-CCF_3_), 80.6 (s, 1C, Boc), 43.9 (s, 2C, NCH_2_), 43.7 (s, 2C, NCH_2_), 28.5 (s, 3C, Boc); MS (ESI) calcd for C_19_H_20_ClF_4_N_6_O_2_ [M − H]^−^ 475.13, found 475.07.

### 3.3. General Procedure for the Targeted Compounds 4a–4q

To a stirred solution of the corresponding chlorine-substituted targeted compound (120 mg, 1.00 equiv.) in Et_2_O:THF (10:2 mL), 10% Pd/C (30 mg, 25% *w*/*w*) and TEA (4.00 equiv.) were added. The reaction was stirred under an H_2_ atmosphere at room temperature for 48 h. After the completion of the reaction, the mixture was filtered over Celite^®^ 545, and the solvent was evaporated. The residue was purified by column chromatography to give the target compound.

#### 3.3.1. (1*R*,5*S*)-*tert*-butyl 8-(4-((4-cyano-2-fluorophenyl)amino)-1,3,5-triazin-2-yl)-3,8-diazabicyclo[3.2.1]octane-3-carboxylate (**4a**)

The above procedure was applied for product **3a** (120 mg, 0.261 mmol, 1.00 equiv.), and triethylamine (0.146 mL, 1.04 mmol, 4.00 equiv.). The product **4a** was obtained as a white solid (50.0 mg, 0.118 mmol, 45%) via flash column chromatography on silica gel (100–200) using hexane/ethyl acetate/triethylamine (49.9:50.0:0.1); ^1^H-NMR (500 MHz, CDCl_3_) *δ* 8.60 (t, *J* = 8.2 Hz, 1H, ArH), 8.30 (s, 1H, 1,3,5-triazine-H), 7.52 (s, 1H, NH), 7.45 (d, *J* = 8.8 Hz, 1H, ArH), 7.37 (dd, *J* = 10.9, 1.3 Hz, 1H, ArH, H *ortho*-CF), 4.79 (d, *J* = 20.6 Hz, 1H, NCH), 4.69 (d, *J* = 16.8 Hz, 1H, NCH), 3.95 (d, *J* = 11.1 Hz, 1H, NCH_2_), 3.80 (dd, *J* = 20.8, 12.8 Hz, 1H, NCH_2_), 3.15–3.00 (m, 2H, NCH_2_), 2.00 (d, *J* = 16.4 Hz, 2H, CCH_2_), 1.86 (d, *J* = 14.5 Hz, 2H, CCH_2_), 1.44 (s, 9H, Boc); ^13^C-NMR (126 MHz, CDCl_3_) (mixture of rotamers) *δ* 166.6 (s, 1C, 1,3,5-triazine), 163.6 (s, 1C, 1,3,5-triazine), 161.8 (s, 1C, 1,3,5-triazine), 156.0 (s, 1C, Boc), 151.3 (d, *J* = 246.7 Hz, 1C, Ar, C-F), 132.3 (d, *J* = 9.7 Hz, 1C, Ar, C *meta*-CF), 129.3 (d, *J* = 3.5 Hz, 1C, Ar), 121.2 (s, 1C, Ar), 118.5 (d, *J* = 23.1 Hz, 1C, Ar, *ortho*-CF), 118.1 (s, 1C, CN), 105.3 (d, *J* = 7.3 Hz, 1C, Ar, C-CN *meta*-CF), 80.3 (s, 1C, Boc), 53.3–52.8 (m, 2C, NCH), 49.9–48.4 (m, 2C, NCH_2_), 28.5 (s, 3C, Boc), 26.9–26.8 (m, 2C, CCH_2_); MS (ESI) calcd for C_21_H_25_FN_7_O_2_ [M + H]^+^ 426.20, found 426.10; for C_21_H_23_FN_7_O_2_ [M − H]^−^ 424.19, found 424.17.

#### 3.3.2. (1*R*,5*S*)-*tert*-butyl 3-(4-((4-cyano-2-fluorophenyl)amino)-1,3,5-triazin-2-yl)-3,8-diazabicyclo[3.2.1]octane-8-carboxylate (**4b**)

The above procedure was applied for product **3b** (120 mg, 0.261 mmol, 1.00 equiv.), and triethylamine (0.146 mL, 1.04 mmol, 4.00 equiv.). The product **4b** was obtained as white solid (30 mg, 0.071 mmol, 34%) via flash column chromatography on silica gel (100–200) using hexane/ethyl acetate/triethylamine (49.9:50.0:0.1); ^1^H-NMR (500 MHz, CDCl_3_) *δ* 8.60 (t, *J* = 8.2 Hz, 1H, ArH), 8.31 (s, 1H, 1,3,5-triazine-H), 7.51–7.46 (m, 2H, ArH, NH), 7.38 (dd, *J* = 10.7, 1.5 Hz, 1H, ArH, H *ortho*-CF), 4.51–4.31 (m, 4H, NCH, NCH_2_), 3.29–3.07 (m, 2H, NCH_2_), 1.94–1.93 (m, 2H, CCH_2_), 1.65 (d, *J* = 11.4 Hz, 2H, CCH_2_), 1.48 (s, 9H, Boc); ^13^C-NMR (126 MHz, CDCl_3_) (mixture of rotamers) *δ* 166.0 (s, 1C, 1,3,5-triazine), 165.5 (s, 1C, 1,3,5-triazine), 163.1 (s, 1C, 1,3,5-triazine), 153.6 (s, 1C, Boc), 151.3 (d, *J* = 246.7 Hz, 1C, Ar, C-F), 132.2 (d, *J* = 8.4 Hz, 1C, Ar, C *meta*-CF), 129.3 (d, *J* = 3.7 Hz, 1C, Ar), 121.1 (s, 1C, Ar), 118.5 (d, *J* = 22.9 Hz, 1C, Ar, C *ortho*-CF), 118.1 (s, 1C, CN), 105.3 (d, *J* = 9.7 Hz, 1C, Ar, C *meta*-CF), 80.3 (s, 1C, Boc), 53.7–52.7 (m, 2C, NCH), 49.5–49.0 (m, 2C, NCH_2_), 28.5 (s, 3C, Boc), 27.6–26.7 (m, 2C, CCH_2_); MS (ESI) calcd for C_21_H_23_FN_7_O_2_ [M − H]^−^ 424.19, found 424.30.

#### 3.3.3. *tert*-butyl 4-((4-((4-cyano-2-fluorophenyl)amino)-1,3,5-triazin-2-yl)amino)piperidine-1-carboxylate (**4c**)

The above procedure was applied for product **3c** (120 mg, 0.268 mmol, 1.00 equiv.), and triethylamine (0.149 mL, 1.07 mmol, 4.00 equiv.). The product **4c** was obtained as a white solid (33 mg, 0.080 mmol, 30%) via flash column chromatography on silica gel (100–200) using hexane/ethyl acetate/triethylamine (49.9:50.0:0.1); ^1^H-NMR (500 MHz, CDCl_3_) (mixture of rotamers) *δ* 8.64 (q, *J* = 8.4 Hz, 1H, ArH), 8.33–8.25 (m, 1H, 1,3,5-triazine-H), 7.64 (s, 1H, NH), 7.44–7.34 (m, 2H, ArH), 6.03–5.32 (m, 1H, NH piperidine), 4.17–3.91 (m, 3H, NCH, NCH_2_), 2.92 (t, *J* = 11.4 Hz, 2H, NCH_2_), 2.02–2.00 (m, 2H, CCH_2_), 1.48–1.39 (m, 11H, CCH_2_, Boc); ^13^C-NMR (126 MHz, CDCl_3_) (mixture of rotamers) *δ* 166.7–166.2 (m, 1C, 1,3,5-triazine), 164.6 (s, 1C, 1,3,5-triazine), 163.7–163.4 (m, 1C, 1,3,5-triazine), 154.7 (s, 1C, Boc), 151.4 (d, *J* = 246.7 Hz, 1C, Ar, C-F), 132.1 (d, *J* = 8.4 Hz, 1C, Ar, C *meta*-CF), 129.1 (d, *J* = 9.6 Hz, 1C, Ar, C *meta*-CF), 121.6–121.5 (m, 1C, Ar), 118.6–118.3 (m, 1C, Ar), 118.0–118.0 (m, 1C, CN), 105.6 (d, *J* = 8.4 Hz, 1C, C-CN *meta*-CF), 80.0–79.9 (m, 1C, Boc), 48.4 (s, 2C, NCH_2_), 42.9–42.4 (m, 1C, NCH), 32.1–31.7 (m, 2C, CCH_2_), 28.5 (s, 3C, Boc); MS (ESI) calcd for C_20_H_23_FN_7_O_2_ [M − H]^−^ 412.19, found 412.16.

#### 3.3.4. *tert*-butyl 4-(4-((4-cyano-2-fluorophenyl)amino)-1,3,5-triazin-2-yl)piperazine-1-carboxylate (**4d**)

The above procedure was applied for product **3d** (120 mg, 0.268 mmol, 1.00 equiv.), and triethylamine (0.154 mL, 1.11 mmol, 4.00 equiv.). The product **4d** was obtained as a white solid (87.0 mg, 0.218 mmol, 79%) via flash column chromatography on silica gel (100–200) using hexane/ethyl acetate/triethylamine (66.6:33.3:0.1); ^1^H-NMR (400 MHz, CDCl_3_) *δ* 8.60 (t, *J* = 8.3 Hz, 1H, ArH), 8.32 (s, 1H, 1,3,5-triazine-H), 7.52–7.45 (m, 2H, ArH, NH), 7.38 (dd, *J* = 10.4, 1.8 Hz, 1H, ArH, H *ortho*-CF), 3.86–3.79 (m, 4H, NCH_2_), 3.50 (s, 4H, NCH_2_), 1.48 (s, 9H, Boc); ^13^C-NMR (101 MHz, CDCl_3_) *δ* 166.2 (s, 1C, 1,3,5-triazine), 164.0 (s, 1C, 1,3,5-triazine), 163.4 (s, 1C, 1,3,5-triazine), 154.7 (s, 1C, Boc), 151.3 (d, *J* = 247.8 Hz, 1C, Ar, C-F), 132.2 (d, *J* = 9.7 Hz, 1C, Ar, C *meta*-CF), 129.3 (d, *J* = 3.9 Hz, 1C, Ar), 121.2 (s, 1C, Ar), 118.5 (d, *J* = 22.3 Hz, 1C, Ar, C *ortho*-CF), 118.1 (s, 1C, CN), 105.4 (d, *J* = 8.8 Hz, 1C, Ar, C-CN *meta*-CF), 80.5 (s, 1C, Boc), 43.4 (s, 2C, NCH_2_), 43.3 (s, 2C, NCH_2_), 28.5 (s, 3C, Boc); MS (ESI) calcd for C_19_H_21_FN_7_O_2_ [M − H]^−^ 398.17, found 398.00.

#### 3.3.5. (1*R*,5*S*)-*tert*-butyl 8-(4-((2-fluoro-4-(methylsulfonyl)phenyl)amino)-1,3,5-triazin-2-yl)-3,8-diazabicyclo[3.2.1]octane-3-carboxylate (**4e**)

The above procedure was applied for product **3e** (120 mg, 0.234 mmol, 1.00 equiv.), and triethylamine (0.130 mL, 0.936 mmol, 4.00 equiv.). The product **4e** was obtained as a white solid (88.0 mg, 0.184 mmol, 79%) via flash column chromatography on silica gel (100–200) using hexane/ethyl acetate/triethylamine (49.9:50.0:0.1); ^1^H-NMR (400 MHz, CDCl_3_) *δ* 8.69 (t, *J* = 8.3 Hz, 1H, ArH), 8.32 (s, 1H, 1,3,5-triazine-H), 7.73 (d, *J* = 9.2 Hz, 1H, ArH), 7.67 (dd, *J* = 10.1, 2.1 Hz, 1H, ArH, H *ortho*-CF), 7.52 (s, 1H, NH), 4.82–4.78 (m, 1H, NCH), 4.72–4.68 (m, 1H, NCH), 3.97–3.94 (m, 1H, NCH_2_), 3.85–3.77 (m, 1H, NCH_2_), 3.16–3.01 (m, 5H, NCH_2_, SO_2_CH_3_), 2.00 (t, *J* = 6.4 Hz, 2H, CCH_2_), 1.88–1.85 (m, 2H, CCH_2_), 1.45 (s, 9H, Boc); ^13^C-NMR (101 MHz, CDCl_3_) (mixture of rotamers) *δ* 166.5 (s, 1C, 1,3,5-triazine), 163.5 (s, 1C, 1,3,5-triazine), 161.7 (s, 1C, 1,3,5-triazine), 156.0 (s, 1C, Boc), 151.4 (d, *J* = 249.7 Hz, Ar, C-F), 133.9 (d, *J* = 6.8 Hz, 1C, Ar, C *meta*-CF), 132.7 (d, *J* = 9.7 Hz, 1C, Ar, C *meta*-CF), 124.3 (d, *J* = 1.9 Hz, 1C, Ar), 121.1 (s, 1C, Ar), 114.4 (d, *J* = 23.2 Hz, 1C, Ar, C *ortho*-CF), 80.3 (s, 1C, Boc), 53.3–52.8 (m, 2C, NCH), 49.9–48.4 (m, 2C, NCH_2_), 44.8 (s, 1C, SO_2_CH_3_), 28.5 (s, 3C, Boc), 26.8–26.8 (m, 2C, CCH_2_); MS (ESI) calcd for C_21_H_26_FN_6_O_4_S [M − H]^−^ 477.17, found 476.94.

#### 3.3.6. (1*R*,5*S*)-*tert*-butyl 3-(4-((2-fluoro-4-(methylsulfonyl)phenyl)amino)-1,3,5-triazin-2-yl)-3,8-diazabicyclo[3.2.1]octane-8-carboxylate (**4f**)

The above procedure was applied for product **3f** (120 mg, 0.234 mmol, 1.00 equiv.), and triethylamine (0.130 mL, 0.936 mmol, 4.00 equiv.). The product **4f** was obtained as a white solid (83 mg, 0.174 mmol, 74%) via flash column chromatography on silica gel (100–200) using hexane/ethyl acetate/triethylamine (39.9:60.0:0.1); ^1^H-NMR (400 MHz, CDCl_3_) *δ* 8.67 (t, *J* = 7.9 Hz, 1H, ArH), 8.32 (s, 1H, 1,3,5-triazine-H), 7.74 (d, *J* = 9.2 Hz, 1H, ArH), 7.67 (dd, *J* = 10.1, 2.1 Hz, 1H, ArH, H *ortho*-CF), 7.52 (s, 1H, NH), 4.52–4.32 (m, 4H, NCH, NCH_2_), 3.22–3.08 (m, 2H, NCH_2_), 3.05 (s, 3H, SO_2_CH_3_), 1.93 (d, *J* = 6.7 Hz, 2H, CCH_2_), 1.65 (d, *J* = 9.8 Hz, 2H, CCH_2_), 1.48 (s, 9H, Boc); ^13^C-NMR (101 MHz, CDCl_3_) (mixture of rotamers) *δ* 165.9 (s, 1C, 1,3,5-triazine), 165.5 (s, 1C, 1,3,5-triazine), 163.1 (s, 1C, 1,3,5-triazine), 153.6 (s, 1C, Boc), 151.4 (d, *J* = 250.7 Hz, 1C, Ar, C-F), 133.9 (d, *J* = 6.8 Hz, 1C, Ar, C-SO_2_CH_3_), 132.6 (d, *J* = 9.7 Hz, 1C, Ar, C *meta*-CF), 124.4 (d, *J* = 3.8 Hz, 1C, Ar), 121.2 (s, 1C, Ar), 114.5 (d, *J* = 23.2, 1C, Ar, C *ortho*-CF), 80.3 (s, 1C, Boc), 53.7–52.6 (m, 2C, NCH), 49.5–48.8 (m, 2C, NCH_2_), 44.8 (s, 1C, SO_2_CH_3_), 28.5 (s, 3C, Boc), 27.6–26.6 (m, 2C, CCH_2_); MS (ESI) calcd for C_21_H_26_FN_6_O_4_S [M − H]^−^ 477.17, found 477.13.

#### 3.3.7. *tert*-butyl 4-((4-((2-fluoro-4-(methylsulfonyl)phenyl)amino)-1,3,5-triazin-2-yl)amino)piperidine-1-carboxylate (**4g**)

The above procedure was applied for product **3g** (120 mg, 0.240 mmol, 1.00 equiv.), and triethylamine (0.134 mL, 0.960 mmol, 4.00 equiv.). The product **4g** was obtained as a white solid (100 mg, 0.215 mmol, 90%) via flash column chromatography on silica gel (100–200) using hexane/ethyl acetate/triethylamine (19.9:80.0:0.1); ^1^H-NMR (400 MHz, CDCl_3_) (mixture of rotamers) *δ* 8.77–8.69 (m, 1H, H_5_), 8.35–8.26 (m, 1H, 1,3,5-triazine-H), 7.72–7.51 (m, 3H, ArH, NH), 5.92–5.33 (m, 1H, (m, 1H, NH piperidine), 4.12–3.92 (m, 3H, NCH, NCH_2_), 3.05–3.04 (m, 3H, SO_2_CH_3_), 2.97–2.91 (m, 2H, NCH_2_), 2.04–2.02 (m, 2H, CCH_2_), 1.46–1.37 (m, 11H, CCH_2_, Boc); ^13^C-NMR (101 MHz, CDCl_3_) (mixture of rotamers) *δ* 166.7–166.1 (m, 1C, 1,3,5-triazine), 164.6–164.4 (m, 1C, 1,3,5-triazine), 163.6–163.4 (m, 1C, 1,3,5-triazine), 154.8 (s, 1C, Boc), 151.4 (d, *J* = 250.7 Hz, 1C, Ar, C-F), 134.2–134.1 (m, 1C, Ar), 132.6–132.5 (m, 1C, Ar), 124.2 (s, 1C, Ar), 121.4–121.1 (m, 1C, Ar), 114.7–114.3 (m, 1C, Ar), 80.0–79.9 (m, 1C, Boc), 48.5–48.4 (m, 2C, NCH_2_), 44.8 (s, 1C, SO_2_CH_3_), 43.1–42.1 (m, 1C, NCH), 32.1–31.7 (m, 2C, CCH_2_), 28.5 (s, 3C, Boc); MS (ESI) calcd for C_20_H_26_FN_6_O_4_S [M − H]^−^ 465.17, found 465.14.

#### 3.3.8. *tert*-butyl 4-(4-((2-fluoro-4-(methylsulfonyl)phenyl)amino)-1,3,5-triazin-2-yl)piperazine-1-carboxylate (**4h**)

The above procedure was applied for product **3h** (120 mg, 0.246 mmol, 1.00 equiv.), and triethylamine (0.137 mL, 0.984 mmol, 4.00 equiv.). The product **4h** was obtained as a white solid (82 mg, 0.181 mmol, 74%) via flash column chromatography on silica gel (100–200) using hexane/ethyl acetate/triethylamine (49.9:50.0:0.1); ^1^H-NMR (400 MHz, CDCl_3_) *δ* 8.67 (t, *J* = 8.3 Hz, 1H, ArH), 8.33 (s, 1H, 1,3,5-triazine-H), 7.73 (d, *J* = 9.2 Hz, 1H, ArH), 7.67 (dd, *J* = 10.4, 1.8 Hz, 1H, ArH, H *ortho*-CF), 7.50 (s, 1H, NH), 3.86–3.80 (m, 4H, NCH_2_), 3.50 (s, 4H, NCH_2_), 3.05 (s, 3H, SO_2_CH_3_), 1.48 (s, 9H, Boc); ^13^C-NMR (101 MHz, CDCl_3_) *δ* 166.2 (s, 1C, 1,3,5-triazine), 164.0 (s, 1C, 1,3,5-triazine), 163.4 (s, 1C, 1,3,5-triazine), 154.7 (s, 1C, Boc), 151.5 (d, *J* = 249.7 Hz, 1C, Ar, C-F), 134.0 (d, *J* = 5.9 Hz, 1C, Ar, C *meta*-CF), 132.6 (d, *J* = 9.7 Hz, 1C, Ar, C *meta*-CF), 124.3 (d, *J* = 2.9 Hz, 1C, Ar), 121.2 (s, 1C, Ar), 114.5 (d, *J* = 23.2 Hz, 1C, Ar, C *ortho*-CF), 80.5 (s, 1C, Boc), 44.8 (s, 1C, SO_2_CH_3_), 43.4 (s, 2C, NCH_2_), 43.3 (s, 2C, NCH_2_), 28.5 (s, 3C, Boc); MS (ESI) calcd for C_19_H_24_FN_6_O_4_S [M − H]^−^ 451.16, found 451.11.

#### 3.3.9. (1*R*,5*S*)-*tert*-butyl 8-(4-((2-chloro-4-(trifluoromethyl)phenyl)amino)-1,3,5-triazin-2-yl)-3,8-diazabicyclo[3.2.1]octane-3-carboxylate (4i) and (1*R*,5*S*)-*tert*-butyl 8-(4-((4-(trifluoromethyl)phenyl)amino)-1,3,5-triazin-2-yl)-3,8-diazabicyclo[3.2.1]octane-3-carboxylate (**4j**)

The above procedure was applied for product **3i** (120 mg, 0.231 mmol, 1.00 equiv.), and triethylamine (0.129 mL, 0.924 mmol, 4.00 equiv.). The products **4i** and **4j** were obtained as white solids—**4i** (80.0 mg, 0.165 mmol, 71%) and **4j** (24.0 mg, 0.053 mmol, 19%)—via flash column chromatography on silica gel (100–200) using hexane/ethyl acetate/triethylamine (66.6:33.3:0.1).

Product **4i**: ^1^H-NMR (500 MHz, CDCl_3_) *δ* 8.61 (d, *J* = 8.8 Hz, 1H, ArH), 8.30 (s, 1H, 1,3,5-triazine-H), 7.64 (s, 1H, ArH), 7.61 (s, 1H, NH), 7.53 (d, *J* = 8.8 Hz, 1H, ArH), 4.82–4.70 (m, 2H, NCH), 3.96 (d, *J* = 11.4 Hz, 1H, NCH_2_), 3.84–3.77 (m, 1H, NCH_2_), 3.17–3.02 (m, 2H, NCH_2_), 2.02–1.95 (m, 2H, CCH_2_), 1.88–1.84 (m, 2H, CCH_2_), 1.45 (s, 9H, Boc); ^13^C-NMR (126 MHz, CDCl_3_) (mixture of rotamers) *δ* 166.3 (s, 1C, 1,3,5-triazine), 163.5 (s, 1C, 1,3,5-triazine), 161.7 (s, 1C, 1,3,5-triazine), 156.0 (s, 1C, Boc), 138.4 (s, 1C, Ar), 126.5 (d, *J* = 3.6 Hz, Ar), 125.4–124.9 (m, 1C, Ar, C-CF_3_), 124.6 (s, 1C, Ar), 123.5 (d, *J* = 272.2 Hz, 1C, CF_3_), 122.9 (s, 1C, Ar), 120.9 (s, 1C, Ar), 80.3 (s, 1C, Boc), 53.3–52.7 (m, 2C, NCH), 49.9–48.5 (m, 2C, NCH_2_), 28.5 (s, 3C, Boc), 26.8–26.8 (m, 2C, CCH_2_); MS (ESI) calcd for C_21_H_23_ClF_3_N_6_O_2_ [M − H]^−^ 483.15, found 483.15.

Product **4j**: ^1^H-NMR (500 MHz, CDCl_3_) *δ* 8.28 (s, 1H, 1,3,5-triazine-H), 8.00 (bs, 1H, NH), 7.68 (d, *J* = 8.4 Hz, 2H, ArH), 7.57 (d, *J* = 8.4 Hz, 2H, ArH), 4.81–4.70 (m, 2H, NCH), 3.96 (d, *J* = 11.8 Hz, 1H, NCH_2_), 3.80 (q, *J* = 12.2 Hz, 1H, NCH_2_), 3.17–3.02 (m, 2H, NCH_2_), 2.02–1.98 (m, 2H, CCH_2_), 1.88–1.84 (m, 2H, CCH_2_), 1.45 (s, 9H, Boc); ^13^C-NMR (126 MHz, CDCl_3_) (mixture of rotamers) *δ* 166.2 (s, 1C, 1,3,5-triazine), 163.6 (s, 1C, 1,3,5-triazine), 161.9 (s, 1C, 1,3,5-triazine), 156.0 (s, 1C, Boc), 141.8 (s, 1C, Ar), 126.2 (d, *J* = 2.4 Hz, 2C, Ar), 125.1–124.8 (m, 1C, Ar, C-CF_3_), 124.3 (d, *J* = 272.2 Hz, 1C, CF_3_), 119.7 (s, 2C, Ar), 80.2 (s, 1C, Boc), 53.2–52.7 (m, 2C, NCH), 49.9–48.4 (m, 2C, NCH_2_), 28.5 (s, 3C, Boc), 26.9–26.8 (m, 2C, CCH_2_); MS (ESI) calcd for C_21_H_24_F_3_N_6_O_2_ [M − H]^−^ 449.19, found 449.18.

#### 3.3.10. (1*R*,5*S*)-*tert*-butyl 3-(4-((4-(trifluoromethyl)phenyl)amino)-1,3,5-triazin-2-yl)-3,8-diazabicyclo[3.2.1]octane-8-carboxylate (**4k**)

The above procedure was applied for product **3j** (120 mg, 0.231 mmol, 1.00 equiv.), and triethylamine (0.129 mL, 0.924 mmol, 4.00 equiv.). The product **4k** was obtained as a white solid (96.0 mg, 0.213 mmol, 92%) via flash column chromatography on silica gel (100–200) using hexane/ethyl acetate/triethylamine (66.6:33.3:0.1); ^1^H-NMR (500 MHz, CDCl_3_) *δ* 8.28 (s, 1H, 1,3,5-triazine-H), 8.06 (bs, 1H, NH), 7.68 (d, *J* = 8.4 Hz, 2H, ArH), 7.58 (d, *J* = 8.8 Hz, 2H, ArH), 4.50–4.35 (m, 4H, NCH, NCH_2_), 3.29–3.08 (m, 2H, NCH_2_), 1.93 (d, *J* = 3.8 Hz, 2H, CCH_2_), 1.65 (d, *J* = 10.7 Hz, 2H, CCH_2_), 1.48 (s, 9H, Boc); ^13^C-NMR (126 MHz, CDCl_3_) (mixture of rotamers) *δ* 165.7 (s, 1C, 1,3,5-triazine), 165.6 (s, 1C, 1,3,5-triazine), 163.3 (s, 1C, 1,3,5-triazine), 153.6 (s, 1C, Boc), 141.8 (s, 1C, Ar), 126.3 (d, *J* = 3.5 Hz, 2C, Ar, C *ortho*-C-CF_3_), 125.0 (d, *J* = 32.3 Hz, 1C, Ar, C-CF_3_), 124.3 (d, *J* = 272.2 Hz, 1C, CF_3_), 119.7 (s, 2C, Ar), 80.2 (s, 1C, Boc), 53.8–52.6 (m, 2C, NCH), 49.3–48.9 (m, 2C, NCH_2_), 28.5 (s, 3C, Boc), 27.5–26.7 (m, 1C, CCH_2_); MS (ESI) calcd for C_21_H_24_F_3_N_6_O_2_ [M − H]^−^ 449.19, found 449.13.

#### 3.3.11. *tert*-butyl 4-((4-((4-(trifluoromethyl)phenyl)amino)-1,3,5-triazin-2-yl)amino)piperidine-1-carboxylate (**4l**)

The above procedure was applied for product **3k** (120 mg, 0.237 mmol, 1.00 equiv.), and triethylamine (0.132 mL, 0.948 mmol, 4.00 equiv.). The product **4l** was obtained as a white solid (71.0 mg, 0.162 mmol, 68%) via flash column chromatography on silica gel (100–200) using hexane/ethyl acetate/triethylamine (49.9:50.0:0.1); ^1^H-NMR (400 MHz, CDCl_3_) (mixture of rotamers) *δ* 8.31–8.22 (m, 1H, 1,3,5-triazine-H), 7.95 (bs, 1H, NH), 7.73–7.67 (m, 2H, ArH), 7.56 (d, *J* = 7.9 Hz, 2H, ArH), 5.98–5.26 (m, 1H, NH piperidine), 4.07–3.93 (m, 3H, NCH, NCH_2_), 2.93 (t, *J* = 11.9 Hz, 2H, NCH_2_), 2.10–1.95 (m, 2H, CCH_2_), 1.46–1.39 (m, 11H, CCH_2_, Boc); ^13^C-NMR (101 MHz, CDCl_3_) (mixture of rotamers) *δ* 166.4–165.7 (m, 1C, 1,3,5-triazine), 164.6–164.4 (m, 1C, 1,3,5-triazine), 163.6–163.4 (s, 1C, 1,3,5-triazine), 154.8 (s, 1C, Boc), 141.7–141.5 (m, 1C, Ar), 126.2 (d, *J* = 2.9 Hz, 2C, Ar, C ortho-C-CF_3_), 125.2 (d, *J* = 32.9 Hz, 1C, Ar, C-CF_3_), 124.2 (d, *J* = 272.9 Hz, 1C, CF_3_), 120.0–119.8 (m, 2C, Ar), 80.0–79.9 (m, 1C, Boc), 48.5–48.3 (m, 2C, NCH_2_), 43.0–42.3 (m, 1C, NCH), 32.2–31.7 (m, 2C, CCH_2_), 28.5 (s, 3C, Boc); MS (ESI) calcd for C_20_H_24_F_3_N_6_O_2_ [M − H]^−^ 437.19, found 437.16.

#### 3.3.12. *tert*-butyl 4-(4-((4-(trifluoromethyl)phenyl)amino)-1,3,5-triazin-2-yl)piperazine-1-carboxylate (**4m**)

The above procedure was applied for product **3l** (120 mg, 0.243 mmol, 1.00 equiv.), and triethylamine (0.135 mL, 0.972 mmol, 4.00 equiv.). The product **4m** was obtained as a white solid (87.0 mg, 0.205 mmol, 84%) via flash column chromatography on silica gel (100–200) using hexane/ethyl acetate/triethylamine (74.9:25.0:0.1); ^1^H-NMR (400 MHz, CDCl_3_) *δ* 8.29 (s, 1H, 1,3,5-triazine-H), 7.82 (bs, 1H, NH), 7.68 (d, *J* = 8.6 Hz, 2H, ArH), 7.58 (d, *J* = 8.6 Hz, 2H, ArH), 3.82 (s, 4H, NCH_2_), 3.50 (s, 4H, NCH_2_), 1.48 (s, 9H, Boc); ^13^C-NMR (101 MHz, CDCl_3_) *δ* 165.9 (s, 1C, 1,3,5-triazine), 164.0 (s, 1C, 1,3,5-triazine), 163.4 (s, 1C, 1,3,5-triazine), 154.7 (s, 1C, Boc), 141.7 (s, 1C, Ar), 126.3 (d, *J* = 3.8 Hz, 2C, Ar, C *ortho*-C-CF_3_), 125.1 (d, *J* = 32.9 Hz, 1C, Ar, C-CF_3_), 124.3 (d, *J* = 274.7 Hz, 1C, CF_3_), 119.7 (s, 2C, Ar), 80.4 (s, 1C, Boc), 43.3 (s, 2C, NCH_2_), 43.2 (s, 2C, NCH_2_), 28.5 (s, 3C, Boc); MS (ESI) calcd for C_19_H_22_F_3_N_6_O_2_ [M − H]^−^ 423.18, found 423.16.

#### 3.3.13. (1*R*,5*S*)-*tert*-butyl 8-(4-((2-fluoro-4-(trifluoromethyl)phenyl)amino)-1,3,5-triazin-2-yl)-3,8-diazabicyclo[3.2.1]octane-3-carboxylate (**4n**)

The above procedure was applied for product **3m** (120 mg, 0.239 mmol, 1.00 equiv.), and triethylamine (0.133 mL, 0.956 mmol, 4.00 equiv.). The product **4n** was obtained as a white solid (97.0 mg, 0.207 mmol, 87%) via flash column chromatography on silica gel (100–200) using hexane/ethyl acetate/triethylamine (66.6:33.3:0.1); ^1^H-NMR (500 MHz, CDCl_3_) *δ* 8.53 (t, *J* = 8.2 Hz, 1H, ArH), 8.29 (s, 1H, 1,3,5-triazine-H), 7.48 (bs, 1H, NH), 7.41 (d, *J* = 8.8 Hz, 1H, ArH), 7.35 (dd, *J* = 11.1, 1.5 Hz, 1H, ArH, H *ortho*-CF), 4.81–4.68 (m, 2H, NCH), 3.95 (d, *J* = 12.2 Hz, 1H, NCH_2_), 3.83–3.77 (m, 1H, NCH_2_), 3.16–3.01 (m, 2H, NCH_2_), 1.99–1.97 (m, 2H, CCH_2_), 1.88–1.84 (m, 2H, CCH_2_), 1.45 (s, 9H, Boc); ^13^C-NMR (126 MHz, CDCl_3_) (mixture of rotamers) *δ* 166.2 (s, 1C, 1,3,5-triazine), 163.4 (s, 1C, 1,3,5-triazine), 161.7 (s, 1C, 1,3,5-triazine), 156.0 (s, 1C, Boc), 151.8 (d, *J* = 246.5 Hz, Ar, C-F), 130.5 (d, *J* = 9.6 Hz, 1C, Ar, C *meta*-CF), 125.1–124.8 (m, 1C, Ar), 123.5 (dd, *J* = 272.2, 2.4 Hz, 1C, CF_3_), 121.7 (s, 1C, Ar), 121.3 (s, 1C, Ar), 112.50 (dd, *J* = 22.8, 4.2 Hz, 1C, Ar, C *ortho*-CF), 80.3 (s, 1C, Boc), 53.3–52.8 (m, 2C, NCH), 50.0–48.4 (m, 2C, NCH_2_), 28.5 (s, 3C, Boc), 26.8–26.8 (m, 2C, CCH_2_); MS (ESI) calcd for C_21_H_23_F_4_N_6_O_2_ [M − H]^−^ 467.18, found 467.15.

#### 3.3.14. (1*R*,5*S*)-*tert*-butyl 3-(4-((2-fluoro-4-(trifluoromethyl)phenyl)amino)-1,3,5-triazin-2-yl)-3,8-diazabicyclo[3.2.1]octane-8-carboxylate (**4o**)

The above procedure was applied for product **3n** (120 mg, 0.239 mmol, 1.00 equiv.) and triethylamine (0.133 mL, 0.956 mmol, 4.00 equiv.). The product **4o** was obtained as a white solid (109 mg, 0.233 mmol, 97%) via flash column chromatography on silica gel (100–200) using hexane/ethyl acetate/triethylamine (66.6:33.3:0.1); ^1^H-NMR (500 MHz, CDCl_3_) *δ* 8.51 (t, *J* = 8.2 Hz, 1H, ArH), 8.29 (s, 1H, 1,3,5-triazine), 7.50 (bs, 1H, NH), 7.42 (d, *J* = 8.4 Hz, 1H, ArH), 7.35 (d, *J* = 11.1 Hz, 1H, ArH, H *ortho*-CF), 4.51–4.33 (m, 4H, NCH, NCH_2_), 3.17 (s, 2H, NCH_2_), 1.94–1.92 (m, 2H, CCH_2_), 1.65 (d, *J* = 10.3 Hz, 2H, CCH_2_), 1.48 (s, 9H, Boc); ^13^C-NMR (126 MHz, CDCl_3_) (mixture of rotamers) *δ* 166.0 (s, 1C, 1,3,5-triazine), 165.6 (s, 1C, 1,3,5-triazine), 163.3 (s, 1C, 1,3,5-triazine), 153.6 (s, 1C, Boc), 151.8 (d, *J* = 245.6 Hz, 1C, Ar, C-F), 130.6 (d, *J* = 9.7 Hz, 1C, Ar, C *meta*-CF), 124.9 (dd, *J* = 33.9, 7.2 Hz, 1C, Ar, C-CF_3_ *meta*-CF), 123.5 (d, *J* = 272.2 Hz, 1C, CF_3_), 121.7 (t, *J* = 3.7 Hz, Ar), 121.3 (s, 1C, Ar), 112.5 (dd, *J* = 21.7, 3.7 Hz, Ar, C *ortho*-CF), 80.2 (s, 1C, Boc), 53.8–52.7 (m, 2C, NCH), 49.3–48.8 (m, 2C, NCH_2_), 28.5 (s, 3C, Boc), 27.5–26.6 (m, 2C, CCH_2_); MS (ESI) calcd for C_21_H_25_F_4_N_6_O_2_ [M + H]^+^ 469.20, found 468.94; for C_21_H_23_F_4_N_6_O_2_ [M − H]^−^ 467.18, found 467.06.

#### 3.3.15. *tert*-butyl 4-((4-((2-fluoro-4-(trifluoromethyl)phenyl)amino)-1,3,5-triazin-2-yl)amino)piperidine-1-carboxylate (**4p**)

The above procedure was applied for product **3o** (120 mg, 0.244 mmol, 1.00 equiv.), and triethylamine (0.136 mL, 0.976 mmol, 4.00 equiv.). The product **4p** was obtained as a white solid (81.0 mg, 0.178 mmol, 73%) via flash column chromatography on silica gel (100–200) using hexane/ethyl acetate/triethylamine (49.9:50.0:0.1); ^1^H-NMR (400 MHz, CDCl_3_) (mixture of rotamers) *δ* 8.61–8.53 (m, 1H, ArH), 8.34–8.24 (m, 1H, 1,3,5-triazine-H), 7.54–7.47 (m, 1H, NH), 7.40–7.33 (m, 2H, ArH), 5.83–5.22 (m, 1H, NH piperidine), 4.11–3.95 (m, 3H, NCH, NCH_2_), 2.97–2.91 (m, 2H, NCH_2_), 2.05–2.03 (m, 2H, CCH_2_), 1.46–1.39 (m, 11H, CCH_2_, Boc); ^13^C-NMR (101 MHz, CDCl_3_) (mixture of rotamers) *δ* 166.7–166.0 (m, 1C, 1,3,5-triazine), 164.6–164.4 (m, 1C, 1,3,5-triazine), 163.7–163.5 (m, 1C, 1,3,5-triazine), 154.8 (s, 1C, Boc), 151.8 (d, *J* = 246.8 Hz, 1C, Ar, C-F), 130.4 (d, *J* = 8.7 Hz, 1C, Ar, C *meta*-CF), 125.2 (dd, *J* = 33.9, 7.8 Hz, 1C, Ar, C-CF_3_ *meta*-CF), 123.5 (d, *J* = 275.8 Hz, 1C, CF_3_), 121.6 (d, *J* = 9.6 Hz, 1C, Ar), 121.4 (s, 1C, Ar), 112.7–112.4 (m, 1C, Ar), 80.0–79.9 (m, 1C, Boc), 48.4–48.3 (m, 2C, NCH_2_), 43.1–42.3 (m, 1C, NCH), 32.2–31.7 (m, 2C, CCH_2_), 28.5 (s, 3C, Boc); MS (ESI) calcd for C_20_H_23_F_4_N_6_O_2_ [M − H]^−^ 455.18, found 455.21.

#### 3.3.16. *tert*-butyl 4-(4-((2-fluoro-4-(trifluoromethyl)phenyl)amino)-1,3,5-triazin-2-yl)piperazine-1-carboxylate (**4q**)

The above procedure was applied for product **3p** (120 mg, 0.252 mmol, 1.00 equiv.), and triethylamine (0.140 mL, 1.01 mmol, 4.00 equiv.). The product **4q** was obtained as a white solid (98.0 mg, 0.222 mmol, 88%) via flash column chromatography on silica gel (100–200) using hexane/ethyl acetate/triethylamine (74.5:25.0:0.1); ^1^H-NMR (400 MHz, CDCl_3_) *δ* 8.53 (t, *J* = 8.3 Hz, 1H, ArH), 8.32 (s, 1H, 1,3,5-triazine-H), 7.43 (d, *J* = 8.6 Hz, 2H, ArH, NH), 7.36 (d, *J* = 11.0 Hz, 1H, ArH, H *ortho*-CF), 3.85–3.81 (m, 4H, NCH_2_), 3.50 (s, 4H, NCH_2_), 1.48 (s, 9H, Boc); ^13^C-NMR (101 MHz, CDCl_3_) *δ* 166.1 (s, 1C, 1,3,5-triazine), 164.1 (s, 1C, 1,3,5-triazine), 163.4 (s, 1C, 1,3,5-triazine), 154.7 (s, 1C, Boc), 151.8 (d, *J* = 246.8 Hz, Ar, C-F), 130.5 (d, *J* = 8.7 Hz, 1C, Ar, C *meta*-CF), 125.0 (dd, *J* = 26.2, 6.7 Hz, 1C, Ar, C-CF_3_ *meta*-CF), 123.5 (d, *J* = 256.4 Hz, 1C, CF_3_), 121.7 (t, *J* = 3.9 Hz, Ar), 121.3 (s, 1C, Ar), 112.5 (dd, *J* = 23.2, 3.8 Hz, 1C, Ar, C *ortho*-CF), 80.4 (s, 1C, Boc), 43.3 (s, 2C, NCH_2_), 28.5 (s, 3C, Boc); MS (ESI) calcd for C_19_H_21_F_4_N_6_O_2_ [M − H]^−^ 441.17, found 441.00.

### 3.4. Cell Culture

The HEK293T cells were obtained from the Korean Cell Line Bank (KCLB; Seoul, Republic of Korea). The cells were cultured in Dulbecco’s Modified Eagle’s Medium (DMEM; Welgene, Daegu, Republic of Korea) supplemented with 10% fetal bovine serum (FBS; Welgene), 100 U/mL penicillin (Welgene), and 100 µg/mL streptomycin (Welgene). The cultured cells were maintained in a 5% CO_2_ incubator at 37 °C and subcultured every 3 days.

### 3.5. Dual-Expression Stable Cell Line Modeling

The HEK293T/CRE-*luc2P* stable cell line was generated by random integration into the host cellular genome through a transient transfection method using Lipofectamine 3000 reagent (Invitrogen, Carlsbad, CA, USA) and the pGL4.29 vector (Promega, Madison, WI, USA; #E8471), which contains a cAMP response element (CRE) driving the expression of the luciferase reporter gene *luc2P*. The stable cell lines were selected in DMEM medium supplemented with Hygromycin B (100 µg/mL; Invitrogen) for 3 weeks and subsequently collected by picking single colonies derived from transfected single cells. The HEK293T/CRE-*luc2P* stable cell line was randomly re-integrated into the host cell genome using a transient transfection method with Lipofectamine 3000 reagent (Invitrogen) and the pCMV6-*hGPR119*-Myc-DDK Tag vector, which encodes the human-derived *GPR119* gene (Addgene Inc., Watertown, MA, USA; #RC216685). This process resulted in the generation of a dual-expression stable HEK293T cell line that encodes both CRE-*luc2P* and *hGPR119*. The dual-stable cell line was selected in DMEM medium supplemented with Hygromycin B (100 µg/mL; Invitrogen) and G418 (200 µg/mL; Gibco, Grand Island, NY, USA) and established by picking single colonies derived from transfected single cells after 3 weeks of selection. The stability of gene expression was evaluated using the RT-PCR method.

### 3.6. RNA Isolation and RT-PCR

The cells were gently washed with 1X phosphate-buffered saline (PBS) and then lysed using TRIzol Reagent (Qiagen, Hilden, Germany). Total RNA was isolated by adding chloroform (Sigma-Aldrich, St. Louis, MO, USA). The isolated RNA was precipitated using isopropanol (Sigma-Aldrich) and then washed with 75% ethanol. After the ethanol wash, the RNA was dissolved in 0.1% DEPC water (Sigma-Aldrich) and subsequently reverse-transcribed with the TOPscript^TM^ cDNA Synthesis kit (Enzynomics, Seoul, Republic of Korea). The synthesized cDNA was amplified using Dyne 2X PreMIX-HOT (DYNE Bio, Seongnam, Republic of Korea) and gene-specific primers. The amplification was carried out under the following conditions: initial hot start at 95 °C for 3 min, followed by 30 cycles of denaturation at 95 °C for 30 s, annealing at 58 to 60 °C for 30 s, extension at 72 °C for 45 s, and final extension at 72 °C for 10 min. The amplified PCR products were semi-quantitatively assessed by 1% agarose gel electrophoresis.

### 3.7. Luciferase-Based cAMP Assay

Briefly, 96-well plates were pre-coated with Poly-L-lysine (Sigma-Aldrich). The HEK293T/CRE-*luc2P* or HEK293T/CRE-*luc2P*/*hGPR119* cells were seeded without antibiotics, such as G418 and Hygromycin B, at a density of 5 × 10^4^ cells per well in the Poly-L-lysine-coated 96-well plates. Following overnight seeding (20 h), the cells were incubated in a dose-dependent manner with test compounds or known agonists, such as Forskolin, OEA, and AS1269573, for 6 h in a 5% CO_2_ incubator at 37 °C. After 6 h, the cells were carefully washed twice with phosphate-buffered saline (PBS) and then lysed using 100 µL of 1X passive lysis buffer (Promega) to extract protein. The extracted solution was transferred to a 96-well white-walled polystyrene microplate (Greiner Bio-One GmbH, Kremsmünster, Austria) with a 20 µL volume. Subsequently, luciferase activity was measured by adding 80 µL of D-luciferin (Promega) and analyzed with the VICTOR^3^ analyzer (PerkinElmer Inc., Foster City, CA, USA). The results were evaluated with a concentration response and performed in triplicate with independent samples.

### 3.8. WST-1 Proliferation Assay

Briefly, 96-well plates were pre-coated with Poly-L-lysine (Sigma-Aldrich). The HEK293T cells were seeded at a density of 3 × 10^4^ cells per well in the Poly-L-lysine-coated 96-well plates and cultured in DMEM supplemented with 10% fetal bovine serum (FBS) and 1% penicillin/streptomycin (P/S). Following overnight seeding, the cells were treated with compounds **3d** and **3g** at concentrations ranging from 0.1 to 50 μM in fresh DMEM containing 10% FBS and 1% P/S. After 24 h of treatment, cell viability was assessed using WST-1 reagent (Roche, Mannheim, Germany). The absorbance was measured at 450 nm and then at 690 nm, which was used as the background reference wavelength, using a VICTOR^3^ spectrophotometer (PerkinElmer Inc.). All experiments were independently repeated at least three times.

### 3.9. Molecular Docking Assay

The molecular modeling was conducted using Schrödinger Suite (2024-4), Schrödinger, LLC, New York, NY, USA. For protein and ligand preparation, the X-ray crystal structure of *GPR119* (PDB code: 7WCN) was prepared using the Protein Preparation Wizard in Maestro, version 14.2. Bond orders were assigned, hydrogen atoms were added, and protonation states of the residues at pH 7.4 were generated using Epik. All hydrogen atoms were energy-minimized with the optimized potential for liquid simulation (OPLS) 2005 force field until the average root-mean-square deviation (RMSD) for hydrogen atoms was under 0.5 Å. Ligand molecules were prepared at pH 7.4 using the LigPrep module in Schrödinger. The receptor grid was centered on the native ligand (x = [124.79], y = [119.53], z = [148.17]) with an enclosing box size of 32 Å × 32 Å × 32 Å. The rigid receptor docking protocol was run in standard precision and extra precision (XP) mode with flexible ligands and post-docking minimization. Binding interactions were visualized and analyzed using Maestro, version 14.2.

## 4. Conclusions

In this study, 1,3,5-triazine was successfully introduced as a bioisostere of the nitropyrimidine moiety. We designed and synthesized thirty-three novel derivatives with assorted piperazine or piperidine scaffolds, which have the potential to exert an agonistic effect against human *GPR119* for treating T2DM.

Biological evaluation revealed that only compounds bearing a nitrile or mesyl group at the R_1_ position exhibited agonistic activity, whereas the corresponding trifluoromethyl derivatives were completely inactive, consistent with our previous findings [20,21]. Among the active nitrile and mesyl derivatives, piperazine and piperidine derivatives induced marked increases in cAMP levels; however, bicyclic piperazine analogs were less potent than their monocyclic counterparts. Furthermore, the presence of a chloro-substituent on the triazine core enhanced *GPR119* agonistic activity. Notably, compound **3c** exhibited the highest efficacy over the concentration range of 1–10 µM, with an hEC_50_ value of 2.39 µM. In addition, compound **3c** enhanced GLP-1 secretion in a dose-dependent manner, further supporting its potential as a promising *GPR119* agonist. Further structural optimization to improve pharmacological activity and reduce cytotoxicity, as well as detailed biological evaluation of these findings, are currently underway and will be reported in due course.

## Figures and Tables

**Table 1:** Structure of synthesized 1,3,5-triazine compounds.

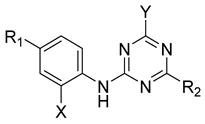							
Entry	Y = Cl			Entry	Y = H		
	R_1_	X	R_2_		R_1_	X	R_2_
**3a**	CN	F	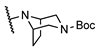	**4a**	CN	F	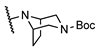
**3b**	CN	F	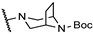	**4b**	CN	F	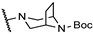
**3c**	CN	F	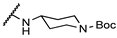	**4c**	CN	F	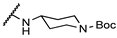
**3d**	CN	F	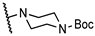	**4d**	CN	F	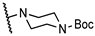
**3e**	SO_2_CH_3_	F	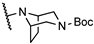	**4e**	SO_2_CH_3_	F	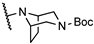
**3f**	SO_2_CH_3_	F	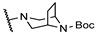	**4f**	SO_2_CH_3_	F	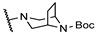
**3g**	SO_2_CH_3_	F	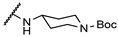	**4g**	SO_2_CH_3_	F	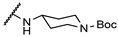
**3h**	SO_2_CH_3_	F	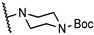	**4h**	SO_2_CH_3_	F	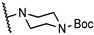
**3i**	CF_3_	Cl	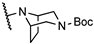	**4i**	CF_3_	Cl	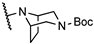
				**4j**	CF_3_	H	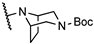
**3j**	CF_3_	Cl	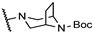	**4k**	CF_3_	H	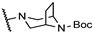
**3k**	CF_3_	Cl	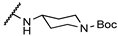	**4l**	CF_3_	H	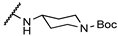
**3l**	CF_3_	Cl	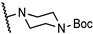	**4m**	CF_3_	H	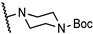
**3m**	CF_3_	F	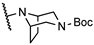	**4n**	CF_3_	F	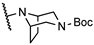
**3n**	CF_3_	F	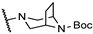	**4o**	CF_3_	F	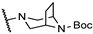
**3o**	CF_3_	F	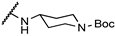	**4p**	CF_3_	F	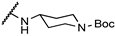
**3p**	CF_3_	F	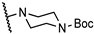	**4q**	CF_3_	F	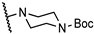

**Table 2:** Summary of the initial screening results presented in Figure 4.

Compound	CRE-*luc2P*	CRE-*luc2P*/*hGPR119*	Interpretation
DMSO	1.00 ± 0.0	1.00 ± 0.0	Negative control
Forskolin	84.0 ± 3.51	74.4 ± 7.41	Positive control
OEA	1.04 ± 0.08	1.04 ± 0.08	*GPR119* agonist control
AS1269574	1.25 ± 0.11	3.30 ± 0.33	*GPR119* agonist control
**3** **a**	0.86 ± 0.04	3.52 ± 0.53	Active
**3** **b**	0.64 ± 0.04	2.56 ± 0.66	Active
**3** **c**	1.20 ± 0.03	6.97 ± 0.68	Strong activity
**3** **d**	0.73 ± 0.04	8.23 ± 1.64	Strong activity
**3** **e**	0.62 ± 0.06	1.73 ± 0.21	Active
**3** **f**	0.36 ± 0.01	1.58 ± 0.16	Active
**3** **g**	1.53 ± 0.06	7.78 ± 0.70	Strong activity
**3** **h**	0.35 ± 0.02	1.96 ± 0.36	Active
**3** **i**	0.70 ± 0.01	1.03 ± 0.18	Inactive
**3** **j**	0.73 ± 0.05	1.03 ± 0.28	Inactive
**3** **k**	0.71 ± 0.03	0.87 ± 0.03	Inactive
**3** **d**	0.73 ± 0.04	8.23 ± 1.64	Strong activity
**3** **e**	0.62 ± 0.06	1.73 ± 0.21	Active
**3** **f**	0.36 ± 0.01	1.58 ± 0.16	Active
**3** **g**	1.53 ± 0.06	7.78 ± 0.70	Strong activity
**3** **h**	0.35 ± 0.02	1.96 ± 0.36	Active
**3** **i**	0.70 ± 0.01	1.03 ± 0.18	Inactive
**3** **l**	0.99 ± 0.09	1.00 ± 0.08	Inactive
**3m**	0.65 ± 0.05	0.97 ± 0.09	Inactive
**3n**	0.68 ± 0.04	0.94 ± 0.07	Inactive
**3o**	0.69 ± 0.09	0.94 ± 0.13	Inactive
**3p**	0.75 ± 0.12	0.86 ± 0.11	Inactive
**4a**	0.36 ± 0.02	3.46 ± 0.71	Active
**4b**	0.34 ± 0.03	3.18 ± 0.85	Active
**4c**	0.25 ± 0.02	3.12 ± 0.27	Active
**4d**	0.27 ± 0.02	1.81 ± 0.48	Active
**4e**	0.33 ± 0.02	2.18 ± 0.41	Active
**4f**	0.30 ± 0.00	2.03 ± 0.29	Active
**4g**	0.40 ± 0.02	2.00 ± 0.75	Active
**4h**	0.18 ± 0.01	1.64 ± 0.42	Active
**4i**	0.64 ± 0.10	1.10 ± 0.02	Inactive
**4j**	0.98 ± 0.14	1.04 ± 0.18	Inactive
**4k**	0.70 ± 0.09	1.22 ± 0.22	Inactive
**4l**	1.20 ± 0.14	1.40 ± 0.27	Inactive
**4m**	1.06 ± 0.12	1.23 ± 0.08	Inactive
**4n**	0.45 ± 0.03	1.12 ± 0.15	Moderate
**4o**	0.36 ± 0.03	1.21 ± 0.20	Moderate
**4p**	0.76 ± 0.09	1.10 ± 0.12	Inactive
**4q**	0.92 ± 0.11	1.18 ± 0.05	Inactive

## Data Availability

The data underlying this study are available in the published article and its Supporting Information.

## Supplementary Materials

The following supporting information can be downloaded at https://www.mdpi.com/article/10.3390/molecules31183289/s1. General information; ^1^H and ^13^C-NMR spectra of compounds; HRMS spectra of compounds **3c**, **3d**, and **3g**; Figure S1: Cytotoxicity validation of compounds **3d** and **3g**.

## Abbreviations

The following abbreviations are used in this manuscript:

*GPR119*	G protein-coupled receptor 119
GLP-1	glucagon-like peptide 1
T2DM	type 2 diabetes mellitus
