# Supplementary material for: Synthesis and Biological Evaluation of 1,3,5-Triazine Derivatives for GPR119 Agonistic Activity
Source: Molecules. 2026 Sep 16;31(18):3289. doi: 10.3390/molecules31183289 (PMC13609768; doi:10.3390/molecules31183289)
Supplement: Supplementary file 1 [file molecules-31-03289-s001.zip › molecules-4484488-supplementary.pdf]

Supporting information

Synthesis and Biological Evaluation of 1,3,5-Triazine Derivatives for *GPR119* Agonistic Activity

Bui Hoang Huu Nhan [1,†], Seong Ho Jeon [2,†], Sujin Lee [3,†], Seung Hwan Son [1], Young Duk Yang [2,*] and Seok-Ho Kim [1,*]

1 College of Pharmacy, Kangwon National University, Chuncheon 24341, Republic of Korea; 202116517@kangwon.ac.kr (B.H.H.N.); shson@kangwon.ac.kr (S.H.S.)
2 Department of Pharmacy, College of Pharmacy and Institute of Pharmaceutical Sciences, CHA University, Pocheon 11160, Republic of Korea; cmb_jsh@naver.com
3 College of Pharmacy, Daegu Catholic University, Gyungsan 38430, Republic of Korea; sujinlee20@cu.ac.kr
* Correspondence: ntsky0816@cha.ac.kr (Y.D.Y.); ksh3410@kangwon.ac.kr (S.-H.K.); Tel.: +82-31-881-7170 (Y.D.Y.); +82-33-250-6915 (S.-H.K.)
† These authors contributed equally to this work.

Table of contents

I. General information S2

II. ^{1}H and ^{13}C-NMR spectra of compounds S4

III. HRMS spectra of compounds **3c**, **3d**, and **3g** S40

IV. Cytotoxicity validation of compounds **3d** and **3g** (Supporting Information Figure 1) S42

I. General information

All chemicals were obtained from commercial suppliers, and used without further purification. All solvents used for all experiments were commercially purchased, then freshly distilled from proper dehydrating agents. All solvents used for chromatography were purchased and directly applied without further purification. ^{1}H-NMR spectra were recorded on a JEOL JNM-ECZR (500/126 MHz) and JEOL JNM-ECZ400S/L1 (400/101 MHz) spectrometers. Chemical shifts are reported in parts per million (ppm) downfield relative to tetramethylsilane as an internal standard. Peak splitting patterns are abbreviated as m (multiplet), s (singlet), bs (broad singlet), d (doublet), bd (broad doublet), t (triplet), dd (doublet of doublets), ddd (doublet of doublet of doublets), dt (doublet of triplets), td (triplet of doublets), q (quartet), and quint (quintet). ^{13}C-NMR spectra were recorded on a JEOL JNM-ECZR (500/125 MHz) and JEOL JNM-ECZ400S/L1 (400/101 MHz) spectrometers, and chemical shifts are reported in parts per million (ppm) downfield relative to tetramethylsilane as an internal standard. Mass spectra were recorded on a JEOL JMS-700 ESI mass spectrometer and a Voyager DE STR MALDI-TOF mass spectrometer. Analytical thin-layer chromatography (TLC) was performed using a commercial glass plate with silica gel 60F 254 purchased from Merck.

I. ^{1}H and ^{13}C-NMR spectra of compounds

1. ^{1}H and ^{13}C-NMR spectrum of 4-((4,6-dichloro-1,3,5-triazin-2-yl)amino)-3-fluorobenzonitrile (**2a**)

^{1}H-NMR

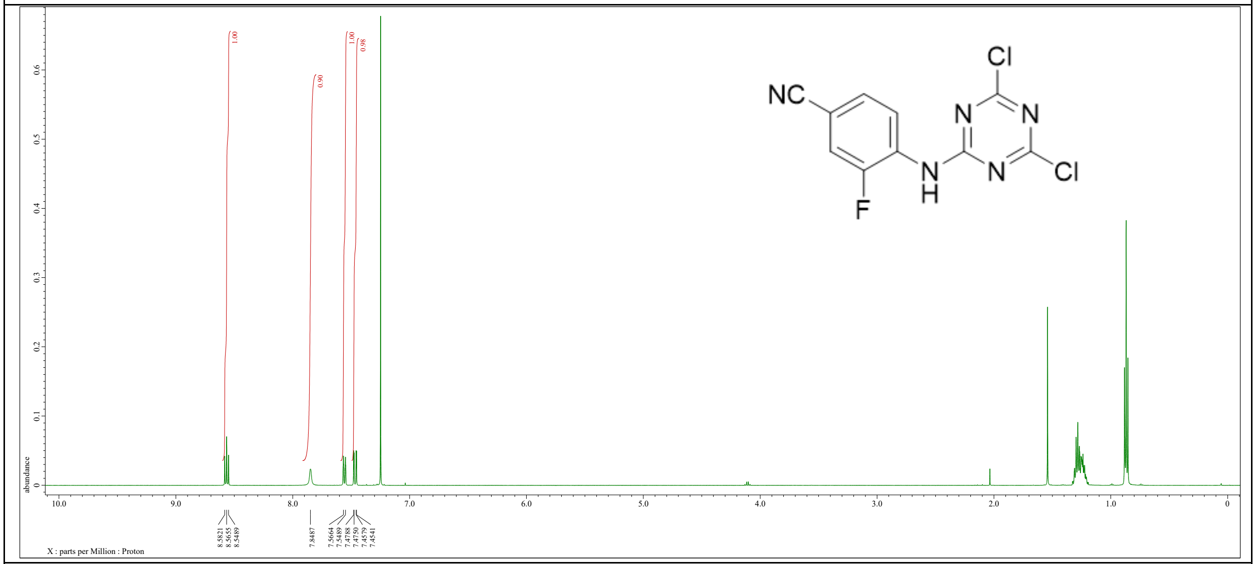

^{1}H-NMR (500 MHz, $CDCl_3$) δ 8.57 (t, J = 8.3 Hz, 1H, ArH), 7.85 (s, 1H, NH), 7.56 (d, J = 8.7 Hz, 1H, ArH), 7.47 (dd, J = 10.5, 1.9 Hz, 1H, ArH, H *ortho*-CF)

^{13}C-NMR

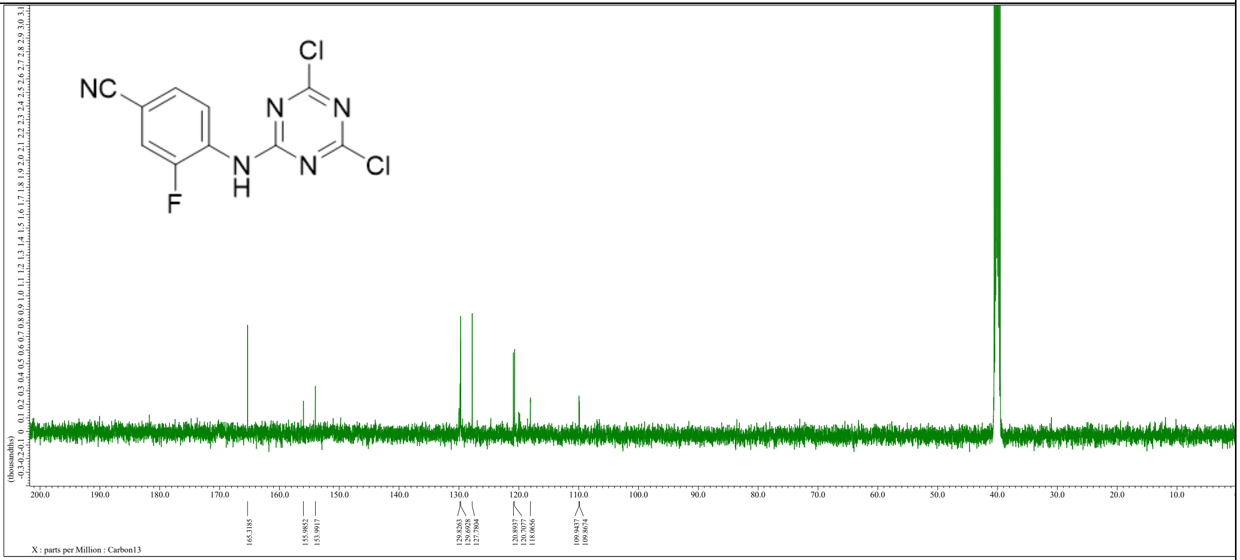

^{13}C-NMR (126 MHz, DMSO-d_6) δ 165.3 (s, 3C, 1,3,5-triazine), 155.0 (d, J = 251.2 Hz, 1C, Ar, C-F), 129.8 (d, J = 24.4 Hz, 1C, Ar, C *ortho*-CF), 129.7 (s, 1C, Ar), 127.8 (s, 1C, Ar), 120.8 (d, J = 23.4 Hz, 1C, Ar, C *ortho*-CF), 118.1 (d, J = 1.8 Hz, 1C, CN), 109.9 (d, J = 9.6 Hz, 1C, Ar, NC-C *meta*-CF)

2. ^{1}H and ^{13}C-NMR spectrum of 4,6-dichloro-N-(2-fluoro-4-(methylsulfonyl)phenyl)-1,3,5-triazin-2-amine (**2b**)

^{1}H-NMR

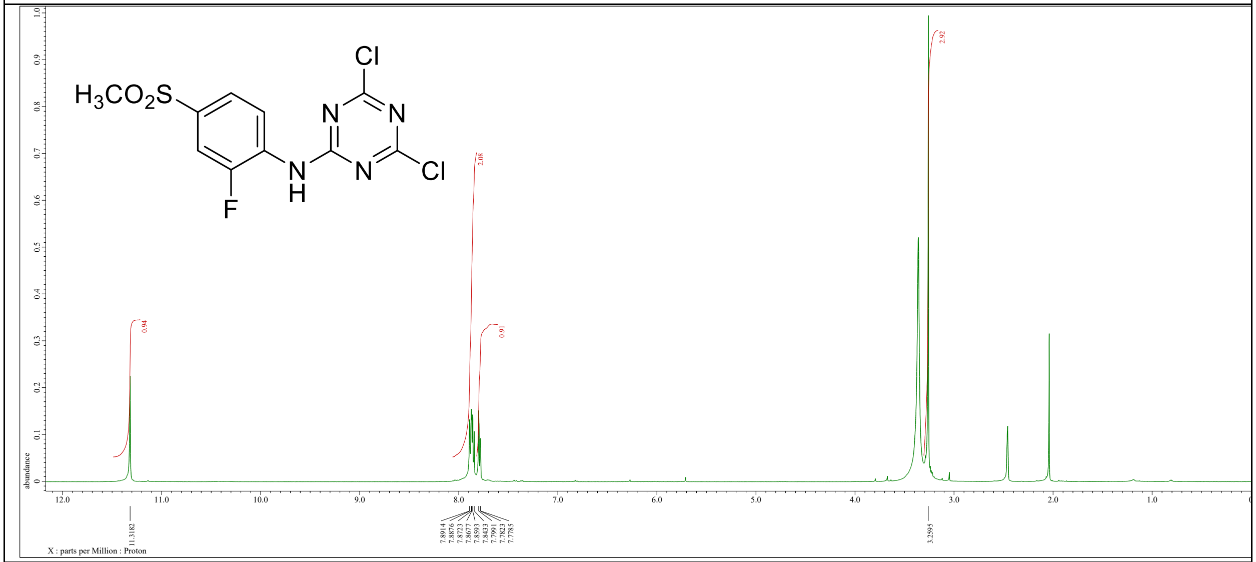

^{1}H-NMR (500 MHz, DMSO-d_6) δ 11.32 (s, 1H, NH), 7.89 – 7.84 (m, 2H, ArH), 7.79 (dd, J = 8.4, 1.9 Hz, 1H, ArH), 3.26 (s, 3H, SO_2CH_3)

^{13}C-NMR

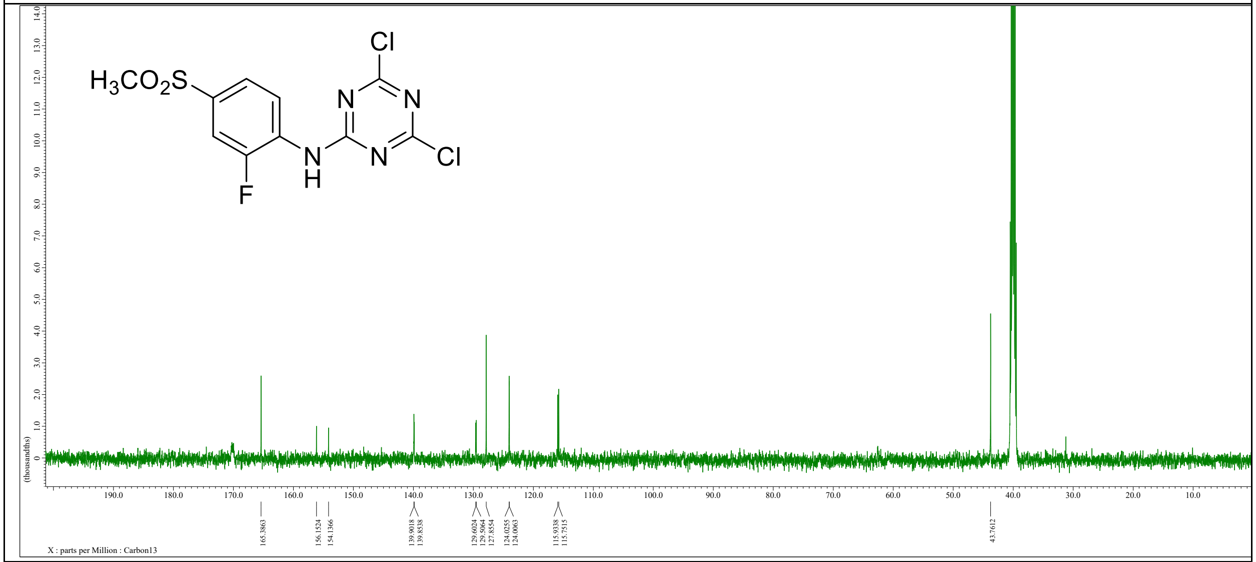

^{13}C-NMR (126 MHz, DMSO-d_6) δ 165.4 (s, 3C, 1,3,5-triazine), 155.1 (d, J = 254.0 Hz, 1C, C-F), 139.9 (d, J = 6.0 Hz, 1C, C-SO_2CH_3), 129.6 (d, J = 12.1 Hz, 1C, C *meta*-CF), 127.9 (s, 1C, Ar), 124.0 (d, J = 2.4 Hz, 1C, Ar), 115.8 (d, J = 23.0 Hz, 1C, C *ortho*-CF), 43.8 (s, 1C, SO_2CH_3)

3. ^{1}H and ^{13}C-NMR spectrum of 4,6-dichloro-N-(2-chloro-4-(trifluoromethyl)phenyl)-1,3,5-triazin-2-amine (**2c**)

^{1}H-NMR

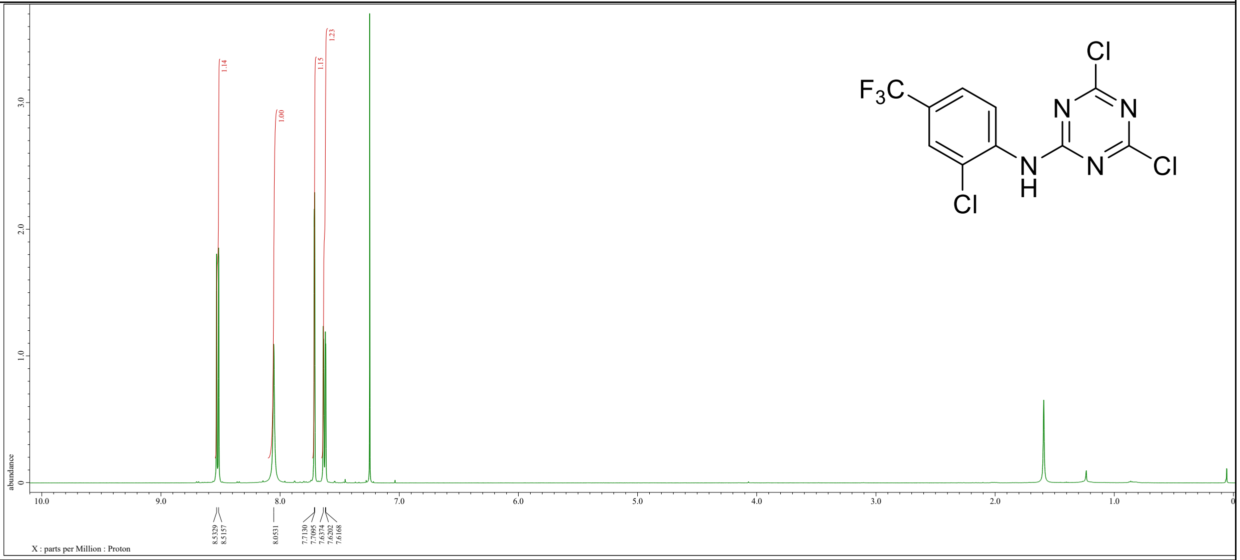

^{1}H-NMR (500 MHz, $CDCl_3$) δ 8.52 (d, J = 8.6 Hz, 1H, ArH), 8.05 (s, 1H, NH), 7.71 (d, J = 1.7 Hz, 1H, ArH), 7.63 (dd, J = 8.6, 1.7 Hz, 1H, ArH)

^{13}C-NMR

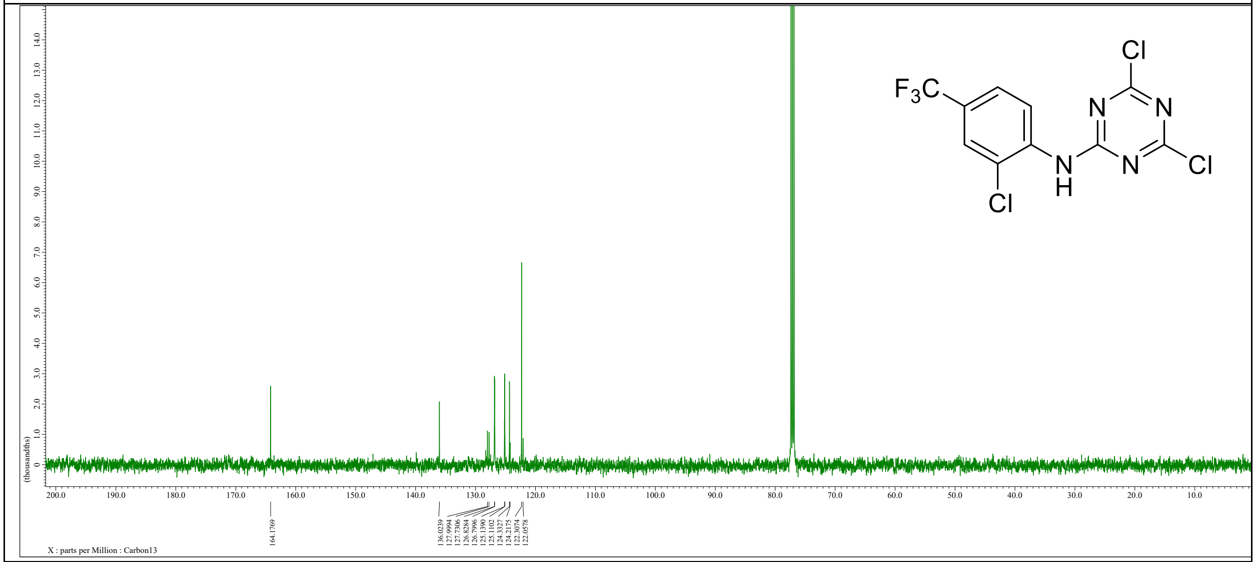

^{13}C-NMR (126 MHz, $CDCl_3$) δ 164.2 (s, 3C, 1,3,5-triazine), 136.0 (s, 1C, Ar), 127.9 (d, J = 33.9 Hz, 1C, Ar, C-CF_3), 126.8 (d, J = 3.6 Hz, 1C, Ar, C *meta*-CCF_3), 125.1 (d, J = 3.6 Hz, 1C, Ar, C *meta*-CCF_3), 124.3 (s, 1C, Ar), 123.1 (d, J = 272.1 Hz, 1C, CF_3) 122.3 (s, 1C, Ar)

4. ^{1}H and ^{13}C-NMR spectrum of 4,6-dichloro-N-(2-fluoro-4-(trifluoromethyl)phenyl)-1,3,5-triazin-2-amine (**2d**)

^{1}H-NMR

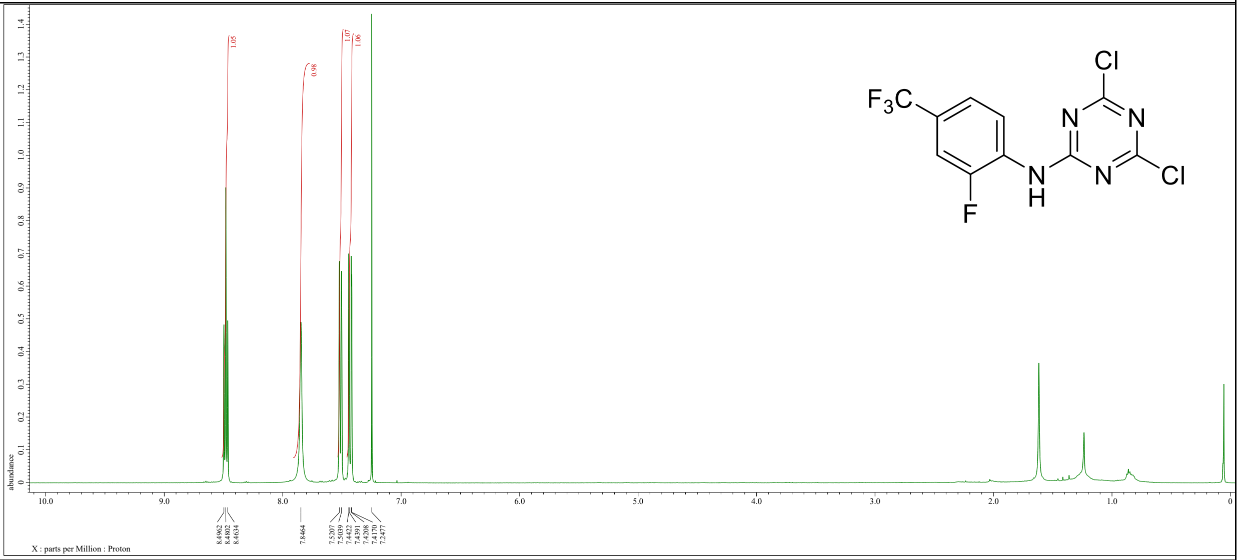

^{1}H-NMR (500 MHz, $CDCl_3$) δ 8.48 (t, J = 8.2 Hz, 1H, ArH), 7.85 (s, 1H, NH), 7.51 (d, J = 8.4 Hz, 1H, ArH), 7.43 (dd, J = 10.9, 1.7 Hz, 1H, ArH, H *ortho*-CF)

^{13}C-NMR

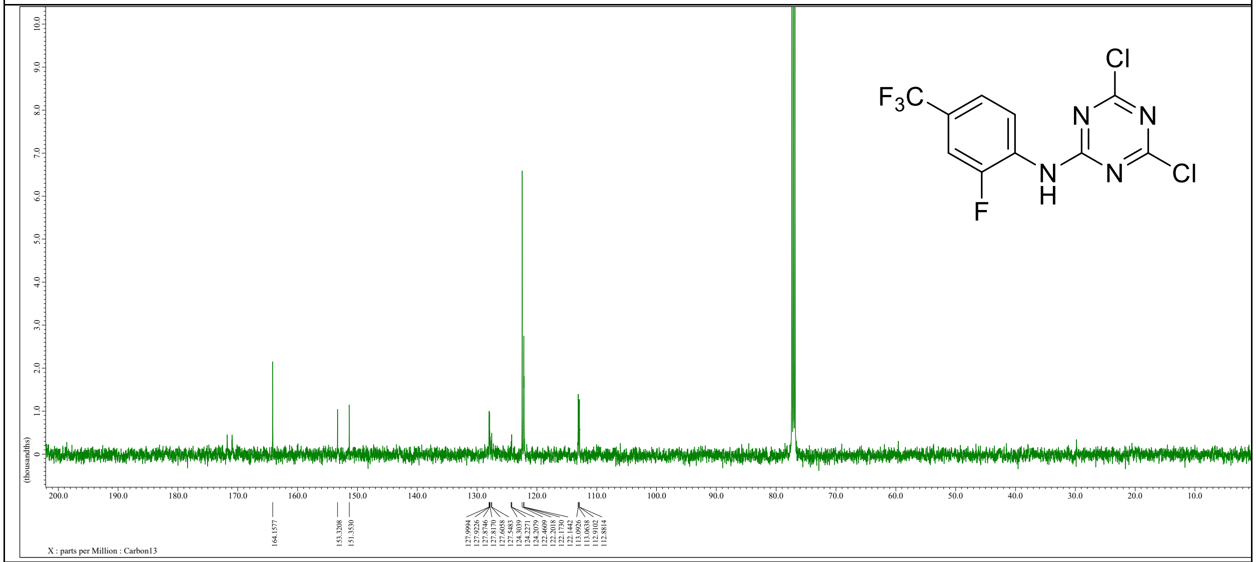

^{13}C-NMR (126 MHz, $CDCl_3$) δ 164.2 (s, 3C, 1,3,5-triazine), 152.3 (d, J = 247.9 Hz, 1C, Ar, C-F), 128.0 (d, J = 9.7 Hz, 1C, Ar, C *ortho*-CF), 127.7 (dd, J = 26.6, 7.3 Hz, 1C, C C-CF_3 *meta*-CF), 124.2 (m, 1C, CF_3), 122.5 (s, 1C, Ar), 122.2 (t, J = 3.6 Hz, 1C, Ar, C *meta*-CF), 113.0 (dd, J = 23.0, 3.6 Hz, 1C, C *ortho*-C-CF_3 *ortho*-CF)

5. ^{1}H and ^{13}C-NMR spectrum of (1*R*,5*S*)-*tert*-butyl 8-(4-chloro-6-((4-cyano-2-fluorophenyl)amino)-1,3,5-triazin-2-yl)-3,8-diazabicyclo[3.2.1]octane-3-carboxylate (**3a**)

^{1}H-NMR

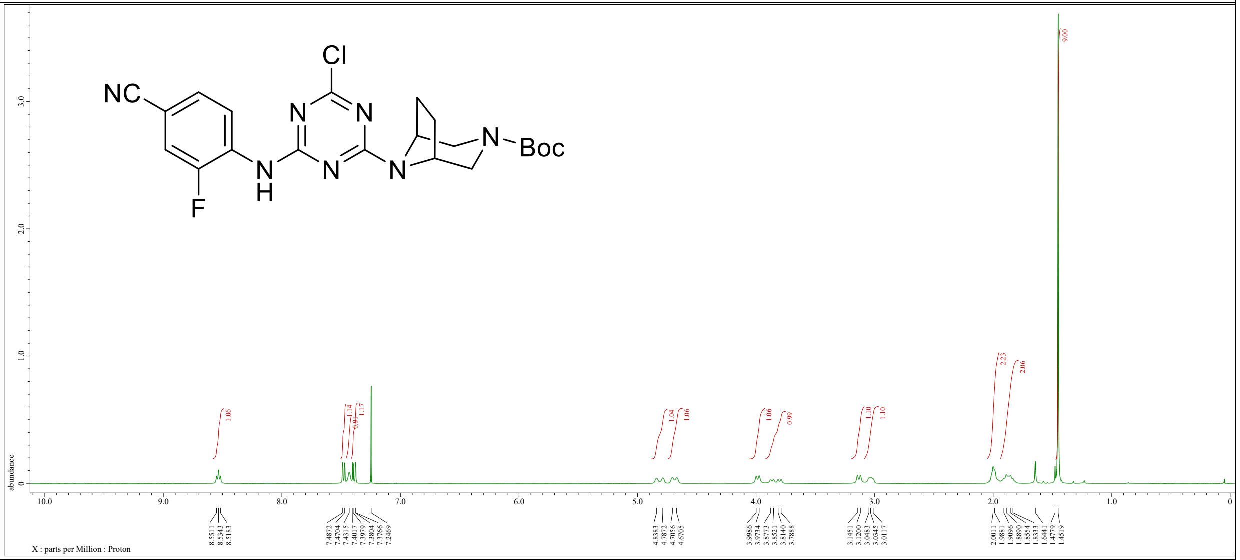

^{1}H-NMR (500 MHz, $CDCl_3$) δ 8.53 (t, J = 8.2 Hz, 1H, ArH), 7.48 (d, J = 8.4 Hz, 1H, ArH), 7.43 (s, 1H, NH), 7.39 (dd, J = 10.7, 1.9 Hz, 1H, ArH, H *ortho*-CF), 4.84 – 4.79 (m, 1H, NCH), 4.71 – 4.67 (m, 1H, NCH), 4.00 – 3.97 (m, 1H, NCH_2), 3.88 – 3.79 (m, 1H, NCH_2), 3.15 – 3.12 (m, 1H, NCH_2), 3.05 – 3.01 (m, 1H, NCH_2), 1.99 (d, J = 6.5 Hz, 2H, CCH_2), 1.91 – 1.83 (m, 2H, CCH_2), 1.45 (s, 9H, Boc)

^{13}C-NMR

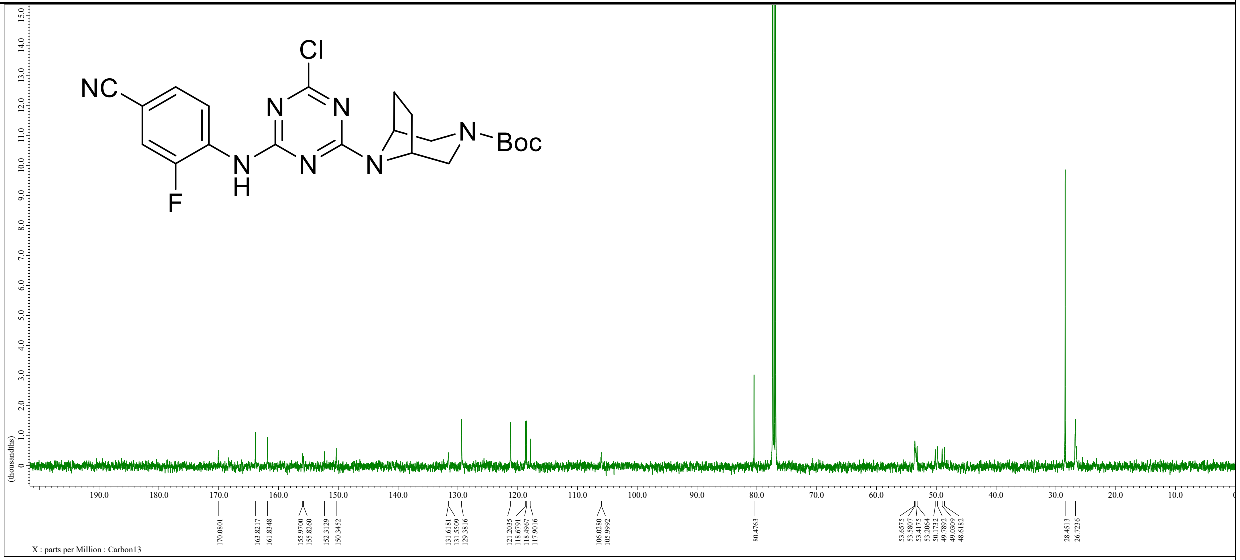

^{13}C-NMR (126 MHz, $CDCl_3$) δ 170.1 (s, 1C, 1,3,5-triazine), 163.8 (s, 1C, 1,3,5-triazine), 161.8 (s, 1C, 1,3,5-triazine), 156.0 – 155.8 (m, 1C, Boc), 151.3 (d, J = 247.9 Hz, 1C, Ar, C-F), 131.6 (d, J = 8.5 Hz, 1C, Ar, C *meta*-CF), 129.4 (s, 1C, Ar), 121.2 (s, 1C, Ar), 118.6 (d, J = 23.0 Hz, 1C, Ar, C *ortho*-CF), 117.9 (s, 1C, CN), 106.0 (d, J = 3.6 Hz, 1C, NC-C *meta*-CF), 80.5 (s, 1C, Boc), 53.7 – 53.2 (m, 2C, NCH), 50.2 – 48.6 (m, 2C, NCH_2), 28.5 (s, 3C, Boc), 26.7 (s, 2C, CCH_2)

6. ^{1}H and ^{13}C-NMR spectrum of (1*R*,5*S*)-*tert*-butyl 3-(4-chloro-6-((4-cyano-2-fluorophenyl)amino)-1,3,5-triazin-2-yl)-3,8-diazabicyclo[3.2.1]octane-8-carboxylate (**3b**)

^{1}H-NMR

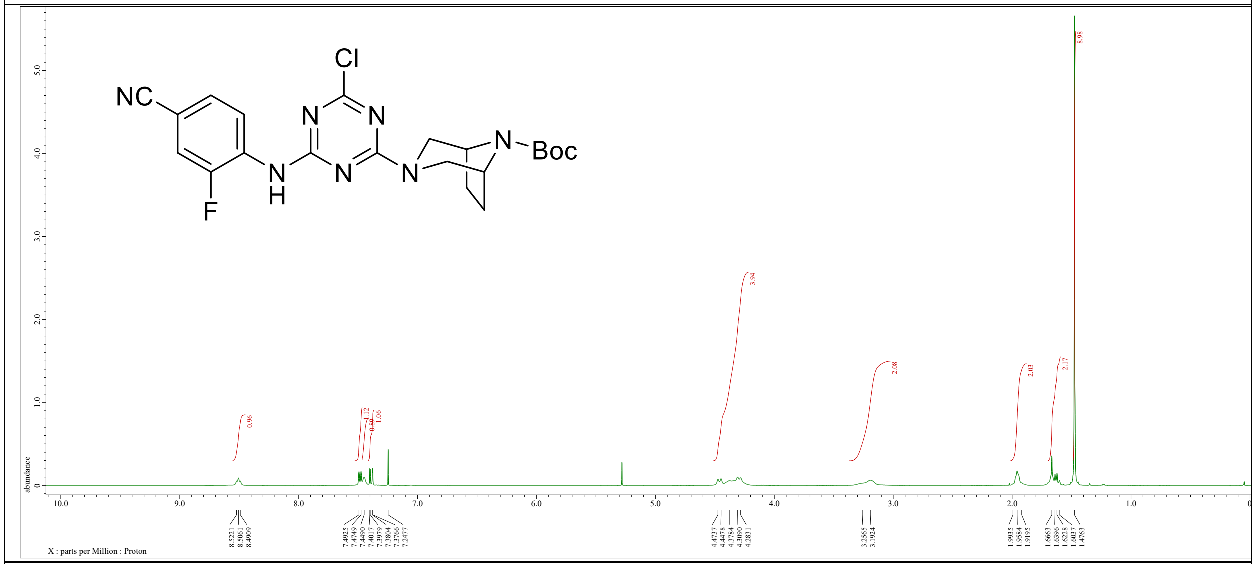

^{1}H-NMR (500 MHz, $CDCl_3$) δ 8.51 (t, J = 7.8 Hz, 1H, ArH), 7.48 (d, J = 8.8 Hz, 1H, ArH), 7.45 (s, 1H, NH), 7.39 (dd, J = 10.7, 1.9 Hz, 1H, ArH, H *ortho*-CF), 4.47 – 4.28 (m, 4H, NCH, NCH_2), 3.26 – 3.19 (m, 2H, NCH_2), 1.99 – 1.92 (m, 2H, CCH_2), 1.67 – 1.60 (m, 2H, CCH_2), 1.48 (s, 9H, Boc)

^{13}C-NMR

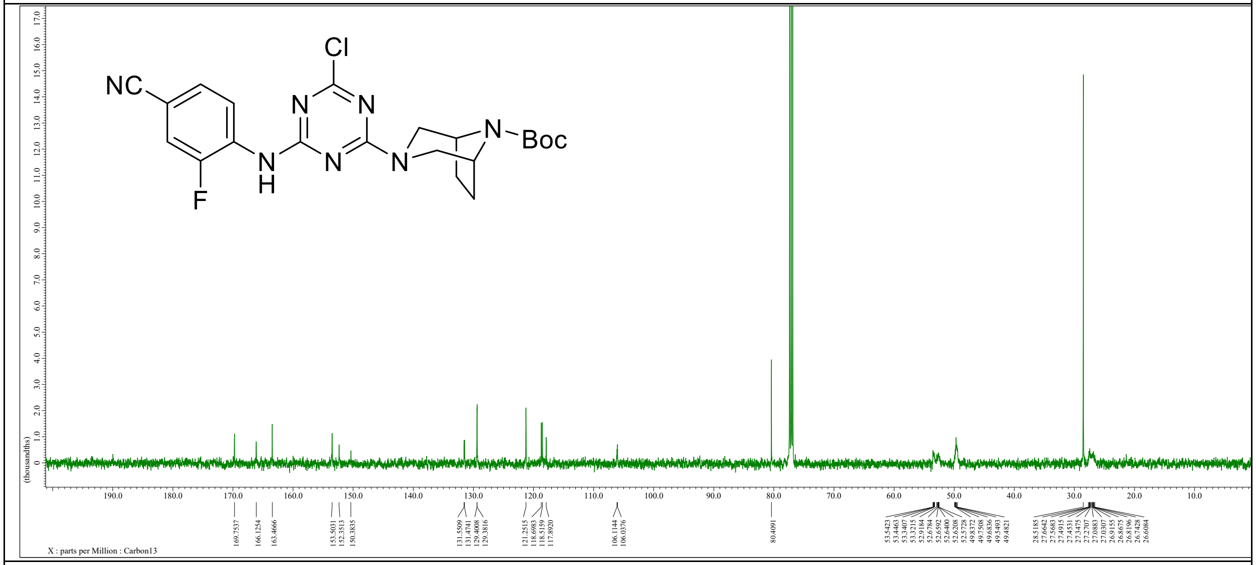

^{13}C-NMR (126 MHz, $CDCl_3$) δ 169.8 (s, 1C, 1,3,5-triazine), 166.1 (s, 1C, 1,3,5-triazine), 163.5 (s, 1C, 1,3,5-triazine), 153.5 (s, 1C, Boc), 151.4 (d, J = 247.9 Hz, 1C, Ar, C-F), 131.5 (d, J = 9.7 Hz, 1C, Ar, C *meta*-CF), 129.4 (d, J = 2.4 Hz, 1C, Ar), 121.3 (s, 1C, Ar), 118.6 (d, J = 23.0 Hz, 1C, Ar, C *ortho*-CF), 117.9 (s, 1C, CN), 106.1 (d, J = 9.7 Hz, 1C, Ar, C *meta*-CF), 80.4 (s, 1C, Boc), 53.5 – 52.5 (m, 2C, NCH), 49.9 – 49.5 (m, 2C, NCH_2), 28.5 (s, 3C, Boc), 27.7 – 26.6 (m, 2C, CCH_2)

7. ^{1}H and ^{13}C-NMR spectrum of *tert*-butyl 4-((4-chloro-6-((4-cyano-2-fluorophenyl)amino)-1,3,5-triazin-2-yl)amino)piperidine-1-carboxylate (**3c**)

^{1}H-NMR

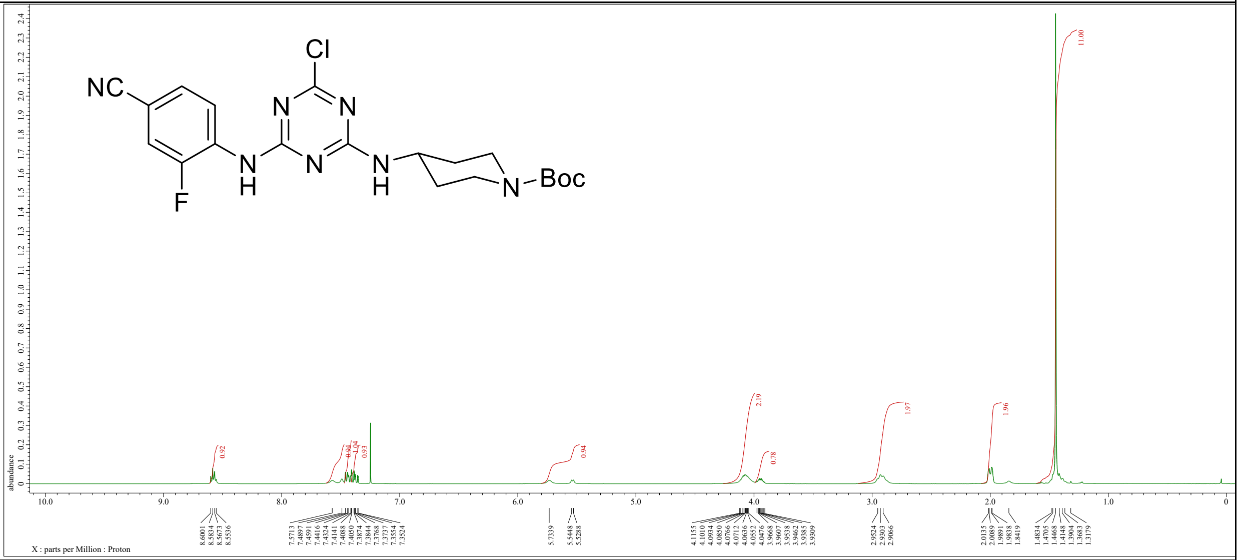

^{1}H-NMR (500 MHz, CDCl$_3$) (mixture of rotamers) δ 8.60 – 8.55 (m, 1H, ArH), 7.57 – 7.49 (m, 1H, NH), 7.46 – 7.41 (m, 1H, ArH), 7.41 – 7.35 (m, 1H, ArH), 5.73 – 5.53 (m, 1H, NH piperidine), 4.12 – 4.05 (m, 2H, NCH$_2$), 3.98 – 3.91 (m, 1H, NCH), 2.93 (t, J = 11.4 Hz, 2H, NCH$_2$), 2.00 (dd, J = 12.4, 2.5 Hz, 2H, CCH$_2$), 1.48 – 1.32 (m, 11H, CCH$_2$, Boc)

^{13}C-NMR

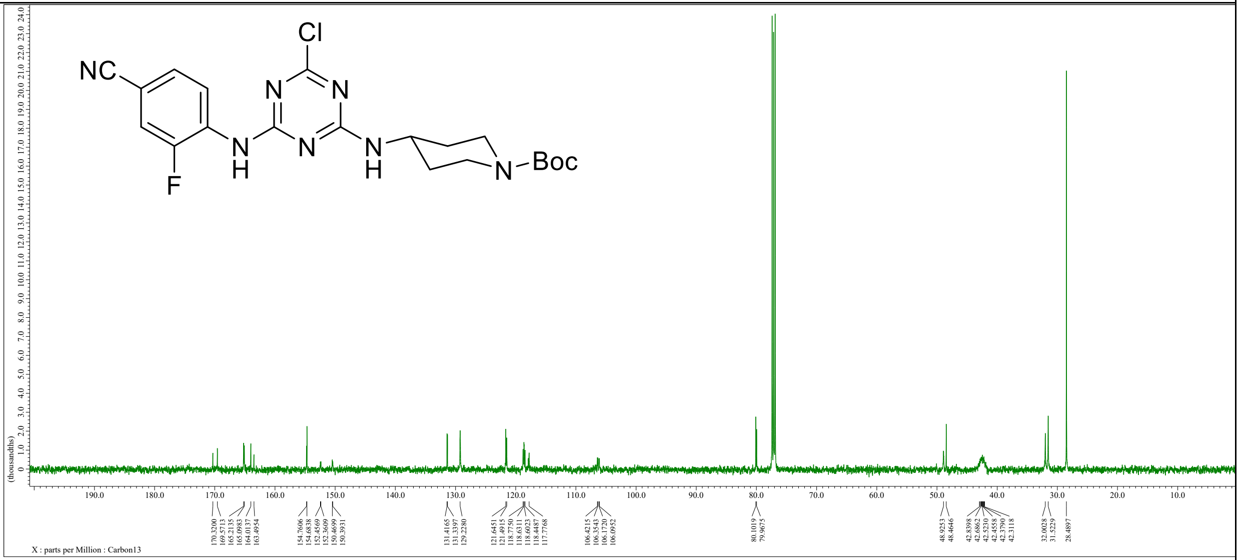

^{13}C-NMR (126 MHz, CDCl$_3$) (mixture of rotamers) δ 170.3 – 169.6 (m, 1C, 1,3,5-triazine), 165.2 – 165.1 (m, 1C, 1,3,5-triazine), 164.0 – 163.5 (m, 1C, 1,3,5-triazine), 154.8 – 154.7 (m, 1C, Boc), 151.4 (d, J = 250.4 Hz, 1C, Ar, C-F), 131.4 (d, J = 9.7 Hz, 1C, Ar, C *meta*-CF), 129.2 (s, 1C, Ar), 121.6 – 121.5 (m, 1C, Ar), 118.6 (d, J = 21.8 Hz, 1C, Ar, C *ortho*-CF), 117.8 (s, 1C, CN), 106.3 (d, J = 8.5 Hz, 1C, Ar, C *meta*-CF), 80.1 – 80.0 (m, 1C, Boc), 48.9 – 48.5 (m, 2C, NCH$_2$), 42.9 – 42.1 (m, 1C, NCH), 32.0 – 31.5 (m, 2C, CCH$_2$), 28.5 (s, 3C, Boc)

8. ^{1}H and ^{13}C-NMR spectrum of *tert*-butyl 4-(4-chloro-6-((4-cyano-2-fluorophenyl)amino)-1,3,5-triazin-2-yl)piperazine-1-carboxylate (**3d**)

**^{1}H-NMR**

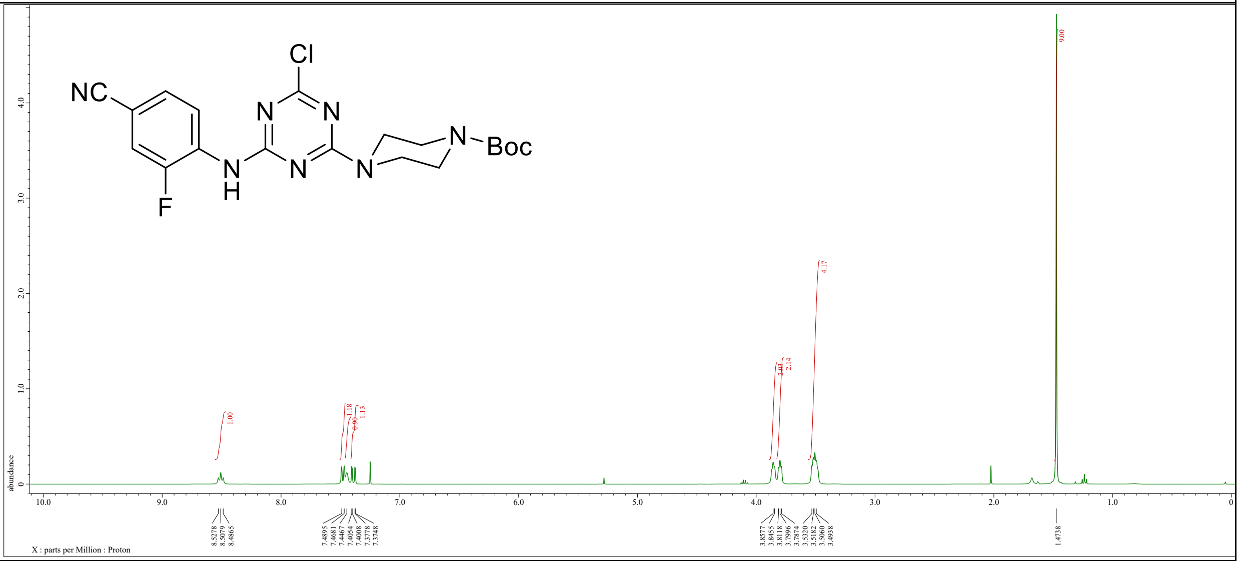

^{1}H-NMR (400 MHz, $CDCl_3$) δ 8.51 (t, J = 8.3 Hz, 1H, ArH), 7.48 (d, J = 8.6 Hz, 1H, ArH), 7.45 (s, 1H, NH), 7.39 (dd, J = 10.7, 1.5 Hz, 1H, ArH, H *ortho*-CF), 3.86 (t, J = 9.8 Hz, 2H, NCH_2), 3.80 (t, J = 9.8 Hz, 2H, NCH_2), 3.51 (quint, J = 5.1 Hz, 4H, NCH_2), 1.47 (s, 9H, Boc)

**^{13}C-NMR**

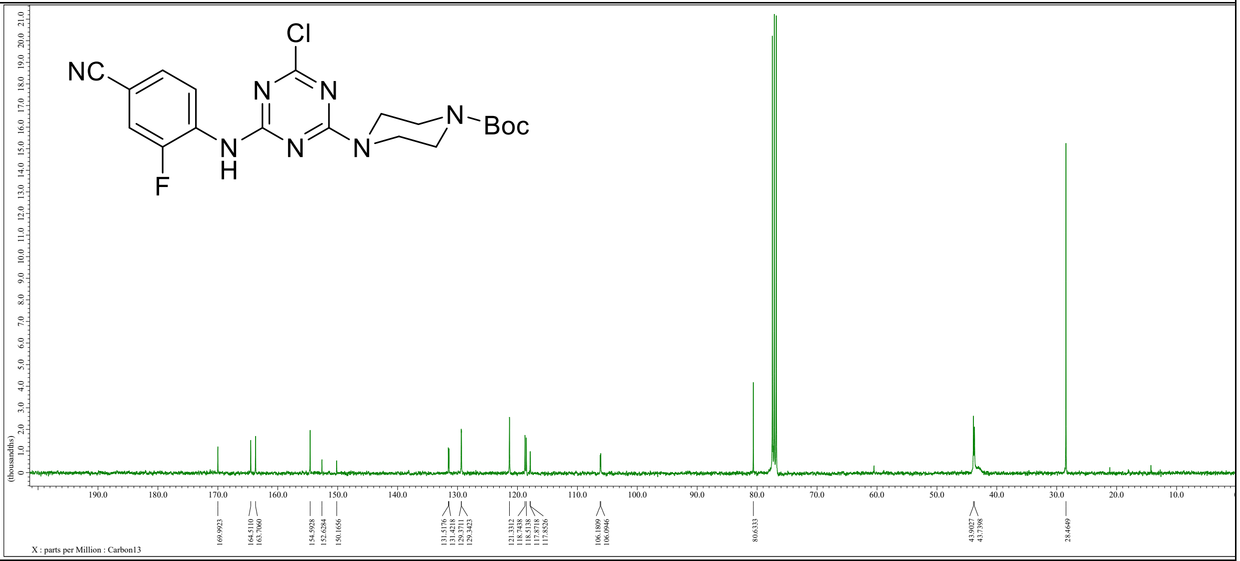

^{13}C-NMR (101 MHz, $CDCl_3$) δ 170.0 (s, 1C, 1,3,5-triazine), 164.5 (s, 1C, 1,3,5-triazine), 163.7 (s, 1C, 1,3,5-triazine), 154.6 (s, 1C, Boc), 151.4 (d, J = 248.7 Hz, Ar, C-F), 131.5 (d, J = 9.7 Hz, 1C, Ar, C *meta*-CF), 129.4 (d, J = 2.9 Hz, Ar), 121.3 (s, 1C, Ar), 118.6 (d, J = 23.3 Hz, 1C, Ar, C *ortho*-CF), 117.9 (d, J = 1.9 Hz, CN), 106.1 (d, J = 8.7 Hz, 1C, Ar), 80.6 (s, 1C, Boc), 43.9 (s, 2C, NCH_2), 43.7 (s, 2C, NCH_2), 28.5 (s, 3C, Boc)

9. ^{1}H and ^{13}C-NMR spectrum of (1*R*,5*S*)-*tert*-butyl 8-(4-chloro-6-((2-fluoro-4-(methylsulfonyl)phenyl)amino)-1,3,5-triazin-2-yl) -3,8-diazabicyclo[3.2.1]octane-3-carboxylate (**3e**)

^{1}H-NMR

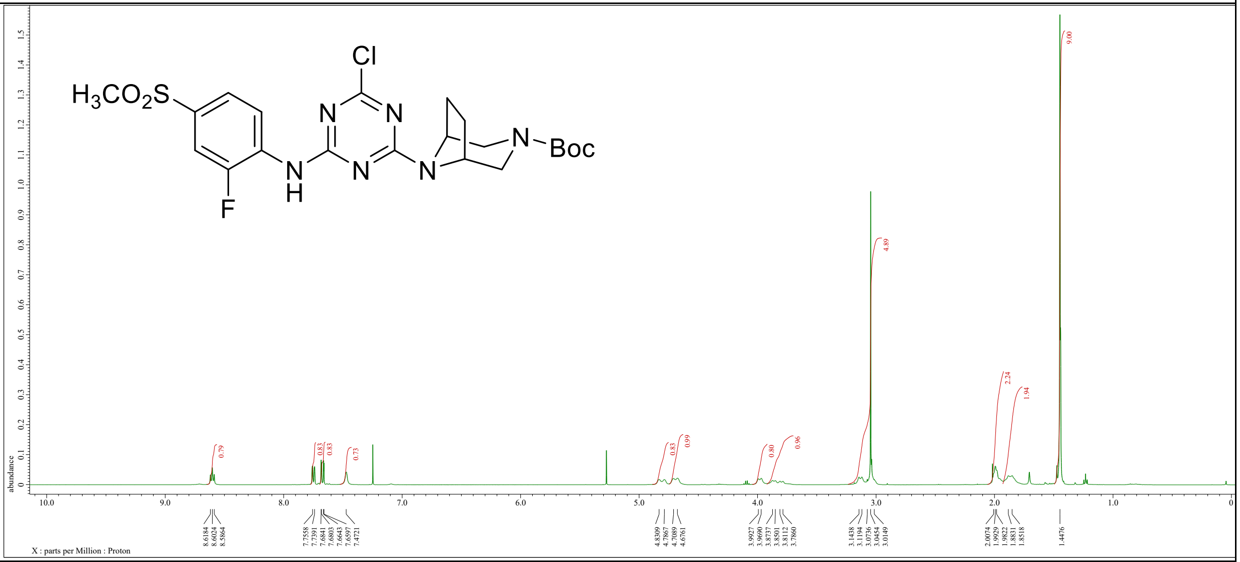

^{1}H-NMR (500 MHz, $CDCl_3$) δ 8.60 (t, *J* = 8.0 Hz, 1H, ArH), 7.75 (d, *J* = 8.4 Hz, 1H, ArH), 7.67 (dd, *J* = 10.1, 2.1 Hz, 1H, ArH, H *ortho*-CF), 7.47 (s, 1H, NH), 4.83 – 4.79 (m, 1H, NCH), 4.71 – 4.68 (m, 1H, NCH), 3.99 – 3.97 (m, 1H, NCH_2), 3.87 – 3.79 (m, 1H, NCH_2), 3.14 – 3.01 (m, 5H, NCH_2, SO_2CH_3), 2.01 – 1.98 (m, 2H, CCH_2), 1.87 (d, *J* = 15.6 Hz, 2H, CCH_2), 1.45 (s, 9H, Boc)

^{13}C-NMR

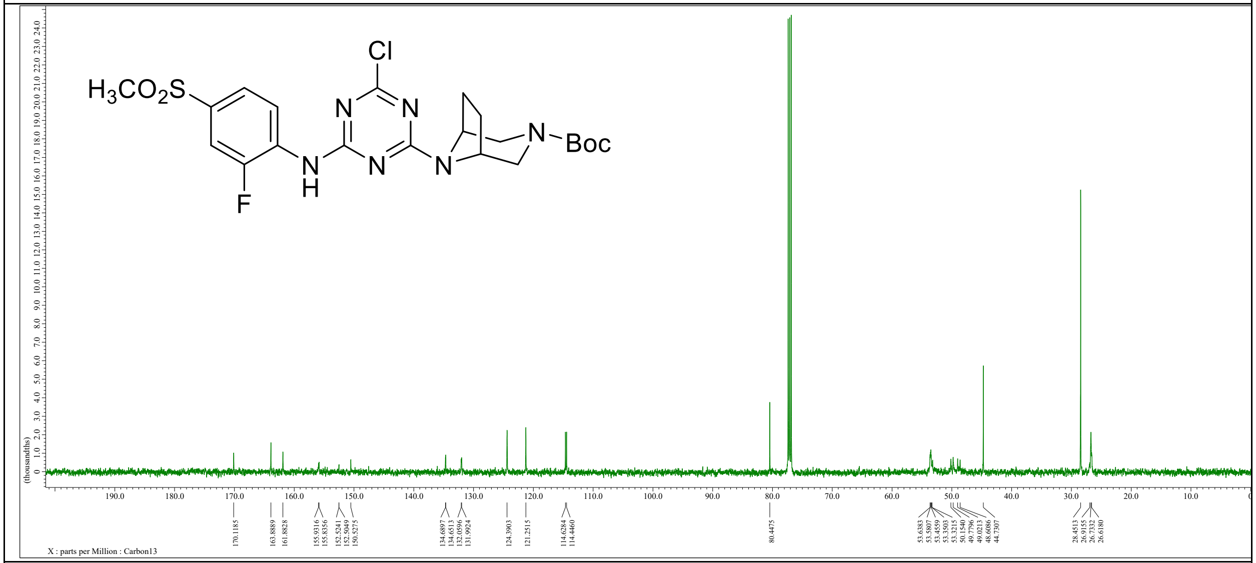

^{13}C-NMR (126 MHz, $CDCl_3$) (mixture of rotamers) δ 170.1 (s, 1C, 1,3,5-triazine), 163.9 (s, 1C, 1,3,5-triazine), 161.9 (s, 1C, 1,3,5-triazine), 155.9 – 155.8 (m, 1C, Boc), 151.5 (d, *J* = 251.6 Hz, 1C, Ar, C-F), 134.7 (d, *J* = 4.8 Hz, 1C, Ar, C-SO_2CH_3), 132.0 (d, *J* = 8.5 Hz, 1C, Ar, C *meta*-CF), 124.4 (s, 1C, Ar), 121.3 (s, 1C, Ar), 114.5 (d, *J* = 23.0 Hz, 1C, Ar, C *ortho*-CF), 80.4 (s, 1C, Boc), 53.6 – 53.3 (m, 2C, NCH), 50.2 – 48.6 (m, 2C, NCH_2), 44.7 (s, 1C, SO_2CH_3), 28.5 (s, 3C, Boc), 26.9 – 26.6 (m, 2C, CCH_2)

10. ^{1}H and ^{13}C-NMR spectrum of (1*R*,5*S*)-*tert*-butyl 3-(4-chloro-6-((2-fluoro-4-(methylsulfonyl)phenyl)amino)-1,3,5-triazin-2-yl)-3,8-diazabicyclo[3.2.1]octane-8-carboxylate (**3f**)

^{1}H-NMR

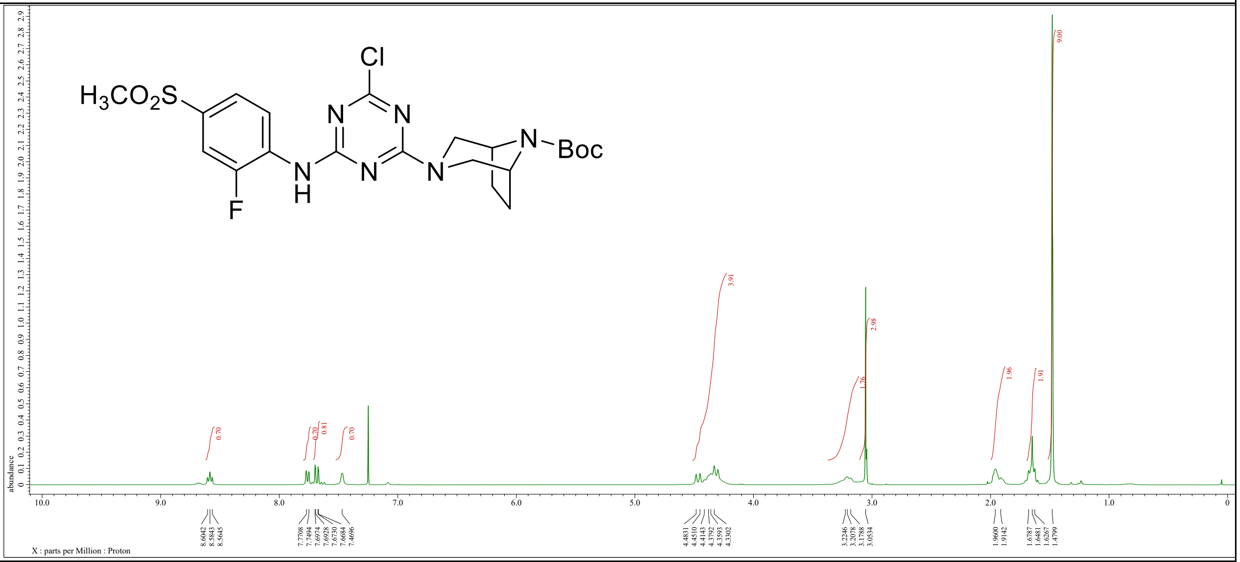

^{1}H-NMR (400 MHz, $CDCl_3$) δ 8.58 (t, J = 7.9 Hz, 1H, ArH), 7.76 (d, J = 8.6 Hz, 1H, ArH), 7.68 (dd, J = 9.8, 1.8 Hz, 1H, Ar, H *ortho*-CF), 7.47 (s, 1H, NH), 4.48 – 4.33 (m, 4H, NCH, NCH_2), 3.22 – 3.18 (m, 2H, NCH_2), 3.05 (s, 3H, SO_2CH_3), 1.96 – 1.91 (m, 2H, CCH_2), 1.68 – 1.63 (m, 2H, CCH_2), 1.48 (s, 9H, Boc)

^{13}C-NMR

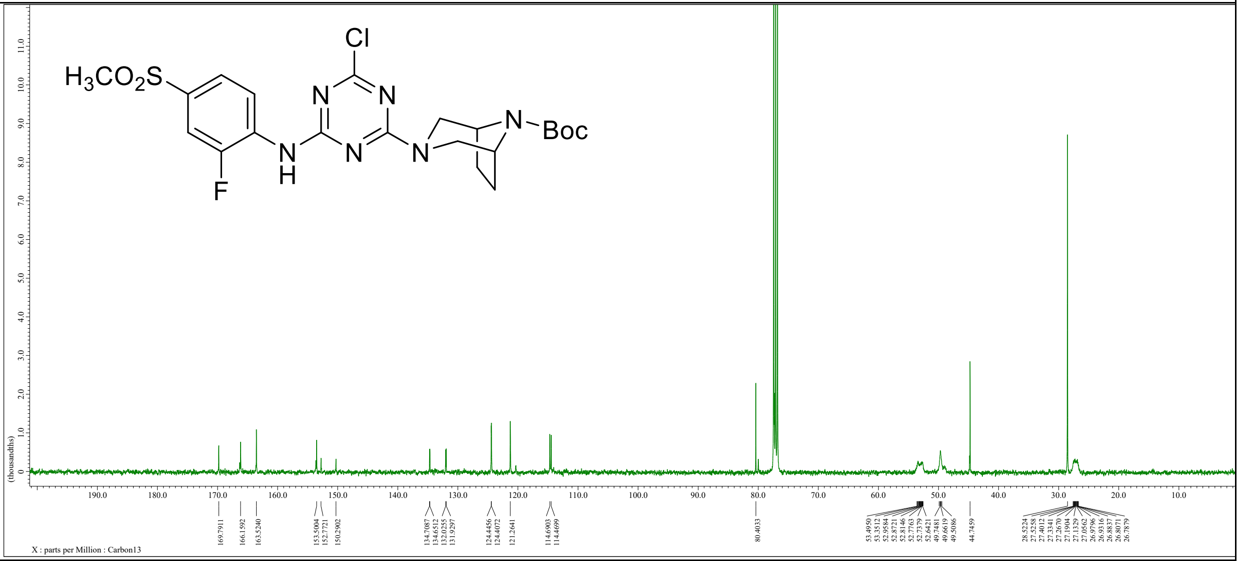

^{13}C-NMR (101 MHz, $CDCl_3$) δ 169.8 (s, 1C, 1,3,5-triazine), 166.2 (s, 1C, 1,3,5-triazine), 163.5 (s, 1C, 1,3,5-triazine), 153.5 (s, 1C, Boc), 151.5 (d, J = 250.7 Hz, 1C, Ar, C-F), 134.7 (d, J = 5.8 Hz, 1C, Ar, C-SO_2CH_3), 132.0 (d, J = 9.7 Hz, 1C, Ar, C *meta*-CF), 124.4 (d, J = 3.9 Hz, 1C, Ar), 121.3 (s, 1C, Ar), 114.6 (d, J = 22.3 Hz, 1C, Ar, C *ortho*-CF), 80.4 (s, 1C, Boc), 53.6 – 52.6 (m, 2C, NCH), 49.9 – 49.5 (m, 2C, NCH_2), 44.7 (s, 1C, SO_2CH_3), 28.5 (s, 3C, Boc), 27.6 – 26.7 (m, 2C, CCH_2)

11. ^{1}H and ^{13}C-NMR spectrum of *tert*-butyl 4-((4-chloro-6-((2-fluoro-4-(methylsulfonyl)phenyl)amino)-1,3,5-triazin-2-yl)amino)piperidine-1-carboxylate (**3g**)

^{1}H-NMR

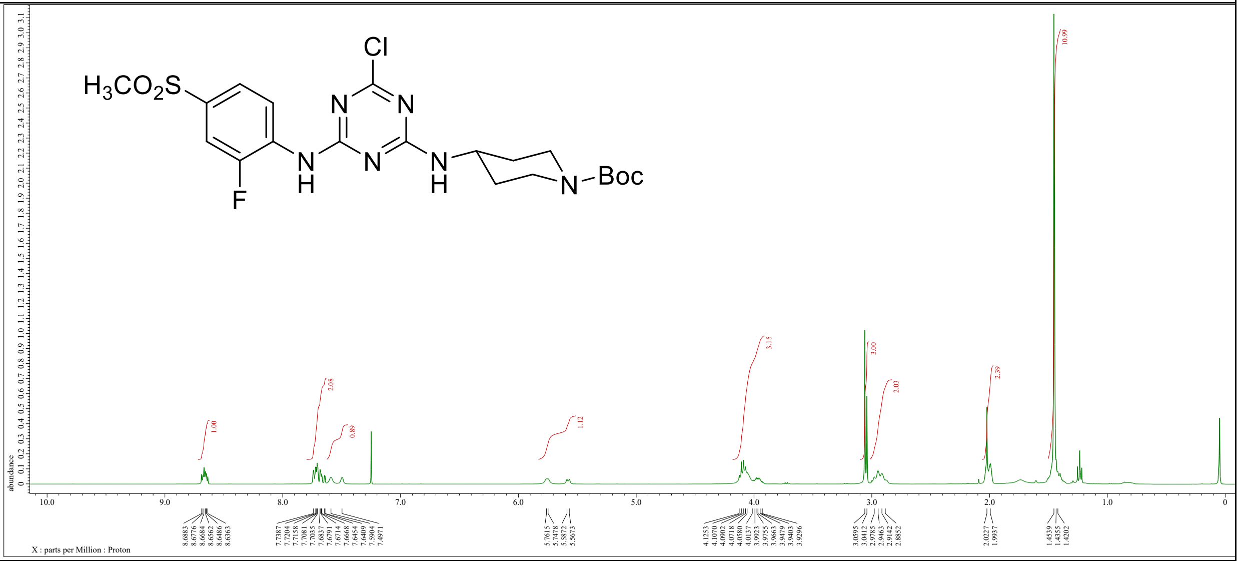

^{1}H-NMR (400 MHz, $CDCl_3$) (mixture of rotamers) δ 8.69 – 8.64 (m, 1H, ArH), 7.74 – 7.64 (m, 2H, ArH), 7.59 – 7.50 (m, 1H, NH), 5.76 – 5.57 (m, 1H, NH piperidine), 4.13 – 3.93 (m, 3H, NCH, NCH_2), 3.06 – 3.04 (m, 3H, SO_2CH_3), 2.98 – 2.89 (m, 2H, NCH_2), 2.02 – 1.99 (m, 2H, CCH_2), 1.45 – 1.42 (m, 11H, CCH_2, Boc)

^{13}C-NMR

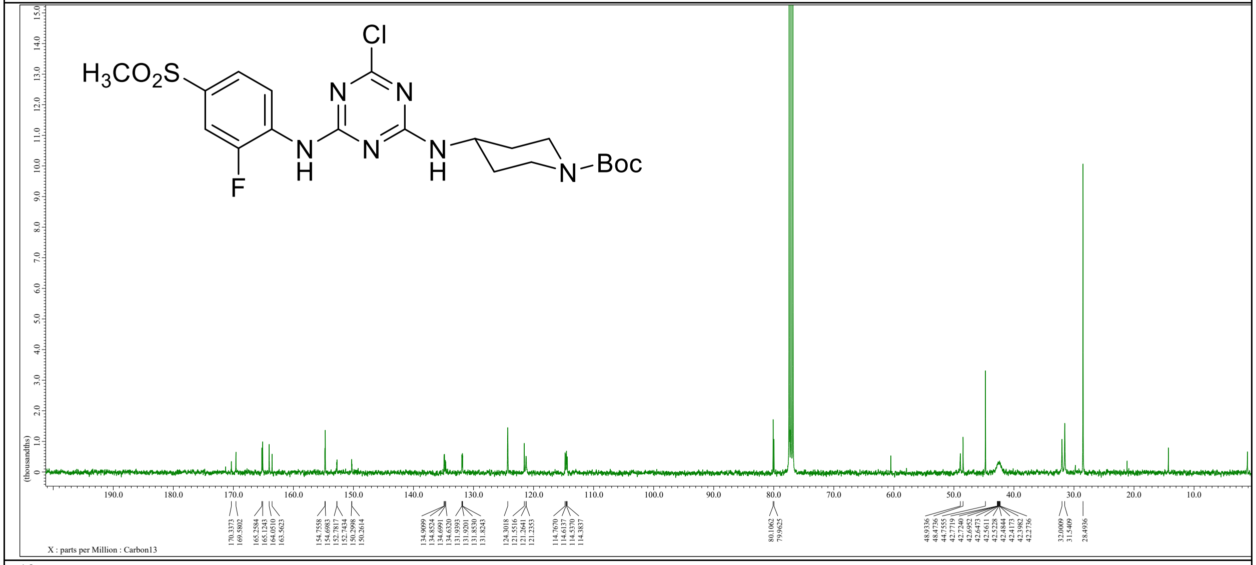

^{13}C-NMR (101 MHz, $CDCl_3$) (mixture of rotamers) δ 170.3 – 169.6 (m, 1C, 1,3,5-triazine), 165.3 – 165.1 (m, 1C, 1,3,5-triazine), 164.1 – 163.6 (m, 1C, 1,3,5-triazine), 154.8 – 154.7 (m, 1C, Boc), 151.5 (d, J = 250.7 Hz, 1C, Ar, C-F), 134.9 – 134.6 (m, 1C, Ar), 131.9 – 131.8 (m, 1C, Ar), 124.3 (s, 1C, Ar), 121.6 – 121.2 (m, 1C, Ar), 114.8 – 114.4 (m, 1C, Ar), 80.1 – 80.0 (m, 1C, Boc), 48.9 – 48.5 (m, 2C, NCH_2), 44.8 (s, 1C, SO_2CH_3), 42.8 – 42.3 (m, 1C, NCH), 32.0 – 31.5 (m, 2C, CCH_2), 28.5 (s, 3C, Boc)

12. ^{1}H and ^{13}C-NMR spectrum of *tert*-butyl 4-(4-chloro-6-((2-fluoro-4-(methylsulfonyl)phenyl)amino)-1,3,5-triazin-2-yl)piperazine-1-carboxylate (**3h**)

^{1}H-NMR

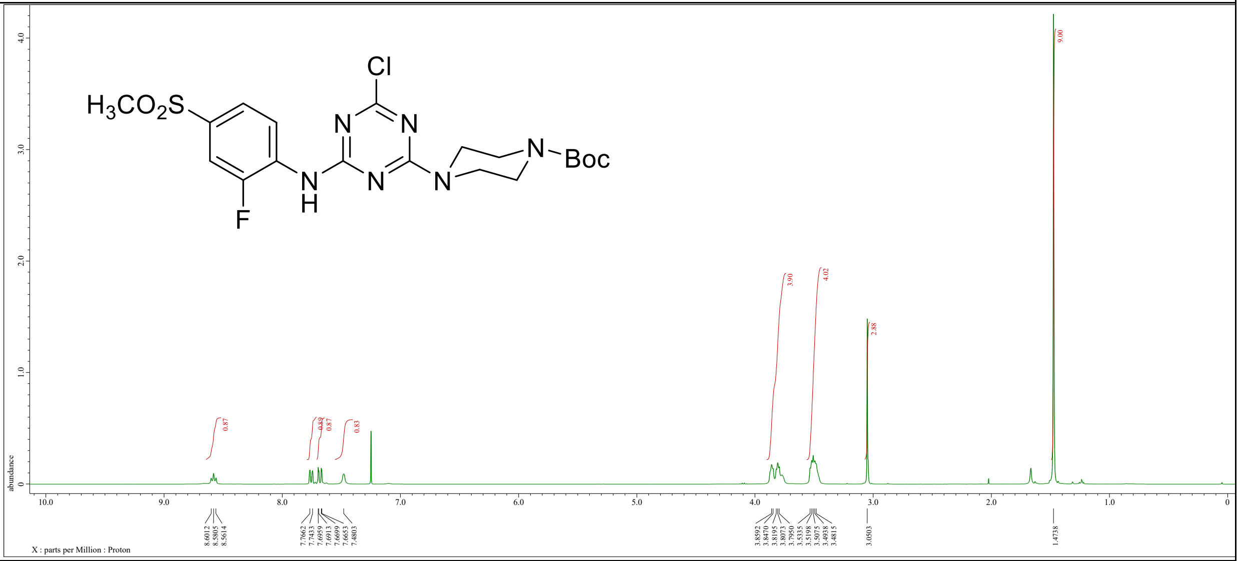

^{1}H-NMR (400 MHz, $CDCl_3$) δ 8.58 (t, J = 7.9 Hz, 1H, ArH), 7.75 (d, J = 9.2 Hz, 1H, ArH), 7.68 (dd, J = 10.4, 1.8 Hz, 1H, ArH, H *ortho*-CF), 7.48 (s, 1H, NH), 3.86 – 3.80 (m, 4H, NCH_2), 3.53 – 3.48 (m, 4H, NCH_2), 3.05 (s, 3H, SO_2CH_3), 1.47 (s, 9H, Boc)

^{13}C-NMR

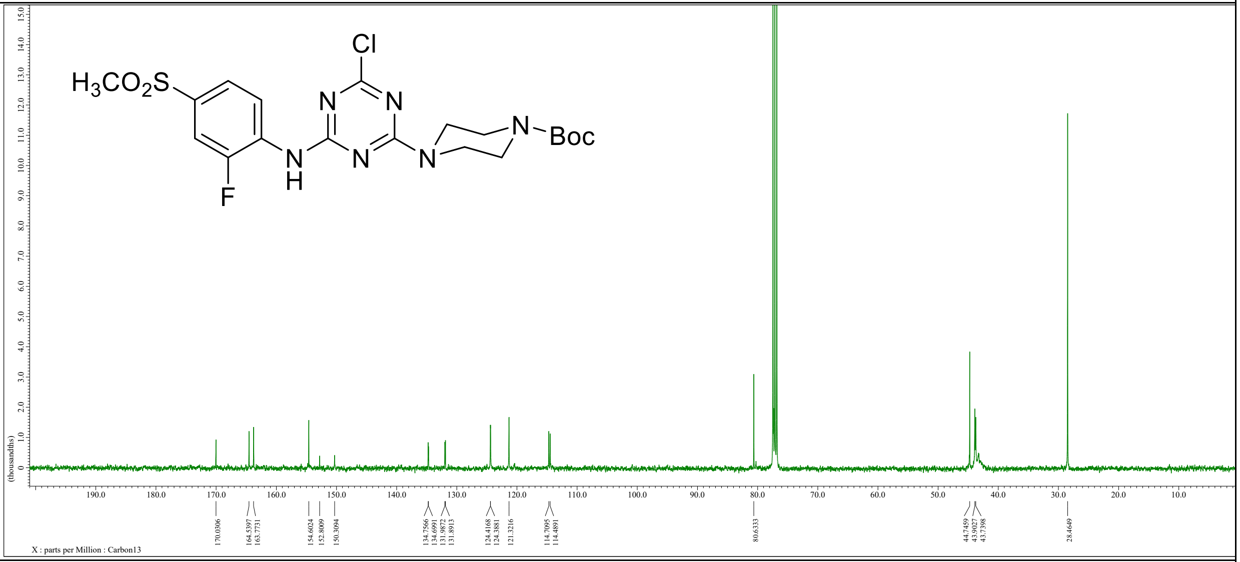

^{13}C-NMR (101 MHz, $CDCl_3$) δ 170.0 (s, 1C, 1,3,5-triazine), 164.5 (s, 1C, 1,3,5-triazine), 163.8 (s, 1C, 1,3,5-triazine), 154.6 (s, 1C, Boc), 151.6 (d, J = 251.6 Hz, 1C, Ar, C-F), 134.7 (d, J = 5.8 Hz, 1C, Ar, C-SO_2CH_3), 131.9 (d, J = 9.7 Hz, 1C, Ar, C *meta*-CF), 124.4 (d, J = 2.9 Hz, 1C, Ar), 121.3 (s, 1C, Ar), 114.6 (d, J = 22.3 Hz, 1C, Ar, C *ortho*-CF), 80.6 (s, 1C, Boc), 44.7 (s, 1C, SO_2CH_3), 43.9 (s, 2C, NCH_2), 43.7 (s, 2C, NCH_2), 28.5 (s, 3C, Boc)

13. ^{1}H and ^{13}C-NMR spectrum of (1*R*,5*S*)-*tert*-butyl 8-(4-chloro-6-((2-chloro-4-(trifluoromethyl)phenyl)amino)-1,3,5-triazin-2-yl)-3,8-diazabicyclo[3.2.1]octane-3-carboxylate (**3i**)

^{1}H-NMR

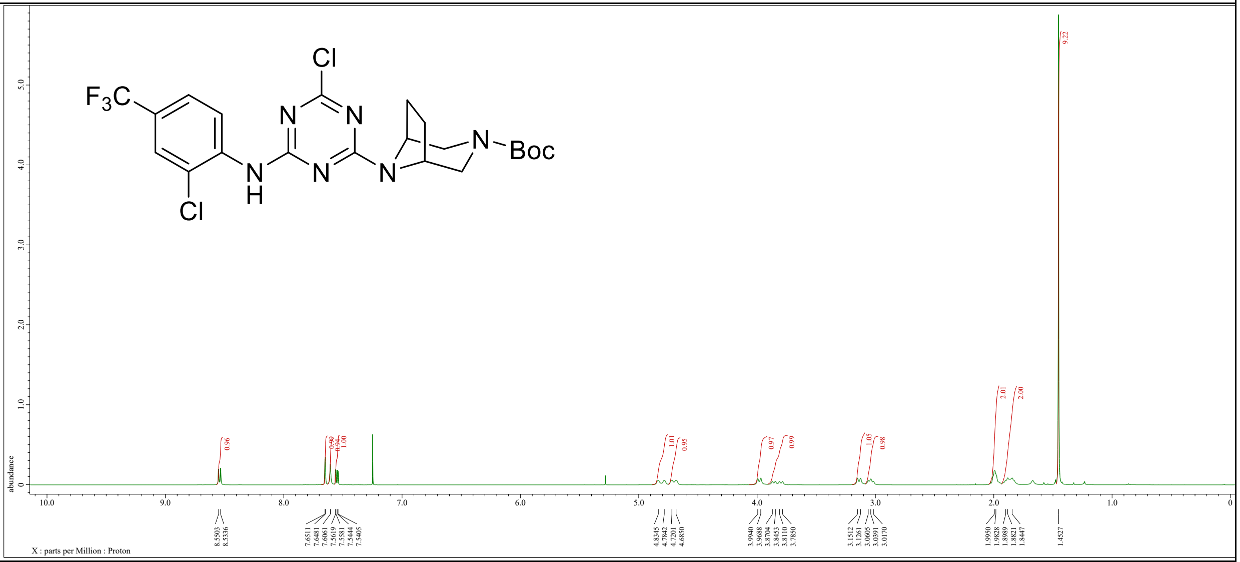

^{1}H-NMR (500 MHz, $CDCl_3$) δ 8.54 (d, J = 8.4 Hz, 1H, ArH), 7.65 (d, J = 1.5 Hz, 1H, ArH), 7.61 (s, 1H, NH), 7.55 (dd, J = 8.8, 1.9 Hz, 1H, ArH), 4.83 – 4.78 (m, 1H, NCH), 4.72 – 4.69 (m, 1H, NCH), 3.99 – 3.97 (m, 1H, NCH_2), 3.87 – 3.79 (m, 1H, NCH_2), 3.14 (d, J = 12.6 Hz, 1H, NCH_2), 3.04 (t, J = 10.9 Hz, 1H, NCH_2), 1.99 (d, J = 6.1 Hz, 2H, CCH_2), 1.90 – 1.84 (m, 2H, CCH_2), 1.45 (s, 9H, Boc)

^{13}C-NMR

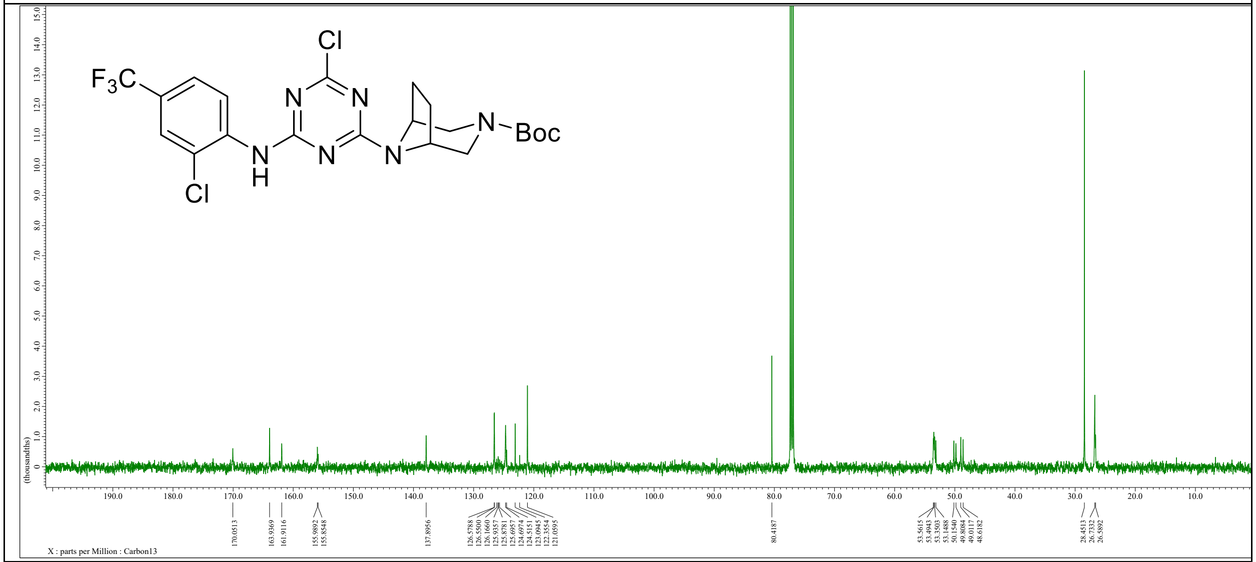

^{13}C-NMR (126 MHz, $CDCl_3$) (mixture of rotamers) δ 170.1 (s, 1C, 1,3,5-triazine), 163.9 (s, 1C, 1,3,5-triazine), 161.9 (s, 1C, 1,3,5-triazine), 156.0 – 155.9 (m, 1C, Boc), 137.9 (s, 1C, Ar), 126.6 (d, J = 3.6 Hz, Ar, C *meta*-CCF_3), 126.2 – 125.7 (m, 1C, Ar), 124.7 (s, 1C, Ar), 123.4 (d, J = 272.1 Hz, 1C, CF_3), 123.1 (s, 1C, Ar), 121.1 (s, 1C, Ar), 80.4 (s, 1C, Boc), 53.6 – 53.1 (m, 2C, NCH), 50.2 – 48.6 (m, 2C, NCH_2), 28.5 (s, 3C, Boc), 26.7 – 26.6 (m, 2C, CCH_2)

14. ^{1}H and ^{13}C-NMR spectrum of (1*R,5S*)-*tert*-butyl 3-(4-chloro-6-((2-chloro-4-(trifluoromethyl)phenyl)amino)-1,3,5-triazin-2-yl) -3,8-diazabicyclo[3.2.1]octane-8-carboxylate (**3j**)

^{1}H-NMR

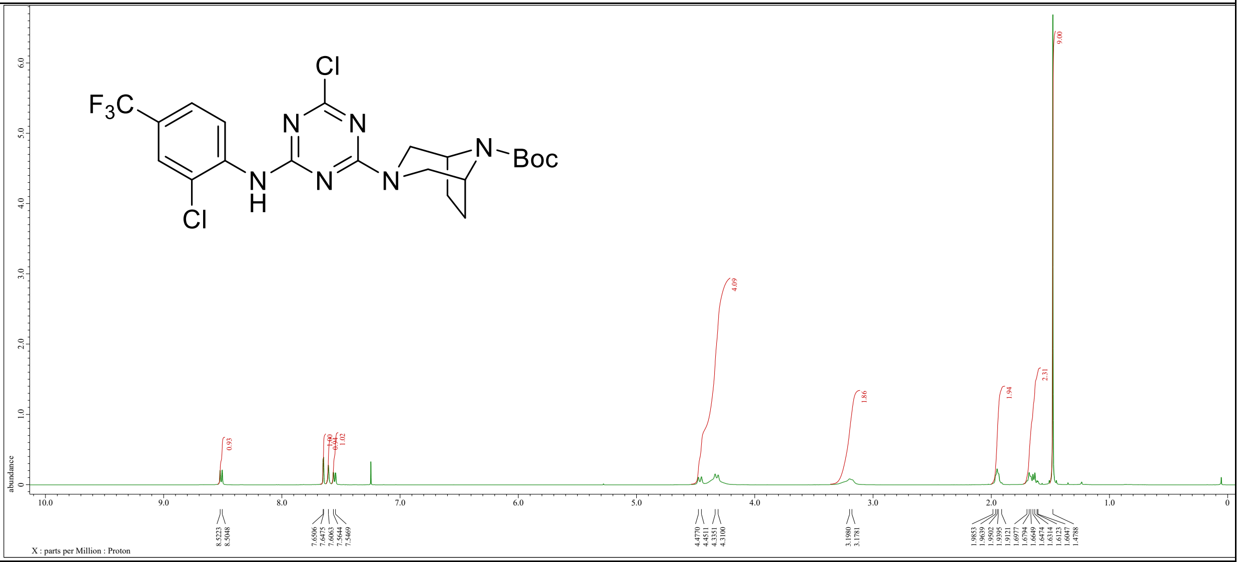

^{1}H-NMR (500 MHz, $CDCl_3$) δ 8.51 (d, J = 8.8 Hz, 1H, ArH), 7.65 (d, J = 1.5 Hz, 1H, ArH), 7.61 (s, 1H, NH), 7.56 (d, J = 8.8 Hz, 1H, ArH), 4.48 – 4.31 (m, 4H, NCH, NCH_2), 3.20 – 3.18 (m, 2H, NCH_2), 1.99 – 1.91 (m, 2H, CCH_2), 1.70 – 1.60 (m, 2H, CCH_2), 1.48 (s, 9H, Boc)

^{13}C-NMR

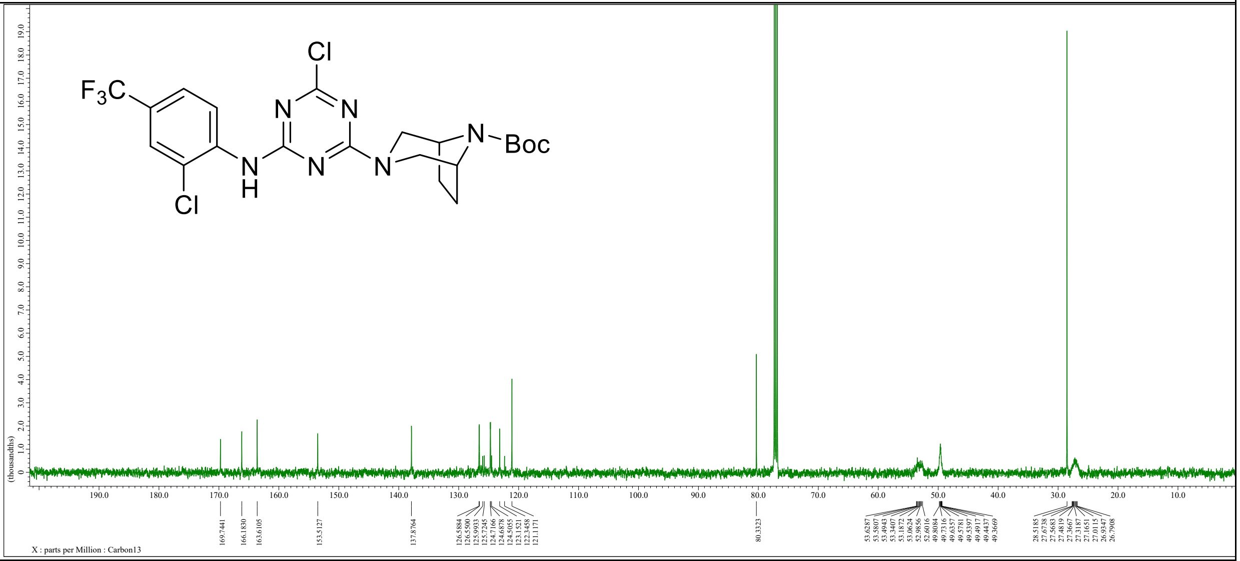

^{13}C-NMR (126 MHz, $CDCl_3$) (mixture of rotamers) δ 169.7 (s, 1C, 1,3,5-triazine), 166.2 (s, 1C, 1,3,5-triazine), 163.6 (s, 1C, 1,3,5-triazine), 153.5 (s, 1C, Boc), 137.9 (s, 1C, Ar), 126.6 (d, J = 4.8 Hz, 1C, Ar, C *meta*-CCF_3), 125.9 (d, J = 33.9 Hz, 1C, Ar, C *ortho*-CCF_3), 124.7 (d, J = 3.6 Hz, 1C, Ar, C *meta*-CCF_3), 123.4 (d, J = 272.1 Hz, 1C, CF_3), 123.2 (s, 1C, Ar), 121.1 (s, 1C, Ar), 80.3 (s, 1C, Boc), 53.6 – 52.6 (m, 2C, NCH), 49.8 – 49.4 (m, 2C, NCH_2), 28.5 (s, 3C, Boc), 27.7 – 26.8 (m, 2C, CCH_2)

15. ^{1}H and ^{13}C-NMR spectrum of *tert*-butyl 4-((4-chloro-6-((2-chloro-4-(trifluoromethyl)phenyl)amino)-1,3,5-triazin-2-yl)amino)piperidine-1-carboxylate (**3k**)

^{1}H-NMR

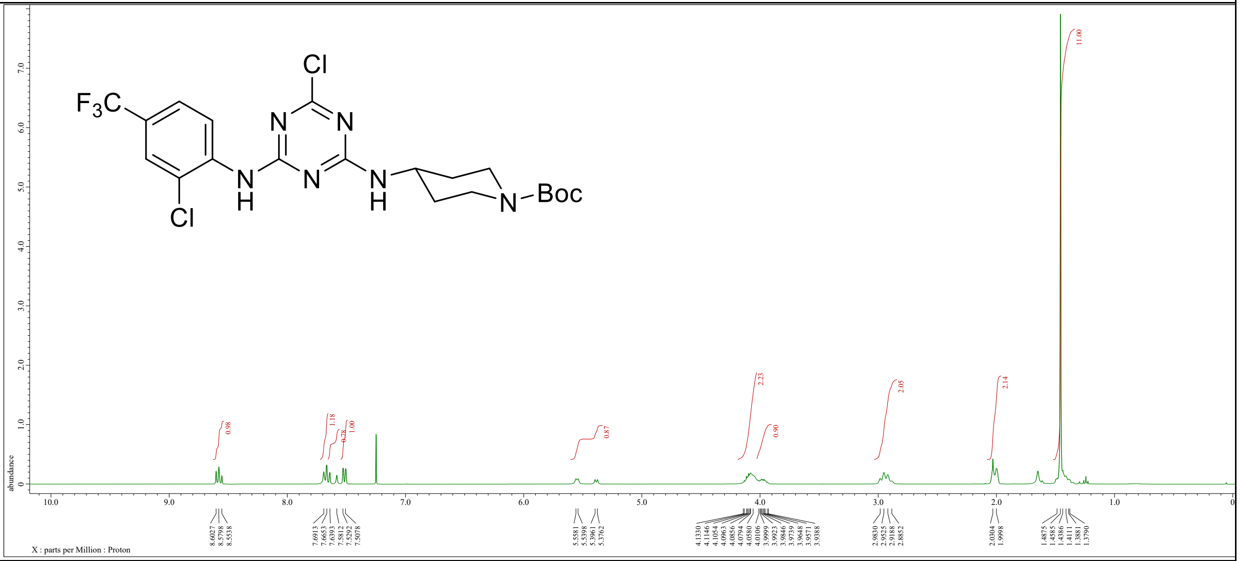

^{1}H-NMR (400 MHz, $CDCl_3$) (mixture of rotamers) δ 8.58 (t, J = 9.8 Hz, 1H, ArH), 7.68 (d, J = 10.4 Hz, 1H, ArH), 7.64 – 7.58 (m, 1H, NH), 7.52 (d, J = 8.6 Hz, 1H, ArH), 5.56 – 5.38 (m, 1H, NH piperidine), 4.14 – 4.06 (m, 2H, NCH_2), 4.01 – 3.93 (m, 1H, NCH), 2.98 – 2.89 (m, 2H, NCH_2), 2.03 – 2.00 (m, 2H, CCH_2), 1.49 – 1.38 (m, 11H, CCH_2, Boc)

^{13}C-NMR

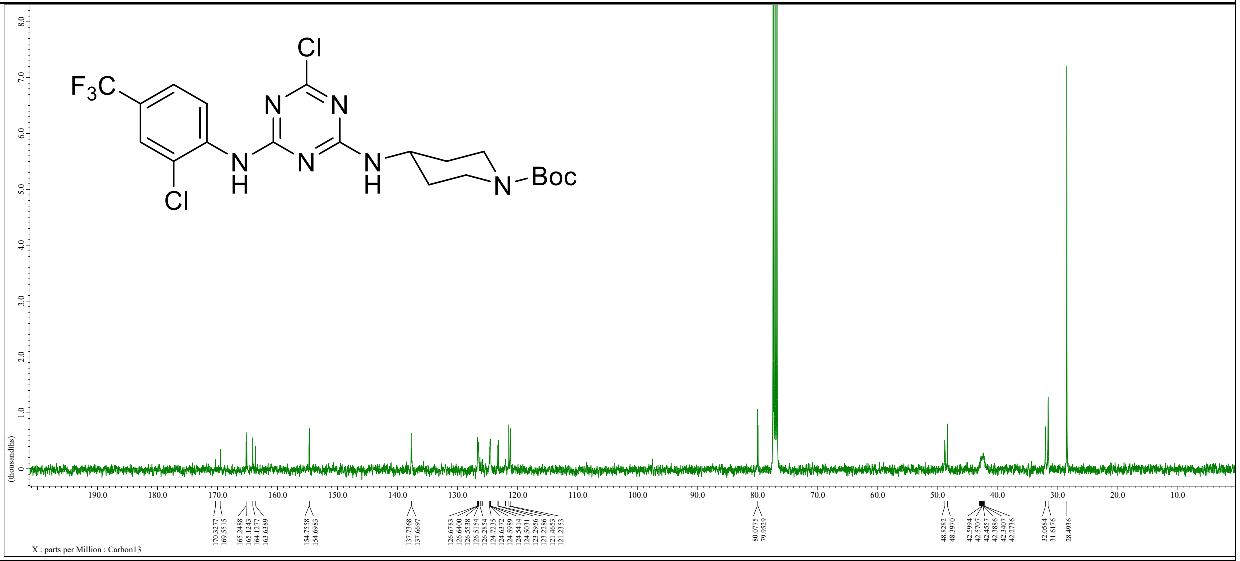

^{13}C-NMR (101 MHz, $CDCl_3$) (mixture of rotamers) δ 170.3 – 169.6 (m, 1C, 1,3,5-triazine), 165.2 – 165.1 (m, 1C, 1,3,5-triazine), 164.1 – 163.6 (m, 1C, 1,3,5-triazine), 154.8 – 154.7 (m, 1C, Boc), 137.7 (s, 1C, Ar), 126.7 – 126.5 (m, 1C, Ar), 126.3 – 125.8 (m, 1C, Ar), 124.6 – 124.5 (m, 1C, Ar), 123.4 (d, J = 272.9 Hz, 1C, CF_3), 123.3 – 123.2 (m, 1C, Alkyl), 121.5 – 121.2 (m, 1C, Alkyl), 80.1 – 80.0 (m, 1C, Boc), 48.8 – 48.4 (m, 2C, NCH_2), 43.0 – 42.2 (m, 1C, NCH), 32.1 – 31.6 (m, 2C, CCH_2), 28.5 (s, 3C, Boc)

16. ^{1}H and ^{13}C-NMR spectrum of *tert*-butyl 4-(4-chloro-6-((2-chloro-4-(trifluoromethyl)phenyl)amino)-1,3,5-triazin-2-yl)piperazine-1-carboxylate (**3l**)

^{1}H-NMR

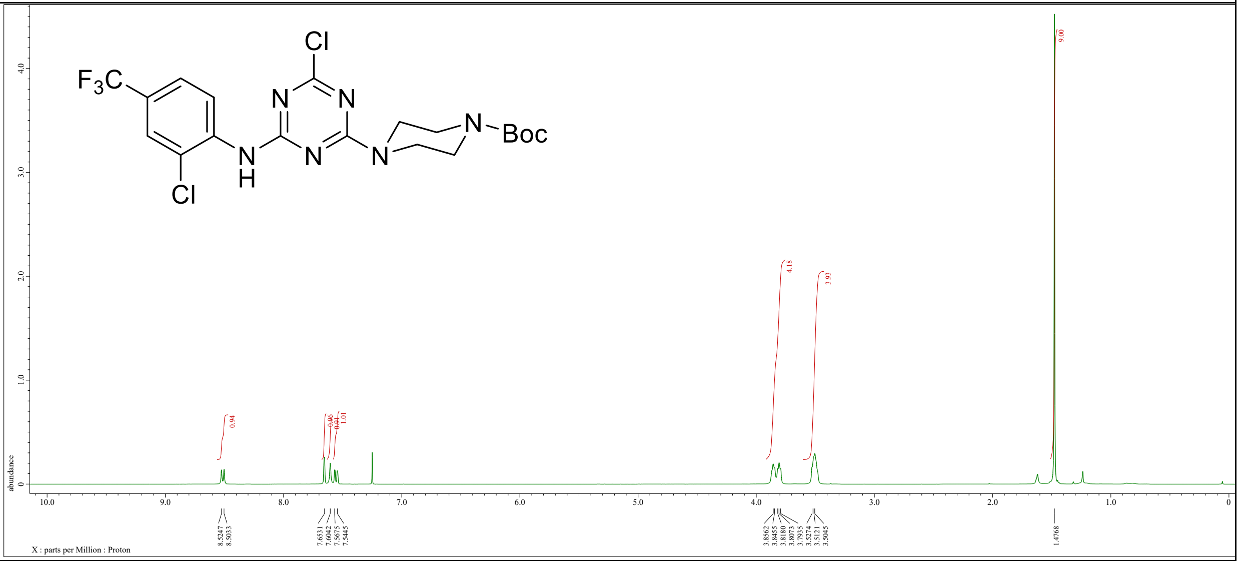

^{1}H-NMR (400 MHz, $CDCl_3$) δ 8.51 (d, J = 8.6 Hz, 1H, ArH), 7.65 (s, 1H, ArH), 7.60 (s, 1H, NH), 7.56 (d, J = 9.2 Hz, 1H, ArH), 3.86 – 3.79 (m, 4H, NCH_2), 3.53 – 3.50 (m, 4H, NCH_2), 1.48 (s, 9H, Boc)

^{13}C-NMR

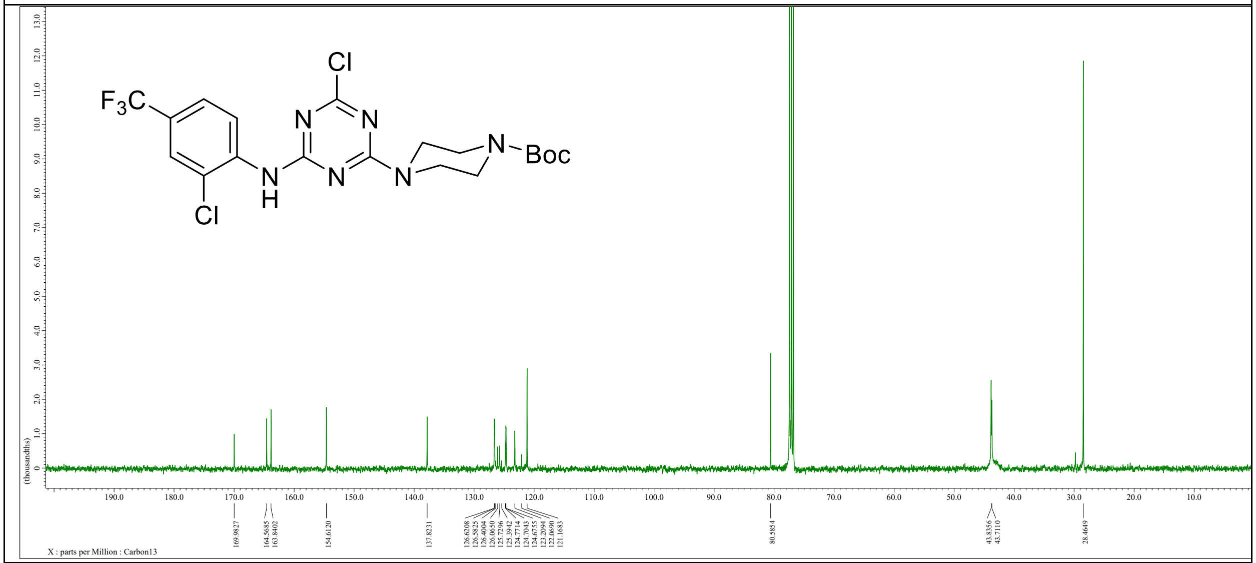

^{13}C-NMR (101 MHz, $CDCl_3$) δ 170.0 (s, 1C, 1,3,5-triazine), 164.6 (s, 1C, 1,3,5-triazine), 163.8 (s, 1C, 1,3,5-triazine), 154.6 (s, 1C, Boc), 137.8 (s, 1C, Ar), 126.6 (d, J = 3.9 Hz, 1C, Ar, C *meta*-CCF_3), 125.9 (d, J = 33.9 Hz, 1C, Ar, C *ortho*-CCF_3), 124.9 (d, J = 2.9 Hz, 1C, Ar, C *meta*-CCF_3), 123.4 (d, J = 272.9 Hz, 1C, CF_3), 123.2 (s, 1C, Ar), 121.2 (s, 1C, Ar), 80.6 (s, 1C, Boc), 43.8 (s, 2C, NCH_2), 43.7 (s, 2C, NCH_2), 28.5 (s, 3C, Boc)

17. ^{1}H and ^{13}C-NMR spectrum of (1*R*,5*S*)-*tert*-butyl 8-(4-chloro-6-((2-fluoro-4-(trifluoromethyl)phenyl)amino)-1,3,5-triazin-2-yl)-3,8-diazabicyclo[3.2.1]octane-3-carboxylate (**3m**)

^{1}H-NMR

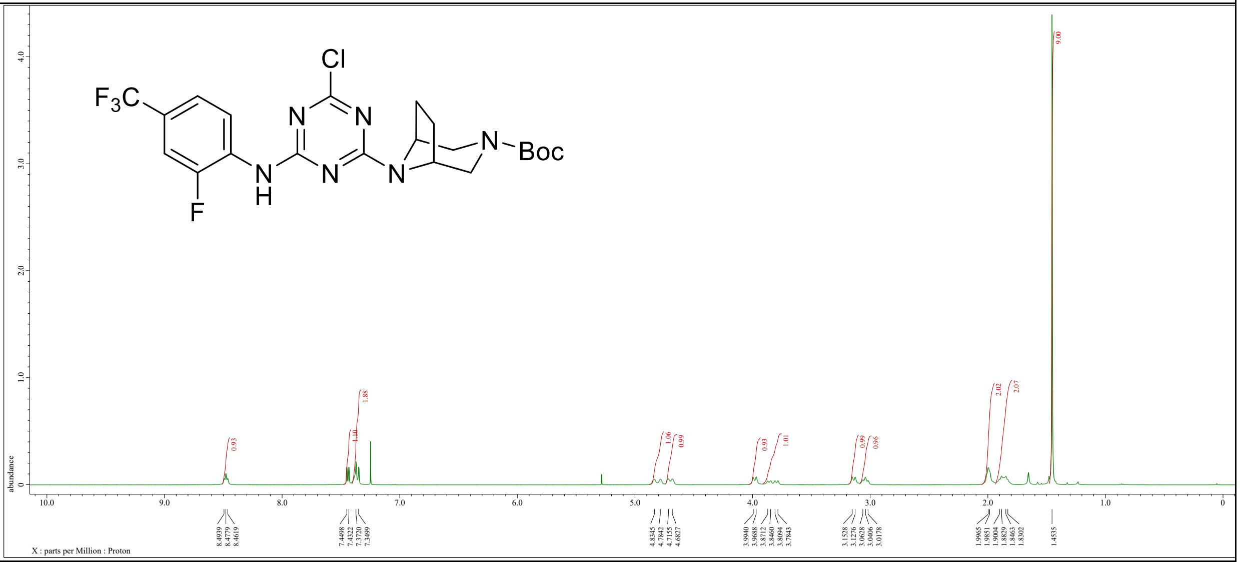

^{1}H-NMR (500 MHz, $CDCl_3$) δ 8.48 (t, *J* = 8.0 Hz, 1H, ArH), 7.44 (d, *J* = 8.8 Hz, 1H, ArH), 7.37 – 7.35 (m, 2H, ArH, NH), 4.83 – 4.78 (m, 1H, NCH), 4.72 – 4.68 (m, 1H, NCH), 3.99 – 3.97 (m, 1H, NCH_2), 3.87 – 3.78 (m, 1H, NCH_2), 3.14 (d, *J* = 12.6 Hz, 1H, NCH_2), 3.04 (t, *J* = 11.3 Hz, 1H, NCH_2), 1.99 (d, *J* = 5.7 Hz, 2H, CCH_2), 1.90 – 1.83 (m, 2H, CCH_2), 1.45 (s, 9H, Boc)

^{13}C-NMR

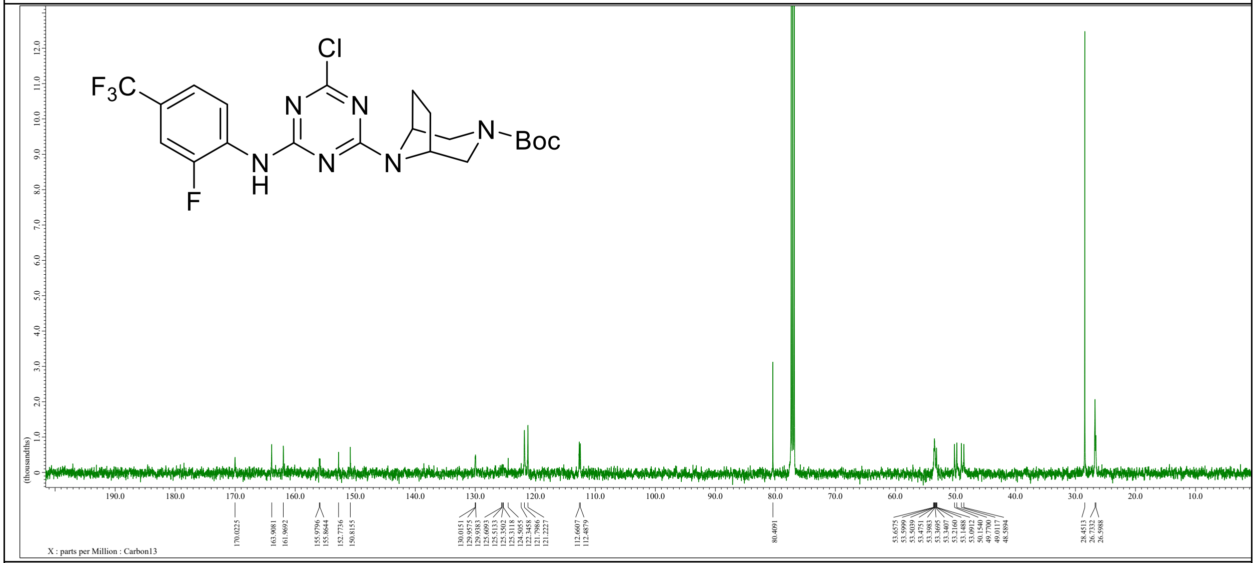

^{13}C-NMR (126 MHz, $CDCl_3$) (mixture of rotamers) δ 170.0 (s, 1C, 1,3,5-triazine), 163.9 (s, 1C, 1,3,5-triazine), 162.0 (s, 1C, 1,3,5-triazine), 156.0 – 155.9 (m, 1C, Boc), 151.8 (d, *J* = 246.7 Hz, 1C, Ar, C-F), 130.0 – 129.9 (m, 1C, Ar), 125.5 (m, 1C, Ar), 123.4 (d, *J* = 272.1 Hz, 1C, CF_3), 121.8 (s, 1C, Ar), 121.2 (s, 1C, Ar), 112.6 (d, *J* = 21.8 Hz, 1C, Ar, C *ortho*-CF), 80.4 (s, 1C, Boc), 53.7 – 53.1 (m, 2C, NCH), 50.2 – 48.6 (m, 2C, NCH_2), 28.5 (s, 3C, Boc), 26.7 – 26.6 (m, 2C, CCH_2)

18. ^{1}H and ^{13}C-NMR spectrum of (1*R*,5*S*)-*tert*-butyl 3-(4-chloro-6-((2-fluoro-4-(trifluoromethyl)phenyl)amino)-1,3,5-triazin-2-yl)-3,8-diazabicyclo[3.2.1]octane-8-carboxylate (**3n**)

^{1}H-NMR

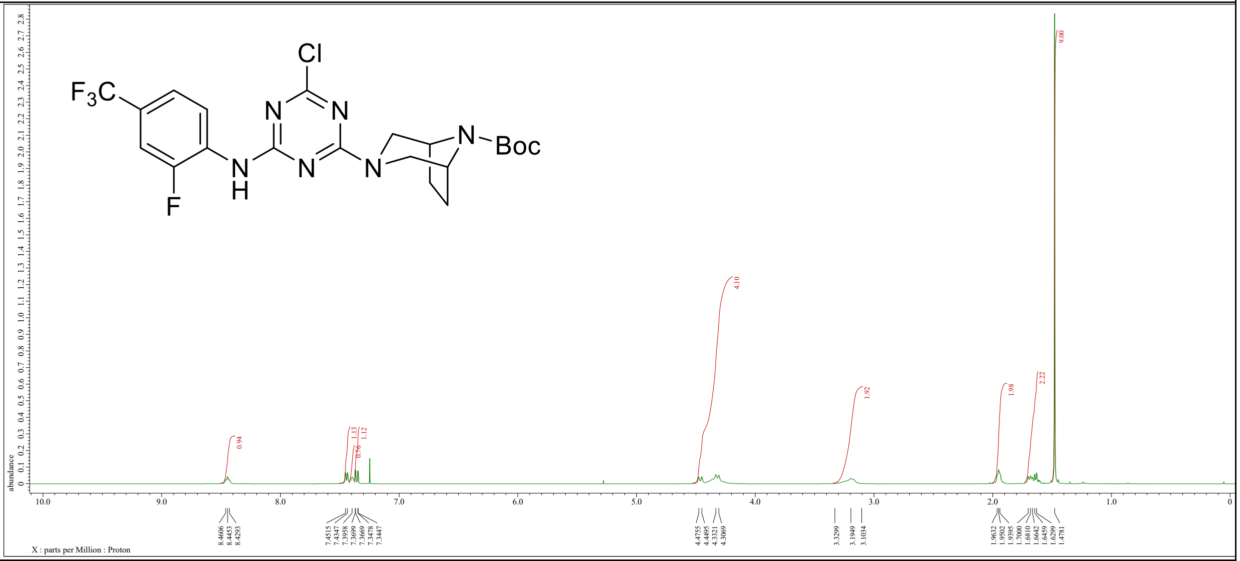

^{1}H-NMR (500 MHz, $CDCl_3$) δ 8.44 (t, *J* = 7.8 Hz, 1H, ArH), 7.44 (d, *J* = 8.4 Hz, 1H, ArH), 7.40 (s, 1H, NH), 7.36 (dd, *J* = 11.1, 1.5 Hz, 1H, ArH, H *ortho*-CF), 4.48 – 4.31 (m, 4H, NCH, NCH_2), 3.33 – 3.10 (m, 2H, NCH_2), 1.95 (t, *J* = 5.9 Hz, 2H, CCH_2), 1.70 – 1.63 (m, 2H, CCH_2), 1.48 (s, 9H, Boc)

^{13}C-NMR

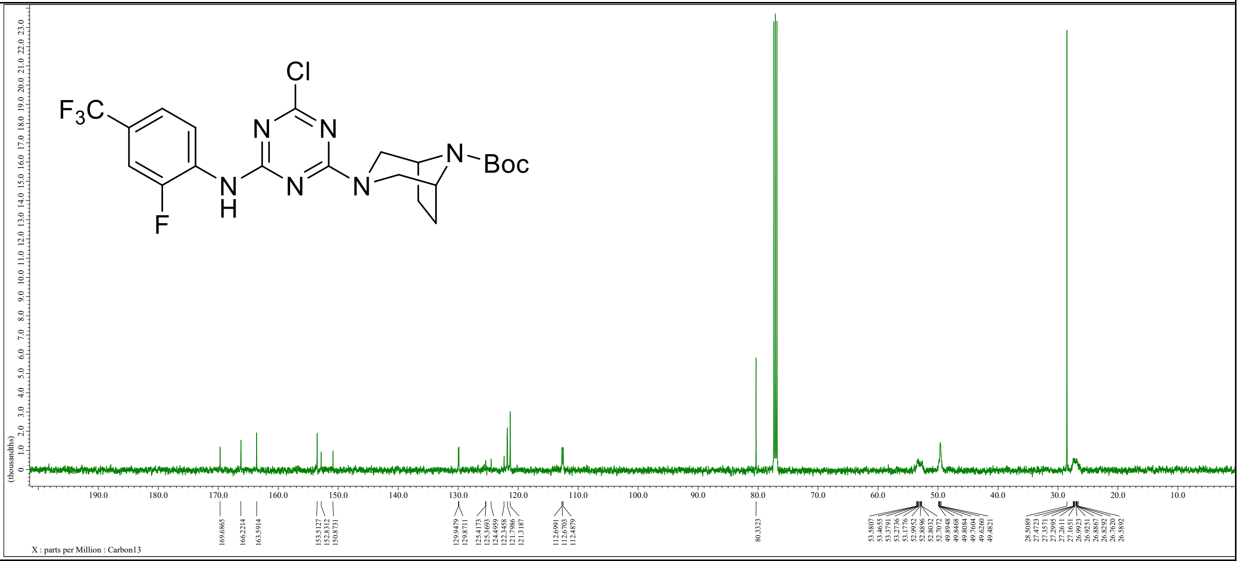

^{13}C-NMR (126 MHz, $CDCl_3$) (mixture of rotamers) δ 169.7 (s, 1C, 1,3,5-triazine), 166.2 (s, 1C, 1,3,5-triazine), 163.6 (s, 1C, 1,3,5-triazine), 153.5 (s, 1C, Boc), 151.9 (d, *J* = 246.7 Hz, 1C, Ar, C-F), 129.9 (d, *J* = 9.7 Hz, 1C, Ar, C *ortho*-CF), 125.4 (d, *J* = 6.0 Hz, 1C, Ar), 123.4 (d, *J* = 270.9 Hz, 1C, CF_3), 121.8 (s, 1C, Ar), 121.3 (s, 1C, Ar), 112.6 (d, *J* = 26.6 Hz, 1C, Ar, C *ortho*-CF), 80.3 (s, 1C, Boc), 53.6 – 52.7 (m, 2C, NCH), 49.9 – 49.5 (m, 2C, NCH_2), 28.5 (s, 3C, Boc), 27.5 – 26.6 (m, 2C, CCH_2)

19. ^{1}H and ^{13}C-NMR spectrum of *tert*-butyl 4-((4-chloro-6-((2-fluoro-4-(trifluoromethyl)phenyl)amino)-1,3,5-triazin-2-yl)amino)piperidine-1-carboxylate (**3o**)

^{1}H-NMR

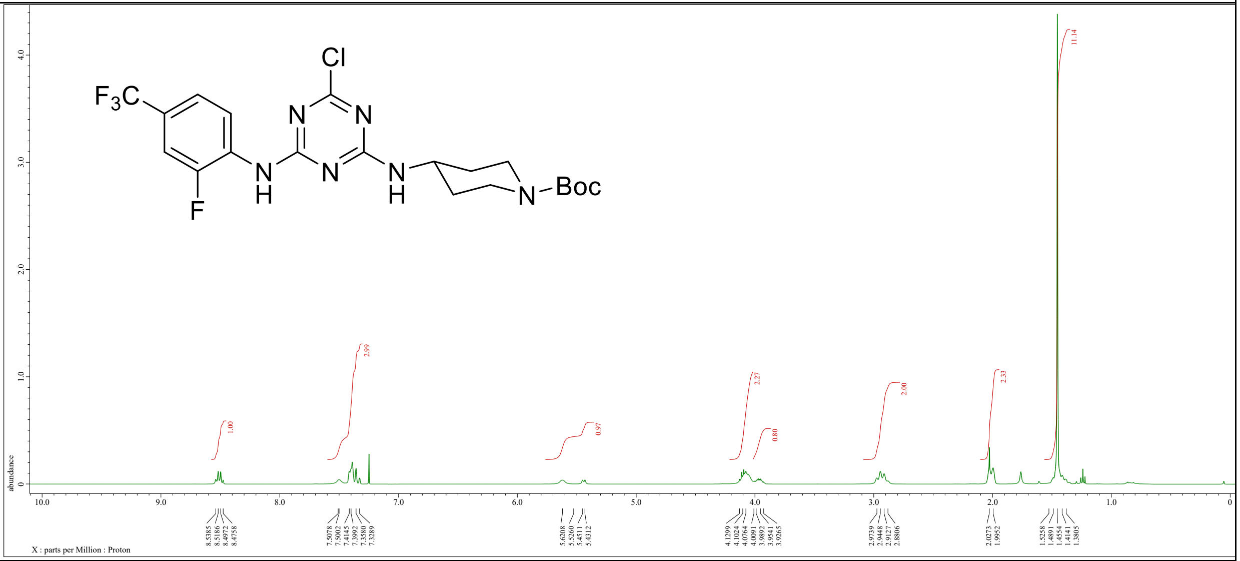

^{1}H-NMR (400 MHz, $CDCl_3$) (mixture of rotamers) δ 8.54 – 8.48 (m, 1H, ArH), 7.51 – 7.33 (m, 3H, ArH, NH), 5.62 – 5.43 (m, 1H, NH piperidine), 4.13 – 4.08 (m, 2H, NCH_2), 4.01 – 3.93 (m, 1H, NCH), 2.97 – 2.88 (m, 2H, NCH_2), 2.01 (d, J = 12.8 Hz, 2H, CCH_2), 1.53 – 1.38 (m, 11H, CCH_2, Boc)

^{13}C-NMR

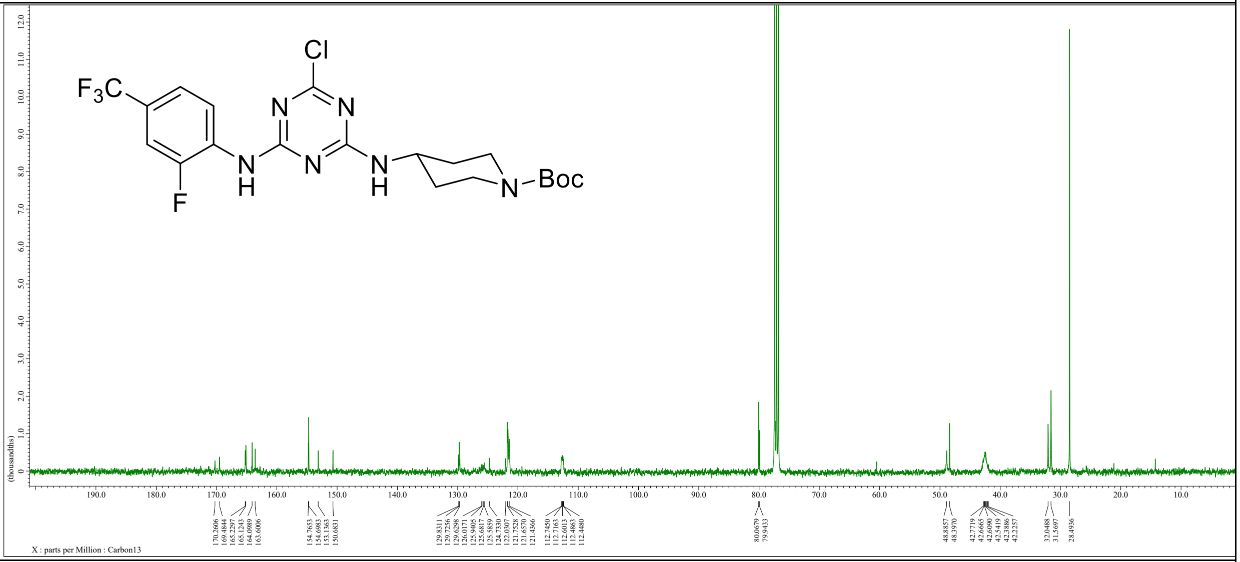

^{13}C-NMR (101 MHz, $CDCl_3$) (mixture of rotamers) δ 170.3 – 169.5 (m, 1C, 1,3,5-triazine), 165.2 – 165.1 (m, 1C, 1,3,5-triazine), 164.1 – 163.6 (m, 1C, 1,3,5-triazine), 154.8 – 154.7 (m, 1C, Boc), 151.9 (d, J = 247.8 Hz, 1C, Ar, C-F), 129.8 – 129.6 (m, 1C, Ar), 126.0 – 125.6 (m, 1C, Ar), 123.4 (d, J = 272.9 Hz, 1C, CF_3), 121.8 (s, 1C, Ar), 121.5 (d, J = 22.3 Hz, 1C, Ar, C *meta*-CF), 112.7 – 112.4 (m, 1C, Ar), 80.1 – 79.9 (m, 1C, Boc), 48.9 – 48.4 (m, 2C, NCH_2), 42.8 – 42.0 (m, 1C, NCH), 32.0 – 31.6 (m, 2C, CCH_2), 28.5 (s, 3C, Boc)

20. ^{1}H and ^{13}C-NMR spectrum of *tert*-butyl 4-(4-chloro-6-((2-fluoro-4-(trifluoromethyl)phenyl)amino)-1,3,5-triazin-2-yl)piperazine-1-carboxylate (**3p**)

^{1}H-NMR

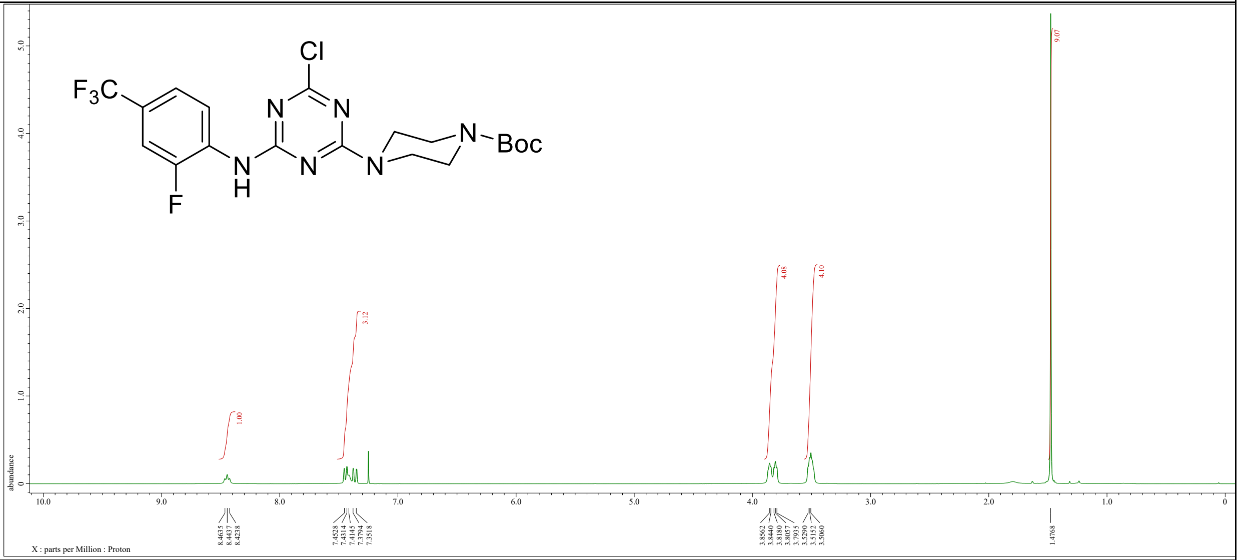

^{1}H-NMR (400 MHz, $CDCl_3$) δ 8.44 (t, J = 7.9 Hz, 1H, ArH), 7.45 – 7.35 (m, 3H, ArH, NH), 3.86 – 3.79 (m, 4H, NCH_2), 3.53 – 3.51 (m, 4H, NCH_2), 1.48 (s, 9H, Boc)

^{13}C-NMR

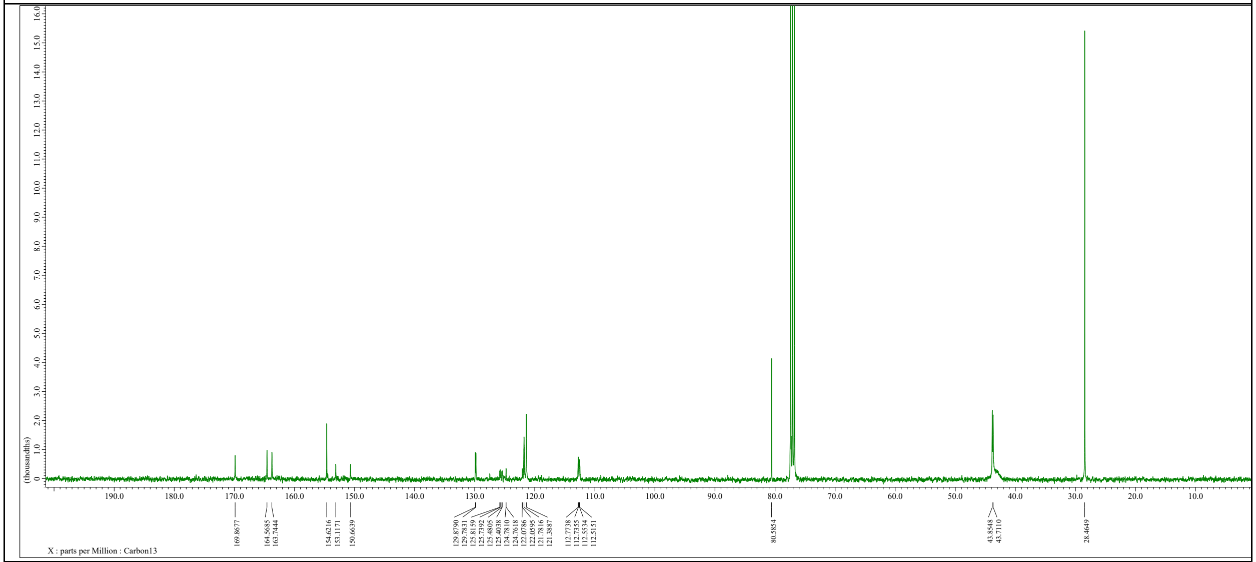

^{13}C-NMR (101 MHz, $CDCl_3$) δ 169.9 (s, 1C, 1,3,5-triazine), 164.6 (s, 1C, 1,3,5-triazine), 163.7 (s, 1C, 1,3,5-triazine), 154.6 (s, 1C, Boc), 151.9 (d, J = 247.8 Hz, 1C, Ar, C-F), 129.8 (d, J = 9.7 Hz, 1C, Ar, C *ortho*-CF), 125.8 – 125.4 (m 1C, Ar), 123.4 (dd, J = 272.9, 2.0 Hz, 1C, CF_3), 121.8 (s, 1C, Ar), 121.4 (s, 1C, Ar), 112.6 (dd, J = 22.3, 3.9 Hz, 1C, Ar, C *ortho*-CF *ortho*-CCF_3), 80.6 (s, 1C, Boc), 43.9 (s, 2C, NCH_2), 43.7 (s, 2C, NCH_2), 28.5 (s, 3C, Boc)

21. ^{1}H and ^{13}C-NMR spectrum of (1*R*,5*S*)-*tert*-butyl 8-(4-((4-cyano-2-fluorophenyl)amino)-1,3,5-triazin-2-yl)-3,8-diazabicyclo[3.2.1]octane-3-carboxylate (**4a**)

^{1}H-NMR

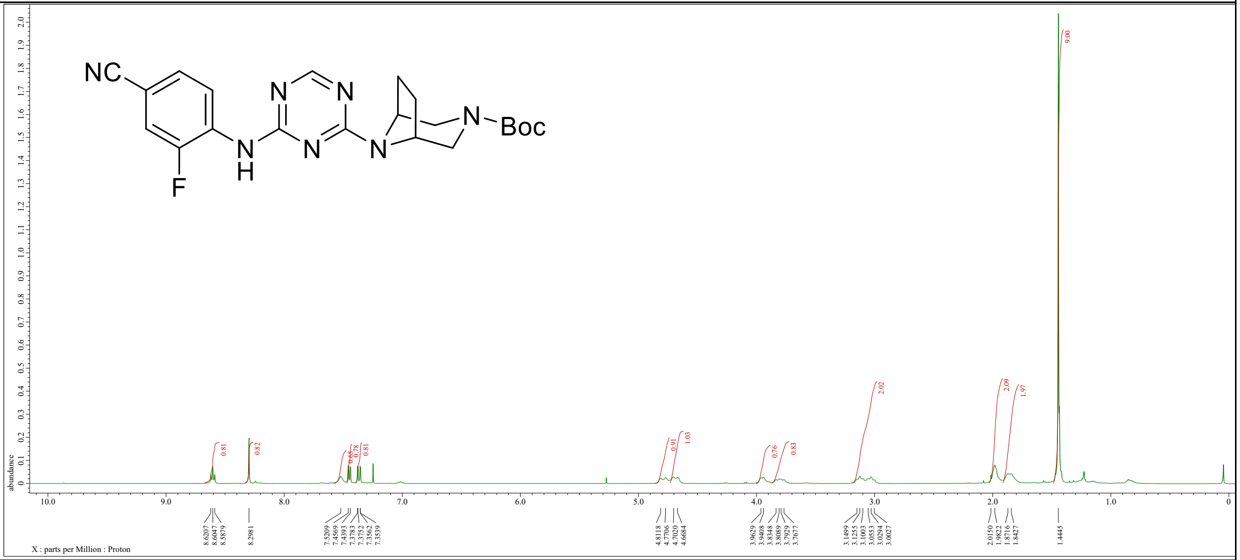

^{1}H-NMR (500 MHz, $CDCl_3$) δ 8.60 (t, J = 8.2 Hz, 1H, ArH), 8.30 (s, 1H, 1,3,5-triazine-H), 7.52 (s, 1H, NH), 7.45 (d, J = 8.8 Hz, 1H, ArH), 7.37 (dd, J = 10.9, 1.3 Hz, 1H, ArH, H *ortho*-CF), 4.79 (d, J = 20.6 Hz, 1H, NCH), 4.69 (d, J = 16.8 Hz, 1H, NCH), 3.95 (d, J = 11.1 Hz, 1H, NCH_2), 3.80 (dd, J = 20.8, 12.8 Hz, 1H, NCH_2), 3.15 – 3.00 (m, 2H, NCH_2), 2.00 (d, J = 16.4 Hz, 2H, CCH_2), 1.86 (d, J = 14.5 Hz, 2H, CCH_2), 1.44 (s, 9H, Boc)

^{13}C-NMR

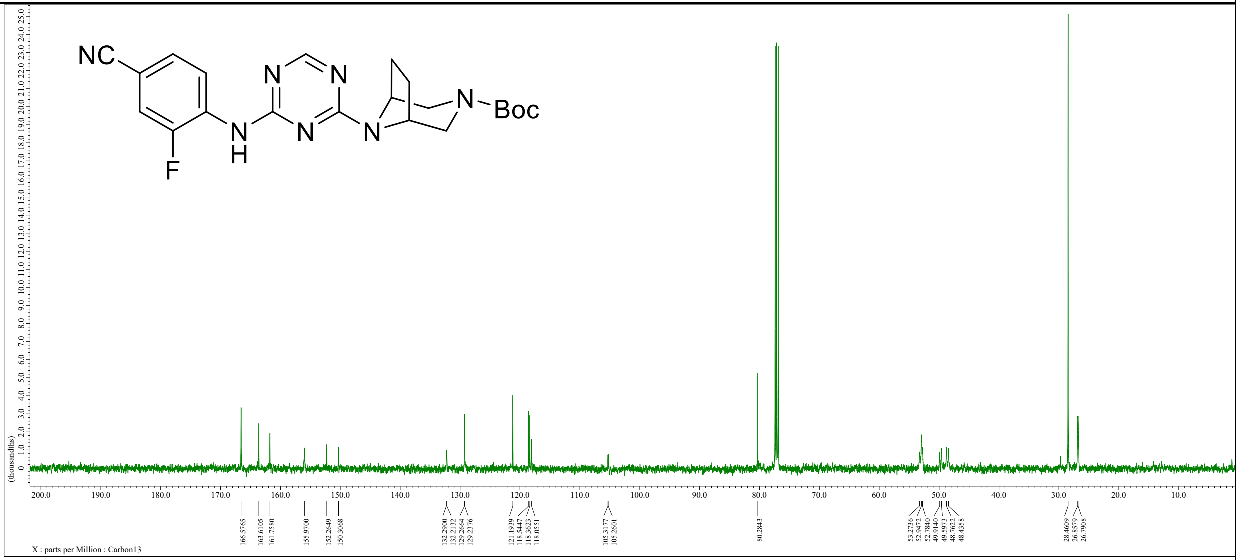

^{13}C-NMR (126 MHz, $CDCl_3$) (mixture of rotamers) δ 166.6 (s, 1C, 1,3,5-triazine), 163.6 (s, 1C, 1,3,5-triazine), 161.8 (s, 1C, 1,3,5-triazine), 156.0 (s, 1C, Boc), 151.3 (d, J = 246.7 Hz, 1C, Ar, C-F), 132.3 (d, J = 9.7 Hz, 1C, Ar, C *meta*-CF), 129.3 (d, J = 3.5 Hz, 1C, Ar), 121.2 (s, 1C, Ar), 118.5 (d, J = 23.1 Hz, 1C, Ar, *ortho*-CF), 118.1 (s, 1C, CN), 105.3 (d, J = 7.3 Hz, 1C, Ar, C-CN *meta*-CF), 80.3 (s, 1C, Boc), 53.3 – 52.8 (m, 2C, NCH), 49.9 – 48.4 (m, 2C, NCH_2), 28.5 (s, 3C, Boc), 26.9 – 26.8 (m, 2C, CCH_2)

22. ^{1}H and ^{13}C-NMR spectrum of (1*R*,5*S*)-*tert*-butyl 3-(4-((4-cyano-2-fluorophenyl)amino)-1,3,5-triazin-2-yl)-3,8-diazabicyclo[3.2.1]octane-8-carboxylate (**4b**)

^{1}H-NMR

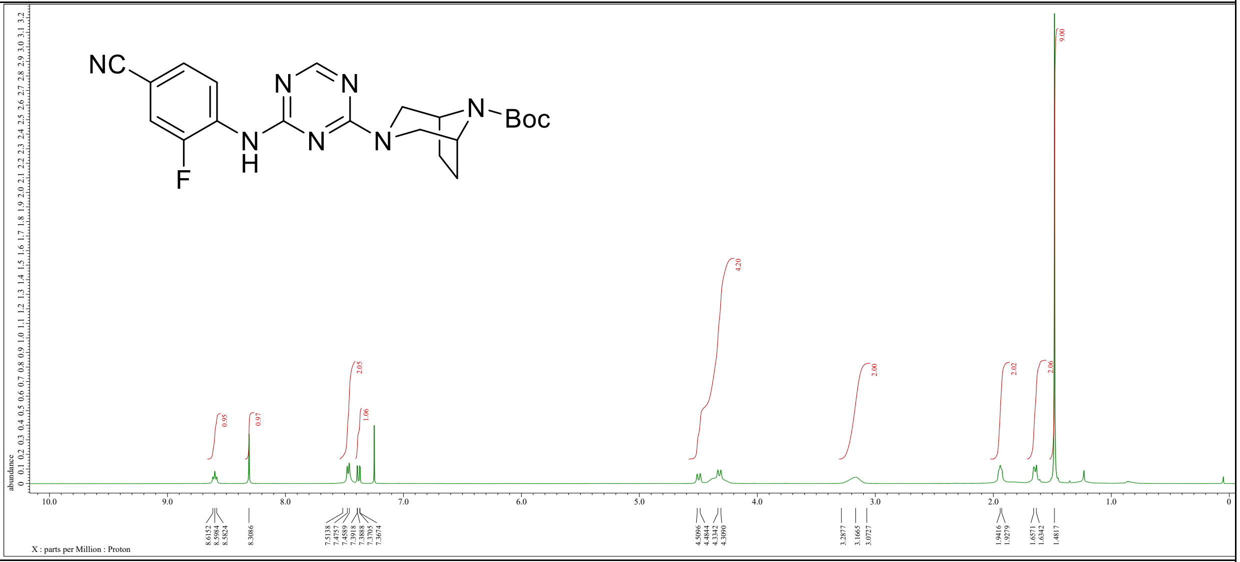

^{1}H-NMR (500 MHz, $CDCl_3$) δ 8.60 (t, J = 8.2 Hz, 1H, ArH), 8.31 (s, 1H, 1,3,5-triazine-H), 7.51 – 7.46 (m, 2H, ArH, NH), 7.38 (dd, J = 10.7, 1.5 Hz, 1H, ArH, H *ortho*-CF), 4.51 – 4.31 (m, 4H, NCH, NCH_2), 3.29 – 3.07 (m, 2H, NCH_2), 1.94 – 1.93 (m, 2H, CCH_2), 1.65 (d, J = 11.4 Hz, 2H, CCH_2), 1.48 (s, 9H, Boc)

^{13}C-NMR

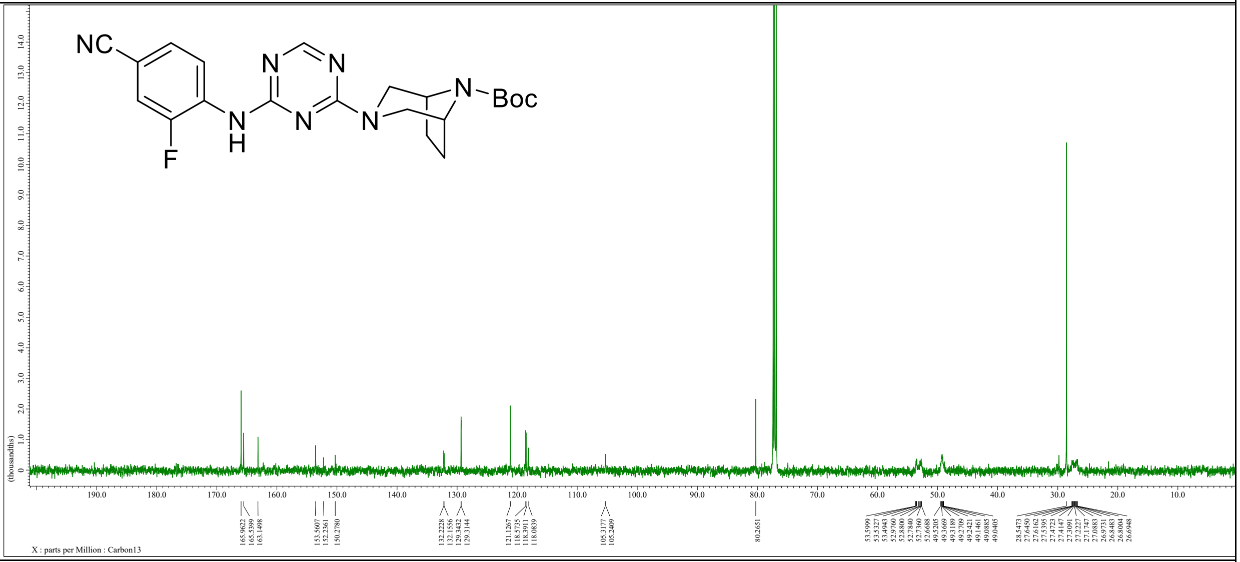

^{13}C-NMR (126 MHz, $CDCl_3$) (mixture of rotamers) δ 166.0 (s, 1C, 1,3,5-triazine), 165.5 (s, 1C, 1,3,5-triazine), 163.1 (s, 1C, 1,3,5-triazine), 153.6 (s, 1C, Boc), 151.3 (d, J = 246.7 Hz, 1C, Ar, C-F), 132.2 (d, J = 8.4 Hz, 1C, Ar, C *meta*-CF), 129.3 (d, J = 3.7 Hz, 1C, Ar), 121.1 (s, 1C, Ar), 118.5 (d, J = 22.9 Hz, 1C, Ar, C *ortho*-CF), 118.1 (s, 1C, CN), 105.3 (d, J = 9.7 Hz, 1C, Ar, C *meta*-CF), 80.3 (s, 1C, Boc), 53.7 – 52.7 (m, 2C, NCH), 49.5 – 49.0 (m, 2C, NCH_2), 28.5 (s, 3C, Boc), 27.6 – 26.7 (m, 2C, CCH_2)

23. ^{1}H and ^{13}C-NMR spectrum of *tert*-butyl 4-((4-((4-cyano-2-fluorophenyl)amino)-1,3,5-triazin-2-yl)amino)piperidine-1-carboxylate (**4c**)

^{1}H-NMR

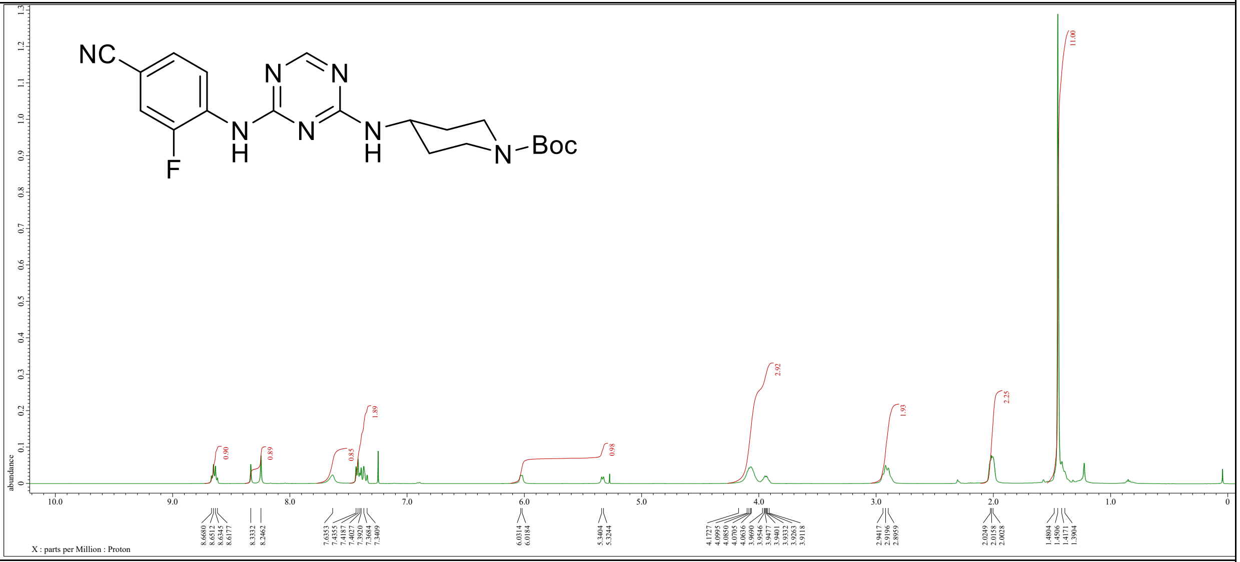

^{1}H-NMR (500 MHz, $CDCl_3$) (mixture of rotamers) δ 8.64 (q, J = 8.4 Hz, 1H, ArH), 8.33 – 8.25 (m, 1H, 1,3,5-triazine-H), 7.64 (s, 1H, NH), 7.44 – 7.34 (m, 2H, ArH), 6.03 – 5.32 (m, 1H, NH piperidine), 4.17 – 3.91 (m, 3H, NCH, NCH_2), 2.92 (t, J = 11.4 Hz, 2H, NCH_2), 2.02 – 2.00 (m, 2H, CCH_2), 1.48 – 1.39 (m, 11H, CCH_2, Boc)

^{13}C-NMR

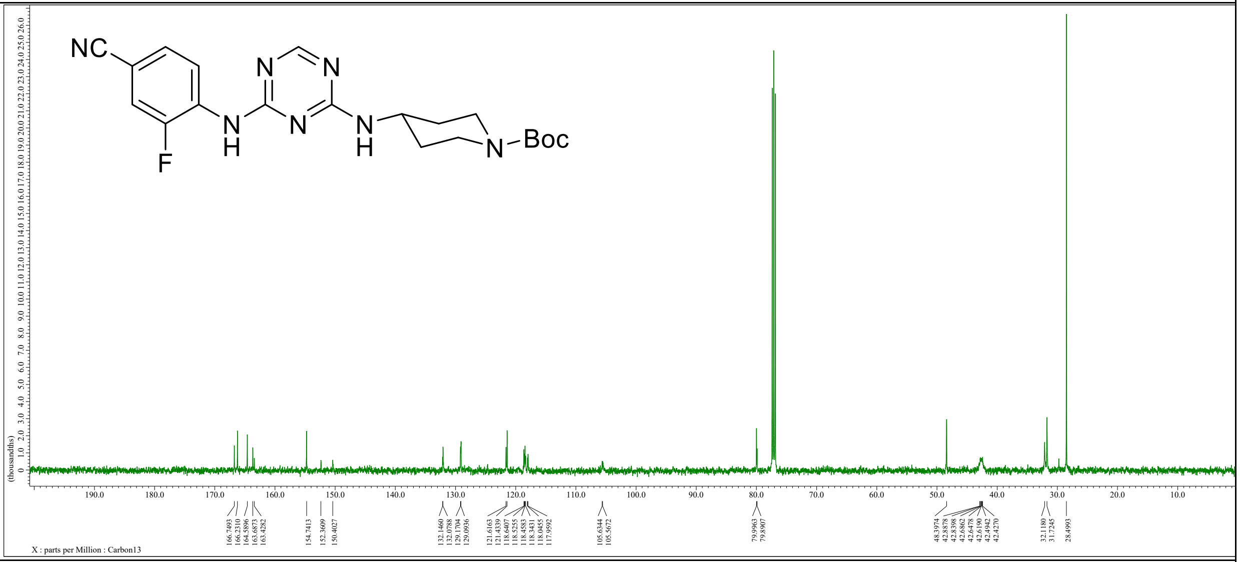

^{13}C-NMR (126 MHz, $CDCl_3$) (mixture of rotamers) δ 166.7 – 166.2 (m, 1C, 1,3,5-triazine), 164.6 (s, 1C, 1,3,5-triazine), 163.7 – 163.4 (m, 1C, 1,3,5-triazine), 154.7 (s, 1C, Boc), 151.4 (d, J = 246.7 Hz, 1C, Ar, C-F), 132.1 (d, J = 8.4 Hz, 1C, Ar, C *meta*-CF), 129.1 (d, J = 9.6 Hz, 1C, Ar, C *meta*-CF), 121.6 – 121.5 (m, 1C, Ar), 118.6 – 118.3 (m, 1C, Ar), 118.0 – 118.0 (m, 1C, CN), 105.6 (d, J = 8.4 Hz, 1C, C-CN *meta*-CF), 80.0 – 79.9 (m, 1C, Boc), 48.4 (s, 2C, NCH_2), 42.9 – 42.4 (m, 1C, NCH), 32.1 – 31.7 (m, 2C, CCH_2), 28.5 (s, 3C, Boc)

24. ^{1}H and ^{13}C-NMR spectrum of *tert*-butyl 4-(4-((4-cyano-2-fluorophenyl)amino)-1,3,5-triazin-2-yl)piperazine-1-carboxylate (**4d**)

^{1}H-NMR

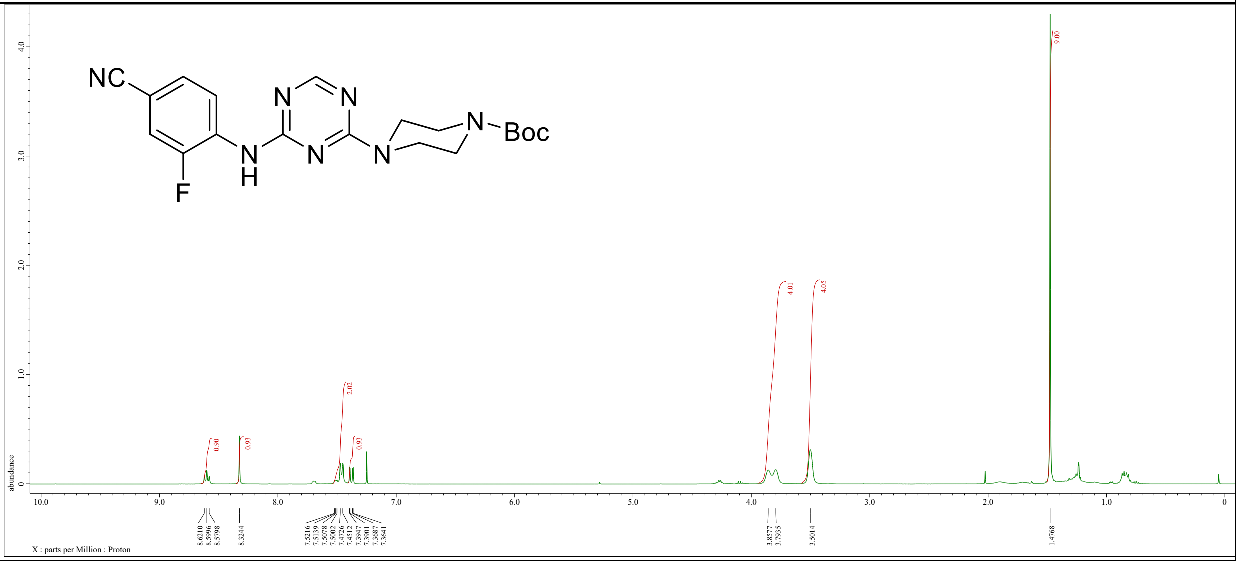

^{1}H-NMR (400 MHz, $CDCl_3$) δ 8.60 (t, J = 8.3 Hz, 1H, ArH), 8.32 (s, 1H, 1,3,5-triazine-H), 7.52 – 7.45 (m, 2H, ArH, NH), 7.38 (dd, J = 10.4, 1.8 Hz, 1H, ArH, H *ortho*-CF), 3.86 – 3.79 (m, 4H, NCH_2), 3.50 (s, 4H, NCH_2), 1.48 (s, 9H, Boc)

^{13}C-NMR

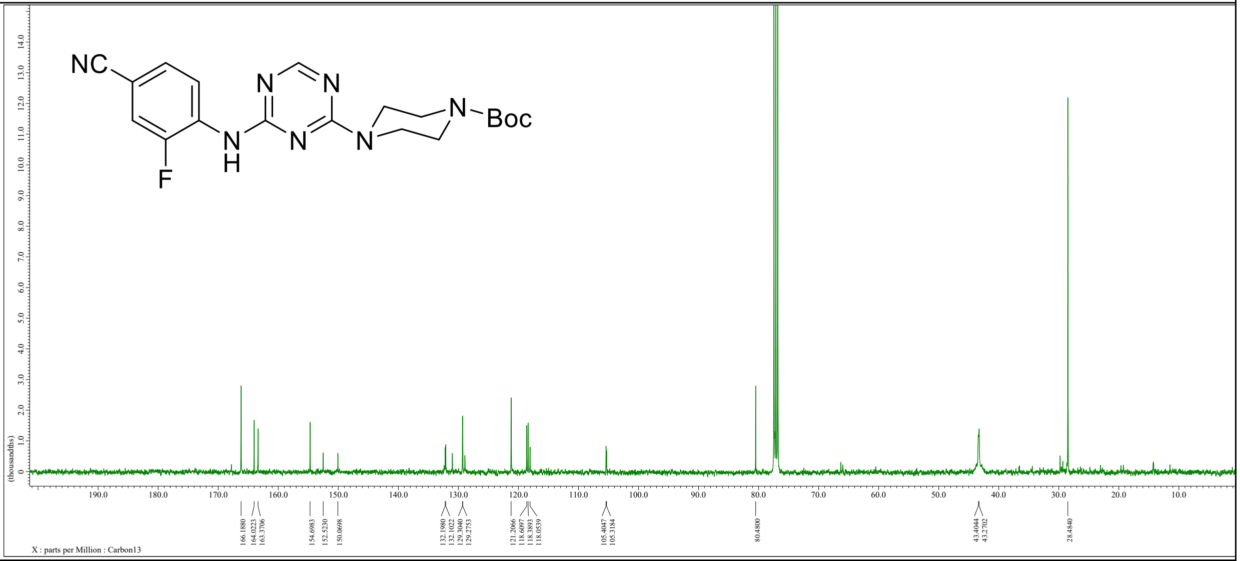

^{13}C-NMR (101 MHz, $CDCl_3$) δ 166.2 (s, 1C, 1,3,5-triazine), 164.0 (s, 1C, 1,3,5-triazine), 163.4 (s, 1C, 1,3,5-triazine), 154.7 (s, 1C, Boc), 151.3 (d, J = 247.8 Hz, 1C, Ar, C-F), 132.2 (d, J = 9.7 Hz, 1C, Ar, C *meta*-CF), 129.3 (d, J = 3.9 Hz, 1C, Ar), 121.2 (s, 1C, Ar), 118.5 (d, J = 22.3 Hz, 1C, Ar, C *ortho*-CF), 118.1 (s, 1C, CN), 105.4 (d, J = 8.8 Hz, 1C, Ar, C-CN *meta*-CF), 80.5 (s, 1C, Boc), 43.4 (s, 2C, NCH_2), 43.3 (s, 2C, NCH_2), 28.5 (s, 3C, Boc)

25. ^{1}H and ^{13}C-NMR spectrum of (1*R*,5*S*)-*tert*-butyl 8-(4-((2-fluoro-4-(methylsulfonyl)phenyl)amino)-1,3,5-triazin-2-yl)-3,8-diaza bicyclo[3.2.1]octane-3-carboxylate (**4e**)

^{1}H-NMR

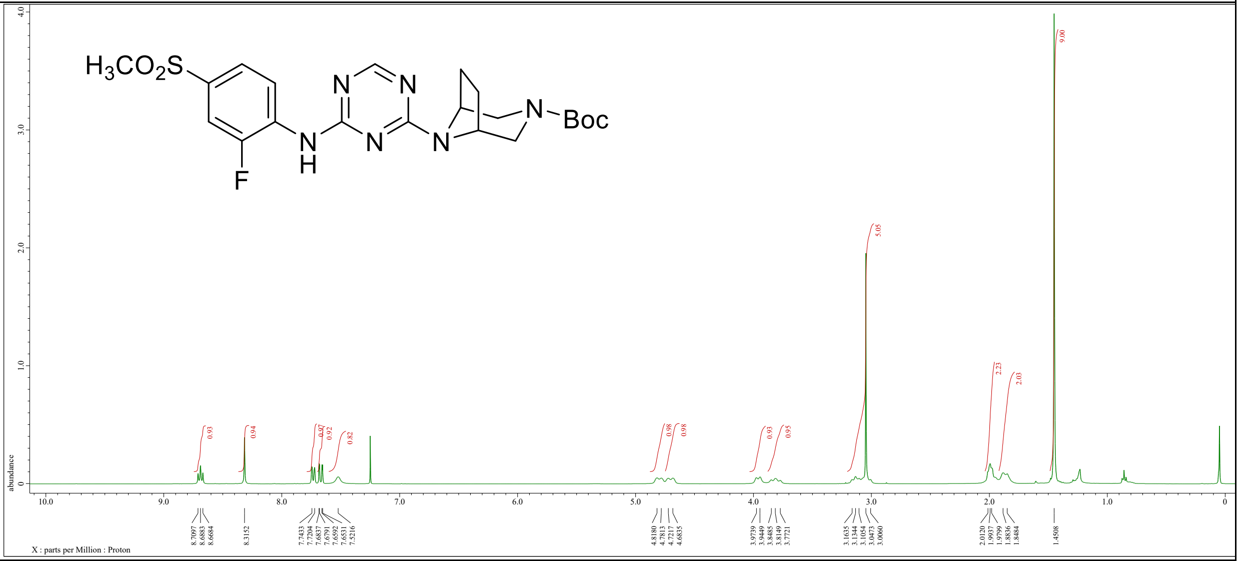

^{1}H-NMR (400 MHz, $CDCl_3$) δ 8.69 (t, *J* = 8.3 Hz, 1H, ArH), 8.32 (s, 1H, 1,3,5-triazine-H), 7.73 (d, *J* = 9.2 Hz, 1H, ArH), 7.67 (dd, *J* = 10.1, 2.1 Hz, 1H, ArH, H *ortho*-CF), 7.52 (s, 1H, NH), 4.82 – 4.78 (m, 1H, NCH), 4.72 – 4.68 (m, 1H, NCH), 3.97 – 3.94 (m, 1H, NCH_2), 3.85 – 3.77 (m, 1H, NCH_2), 3.16 – 3.01 (m, 5H, NCH_2, SO_2CH_3), 2.00 (t, *J* = 6.4 Hz, 2H, CCH_2), 1.88 – 1.85 (m, 2H, CCH_2), 1.45 (s, 9H, Boc)

^{13}C-NMR

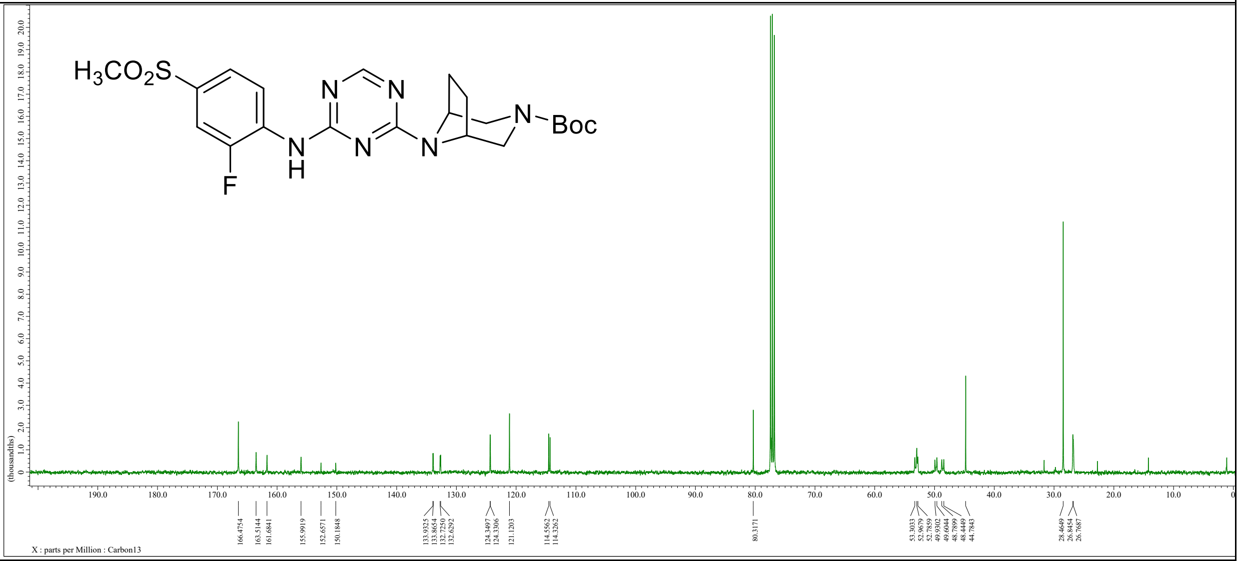

^{13}C-NMR (101 MHz, $CDCl_3$) (mixture of rotamers) δ 166.5 (s, 1C, 1,3,5-triazine), 163.5 (s, 1C, 1,3,5-triazine), 161.7 (s, 1C, 1,3,5-triazine), 156.0 (s, 1C, Boc), 151.4 (d, *J* = 249.7 Hz, Ar, C-F), 133.9 (d, *J* = 6.8 Hz, 1C, Ar, C *meta*-CF), 132.7 (d, *J* = 9.7 Hz, 1C, Ar, C *meta*-CF), 124.3 (d, *J* = 1.9 Hz, 1C, Ar), 121.1 (s, 1C, Ar), 114.4 (d, *J* = 23.2 Hz, 1C, Ar, C *ortho*-CF), 80.3 (s, 1C, Boc), 53.3 – 52.8 (m, 2C, NCH), 49.9 – 48.4 (m, 2C, NCH_2), 44.8 (s, 1C, SO_2CH_3), 28.5 (s, 3C, Boc), 26.8 – 26.8 (m, 2C, CCH_2)

26. ^{1}H and ^{13}C-NMR spectrum of (1*R*,5*S*)-*tert*-butyl 3-(4-((2-fluoro-4-(methylsulfonyl)phenyl) amino)-1,3,5-triazin-2-yl)-3,8-diazabicyclo[3.2.1]octane-8-carboxylate (**4f**)

^{1}H-NMR

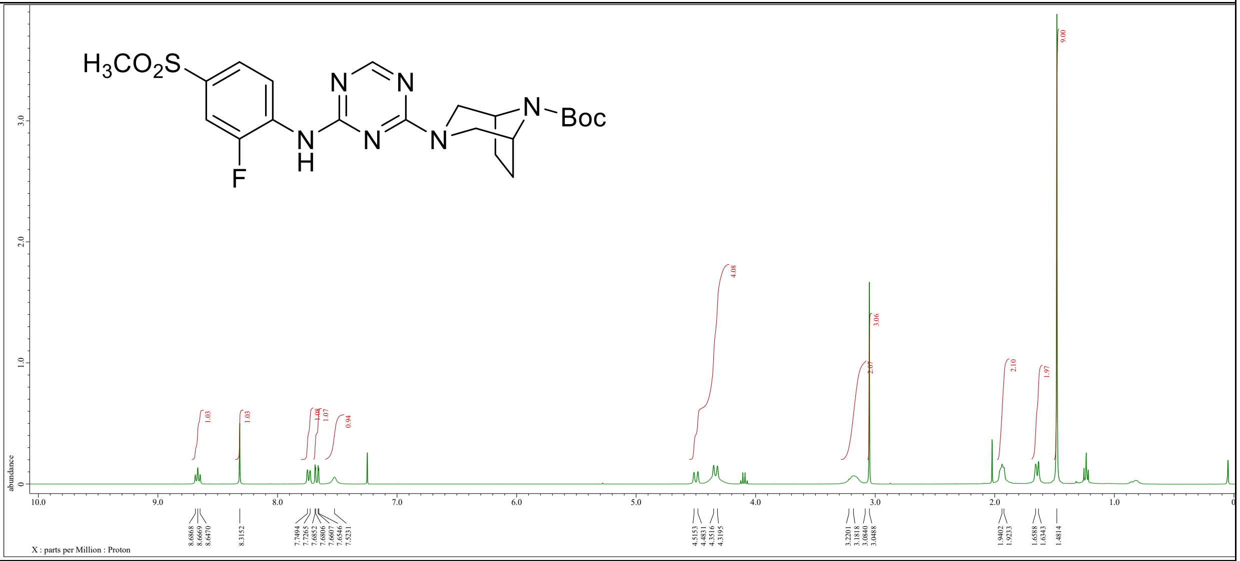

^{1}H-NMR (400 MHz, $CDCl_3$) δ 8.67 (t, J = 7.9 Hz, 1H, ArH), 8.32 (s, 1H, 1,3,5-triazine-H), 7.74 (d, J = 9.2 Hz, 1H, ArH), 7.67 (dd, J = 10.1, 2.1 Hz, 1H, ArH, H *ortho*-CF), 7.52 (s, 1H, NH), 4.52 – 4.32 (m, 4H, NCH, NCH_2), 3.22 – 3.08 (m, 2H, NCH_2), 3.05 (s, 3H, SO_2CH_3), 1.93 (d, J = 6.7 Hz, 2H, CCH_2), 1.65 (d, J = 9.8 Hz, 2H, CCH_2), 1.48 (s, 9H, Boc)

^{13}C-NMR

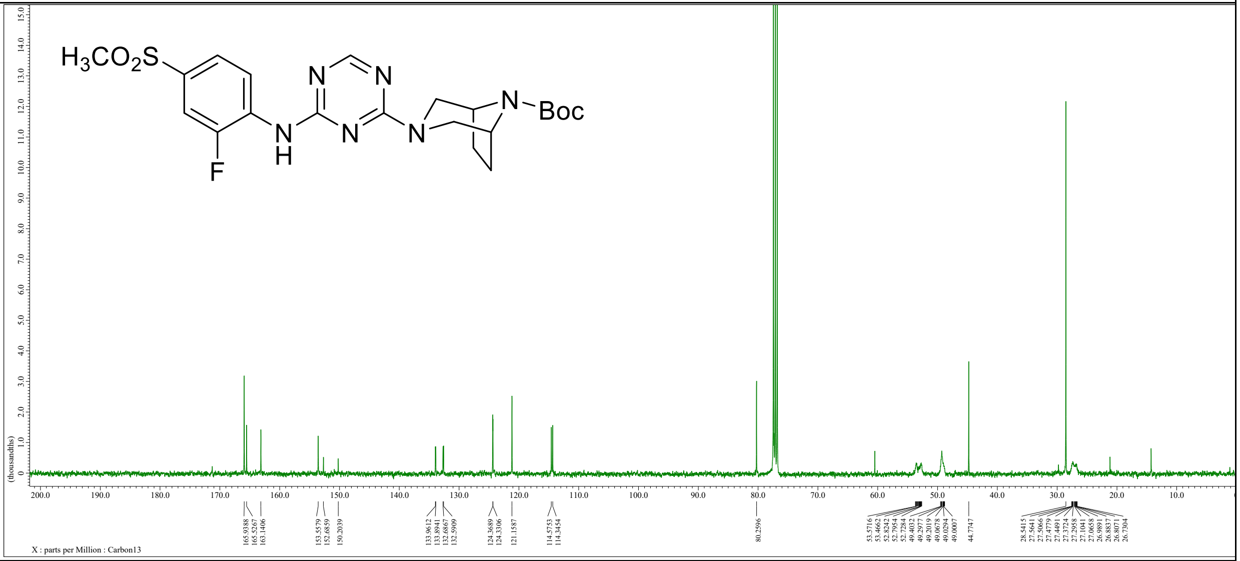

^{13}C-NMR (101 MHz, $CDCl_3$) (mixture of rotamers) δ 165.9 (s, 1C, 1,3,5-triazine), 165.5 (s, 1C, 1,3,5-triazine), 163.1 (s, 1C, 1,3,5-triazine), 153.6 (s, 1C, Boc), 151.4 (d, J = 250.7 Hz, 1C, Ar, C-F), 133.9 (d, J = 6.8 Hz, 1C, Ar, C-SO_2CH_3), 132.6 (d, J = 9.7 Hz, 1C, Ar, C *meta*-CF), 124.4 (d, J = 3.8 Hz, 1C, Ar), 121.2 (s, 1C, Ar), 114.5 (d, J = 23.2, 1C, Ar, C *ortho*-CF), 80.3 (s, 1C, Boc), 53.7 – 52.6 (m, 2C, NCH), 49.5 – 48.8 (m, 2C, NCH_2), 44.8 (s, 1C, SO_2CH_3), 28.5 (s, 3C, Boc), 27.6 – 26.6 (m, 2C, CCH_2)

27. ^{1}H and ^{13}C-NMR spectrum of *tert*-butyl 4-((4-((2-fluoro-4-(methylsulfonyl)phenyl)amino)-1,3,5-triazin-2-yl)amino)piperidine-1-carboxylate (**4g**)

^{1}H-NMR

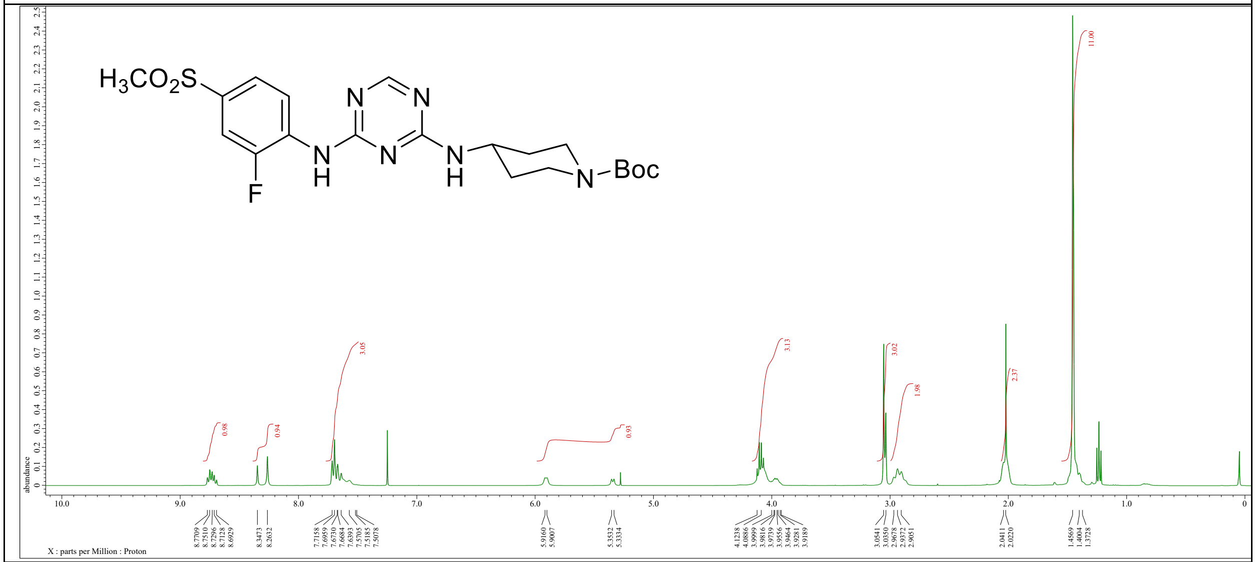

^{1}H-NMR (400 MHz, $CDCl_3$) (mixture of rotamers) δ 8.77 – 8.69 (m, 1H, H_5), 8.35 – 8.26 (m, 1H, 1,3,5-triazine-H), 7.72 – 7.51 (m, 3H, ArH, NH), 5.92 – 5.33 (m, 1H, (m, 1H, NH piperidine), 4.12 – 3.92 (m, 3H, NCH, NCH_2), 3.05 – 3.04 (m, 3H, SO_2CH_3), 2.97 – 2.91 (m, 2H, NCH_2), 2.04 – 2.02 (m, 2H, CCH_2), 1.46 – 1.37 (m, 11H, CCH_2, Boc)

^{13}C-NMR

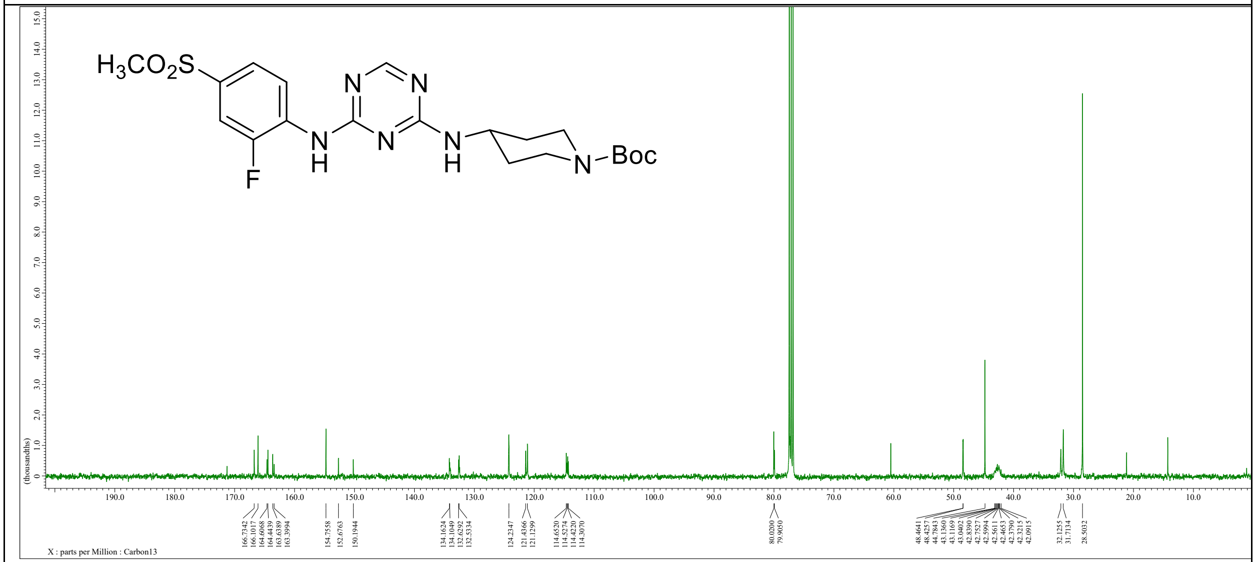

^{13}C-NMR (101 MHz, $CDCl_3$) (mixture of rotamers) δ 166.7 – 166.1 (m, 1C, 1,3,5-triazine), 164.6 – 164.4 (m, 1C, 1,3,5-triazine), 163.6 – 163.4 (m, 1C, 1,3,5-triazine), 154.8 (s, 1C, Boc), 151.4 (d, J = 250.7 Hz, 1C, Ar, C-F), 134.2 – 134.1 (m, 1C, Ar), 132.6 – 132.5 (m, 1C, Ar), 124.2 (s, 1C, Ar), 121.4 – 121.1 (m, 1C, Ar), 114.7 – 114.3 (m, 1C, Ar), 80.0 – 79.9 (m, 1C, Boc), 48.5 – 48.4 (m, 2C, NCH_2), 44.8 (s, 1C, SO_2CH_3), 43.1 – 42.1 (m, 1C, NCH), 32.1 – 31.7 (m, 2C, CCH_2), 28.5 (s, 3C, Boc)

28. ^{1}H and ^{13}C-NMR spectrum of *tert*-butyl 4-(4-((2-fluoro-4-(methylsulfonyl)phenyl)amino)-1,3,5-triazin-2-yl)piperazine-1-carboxylate (**4h**)

^{1}H-NMR

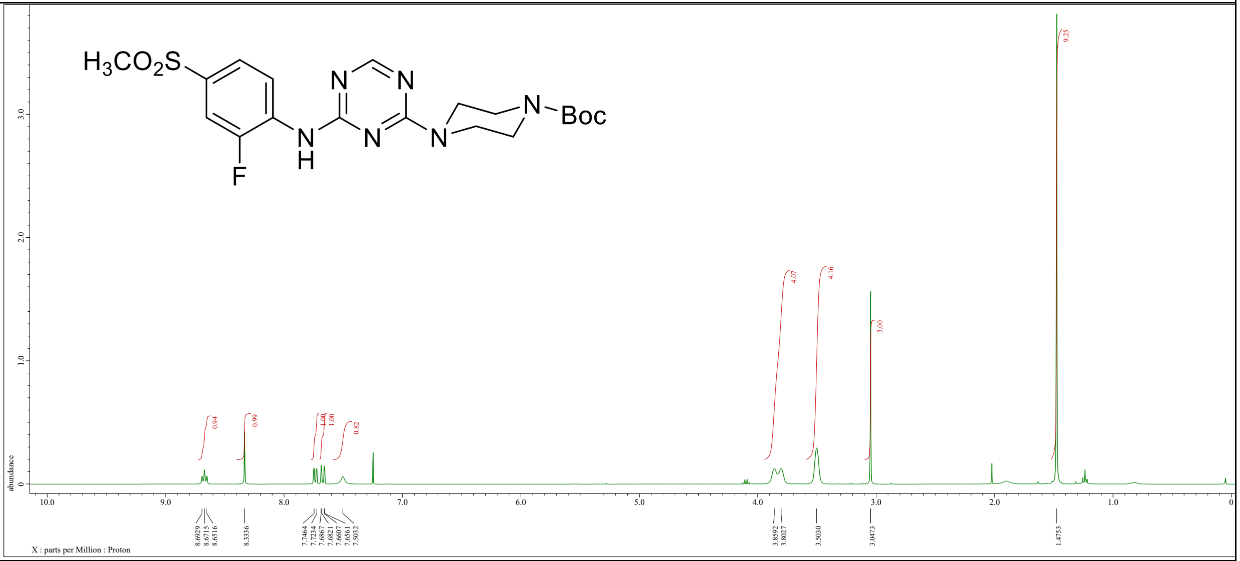

^{1}H-NMR (400 MHz, $CDCl_3$) δ 8.67 (t, J = 8.3 Hz, 1H, ArH), 8.33 (s, 1H, 1,3,5-triazine-H), 7.73 (d, J = 9.2 Hz, 1H, ArH), 7.67 (dd, J = 10.4, 1.8 Hz, 1H, ArH, H *ortho*-CF), 7.50 (s, 1H, NH), 3.86 – 3.80 (m, 4H, NCH_2), 3.50 (s, 4H, NCH_2), 3.05 (s, 3H, SO_2CH_3), 1.48 (s, 9H, Boc)

^{13}C-NMR

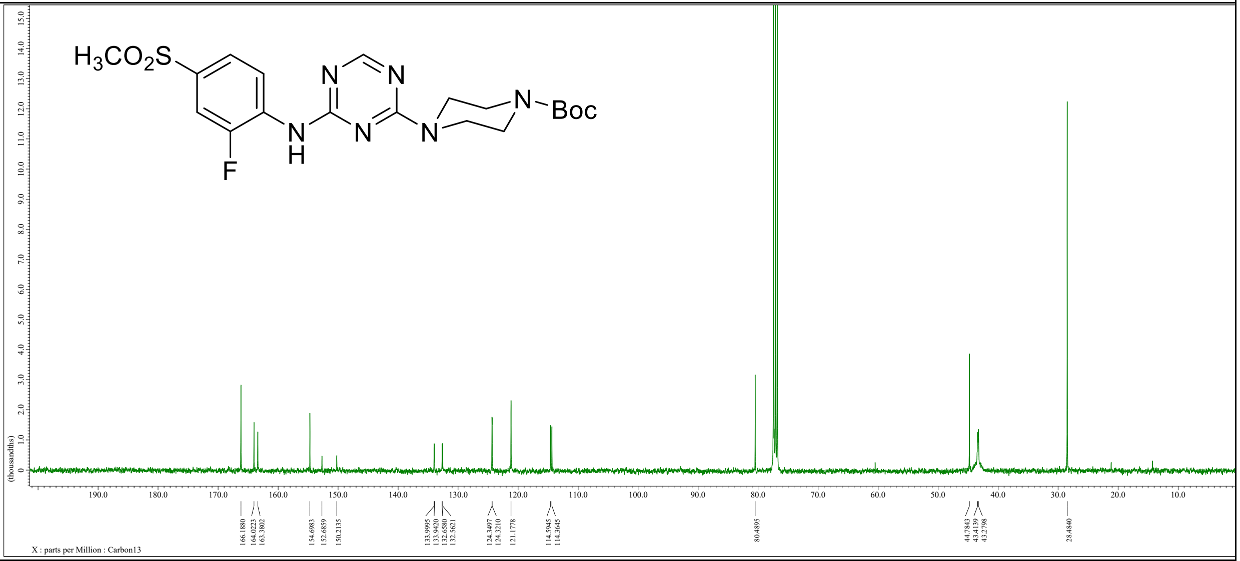

^{13}C-NMR (101 MHz, $CDCl_3$) δ 166.2 (s, 1C, 1,3,5-triazine), 164.0 (s, 1C, 1,3,5-triazine), 163.4 (s, 1C, 1,3,5-triazine), 154.7 (s, 1C, Boc), 151.5 (d, J = 249.7 Hz, 1C, Ar, C-F), 134.0 (d, J = 5.9 Hz, 1C, Ar, C *meta*-CF), 132.6 (d, J = 9.7 Hz, 1C, Ar, C *meta*-CF), 124.3 (d, J = 2.9 Hz, 1C, Ar), 121.2 (s, 1C, Ar), 114.5 (d, J = 23.2 Hz, 1C, Ar, C *ortho*-CF), 80.5 (s, 1C, Boc), 44.8 (s, 1C, SO_2CH_3), 43.4 (s, 2C, NCH_2), 43.3 (s, 2C, NCH_2), 28.5 (s, 3C, Boc)

29. ^{1}H and ^{13}C-NMR spectrum of (1*R*,5*S*)-*tert*-butyl 8-(4-((2-chloro-4-(trifluoromethyl)phenyl)amino)-1,3,5-triazin-2-yl)-3,8-diazabicyclo[3.2.1]octane-3-carboxylate (**4i**)

^{1}H-NMR

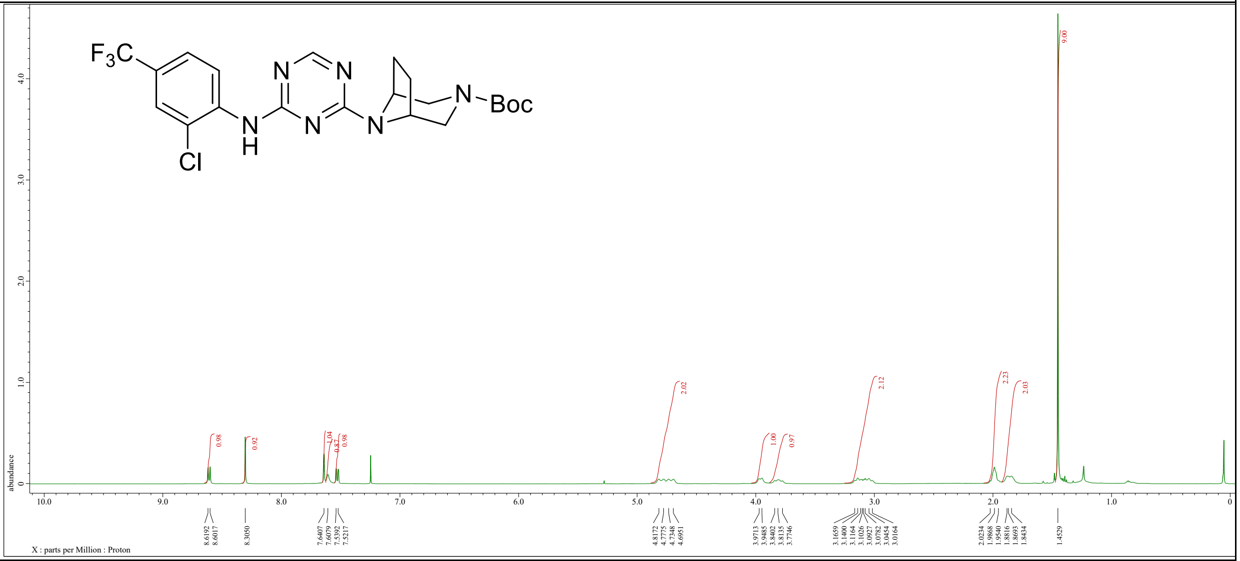

^{1}H-NMR (500 MHz, $CDCl_3$) δ 8.61 (d, J = 8.8 Hz, 1H, ArH), 8.30 (s, 1H, 1,3,5-triazine-H), 7.64 (s, 1H, ArH), 7.61 (s, 1H, NH), 7.53 (d, J = 8.8 Hz, 1H, ArH), 4.82 – 4.70 (m, 2H, NCH), 3.96 (d, J = 11.4 Hz, 1H, NCH_2), 3.84 – 3.77 (m, 1H, NCH_2), 3.17 – 3.02 (m, 2H, NCH_2), 2.02 – 1.95 (m, 2H, CCH_2), 1.88 – 1.84 (m, 2H, CCH_2), 1.45 (s, 9H, Boc)

^{13}C-NMR

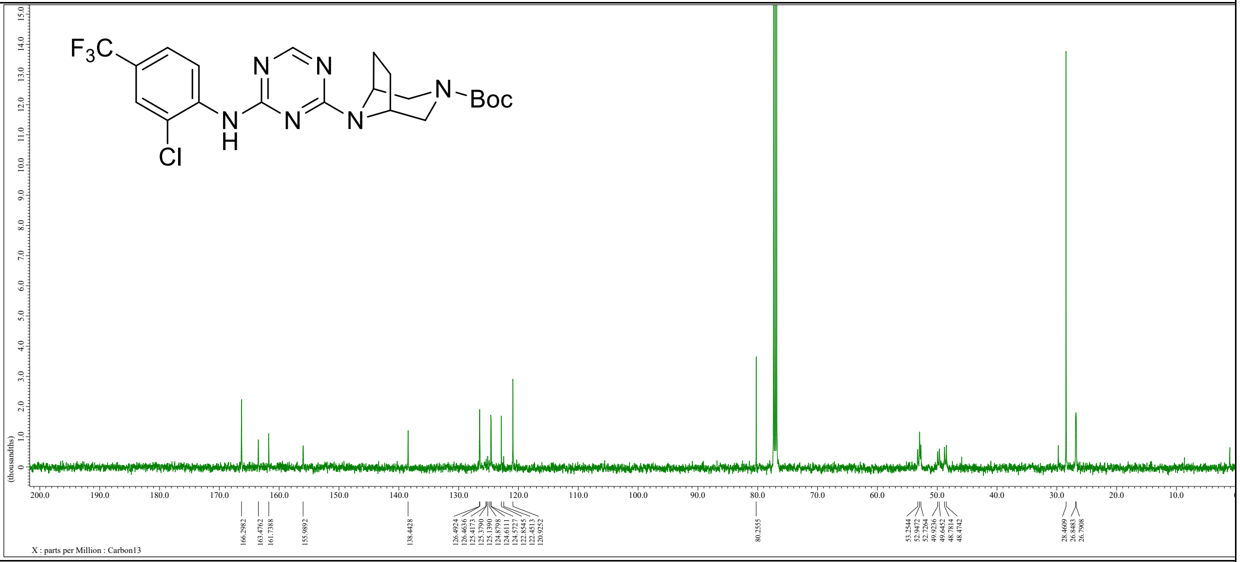

^{13}C-NMR (126 MHz, $CDCl_3$) (mixture of rotamers) δ 166.3 (s, 1C, 1,3,5-triazine), 163.5 (s, 1C, 1,3,5-triazine), 161.7 (s, 1C, 1,3,5-triazine), 156.0 (s, 1C, Boc), 138.4 (s, 1C, Ar), 126.5 (d, J = 3.6 Hz, Ar), 125.4 – 124.9 (m, 1C, Ar, C-CF_3), 124.6 (s, 1C, Ar), 123.5 (d, J = 272.2 Hz, 1C, CF_3), 122.9 (s, 1C, Ar), 120.9 (s, 1C, Ar), 80.3 (s, 1C, Boc), 53.3 – 52.7 (m, 2C, NCH), 49.9 – 48.5 (m, 2C, NCH_2), 28.5 (s, 3C, Boc), 26.8 – 26.8 (m, 2C, CCH_2)

30. ^{1}H and ^{13}C-NMR spectrum of (1*R*,5*S*)-*tert*-butyl 8-(4-((4-(trifluoromethyl)phenyl)amino)-1,3,5-triazin-2-yl)-3,8-diazabicyclo[3.2.1]octane-3-carboxylate (**4j**)

^{1}H-NMR

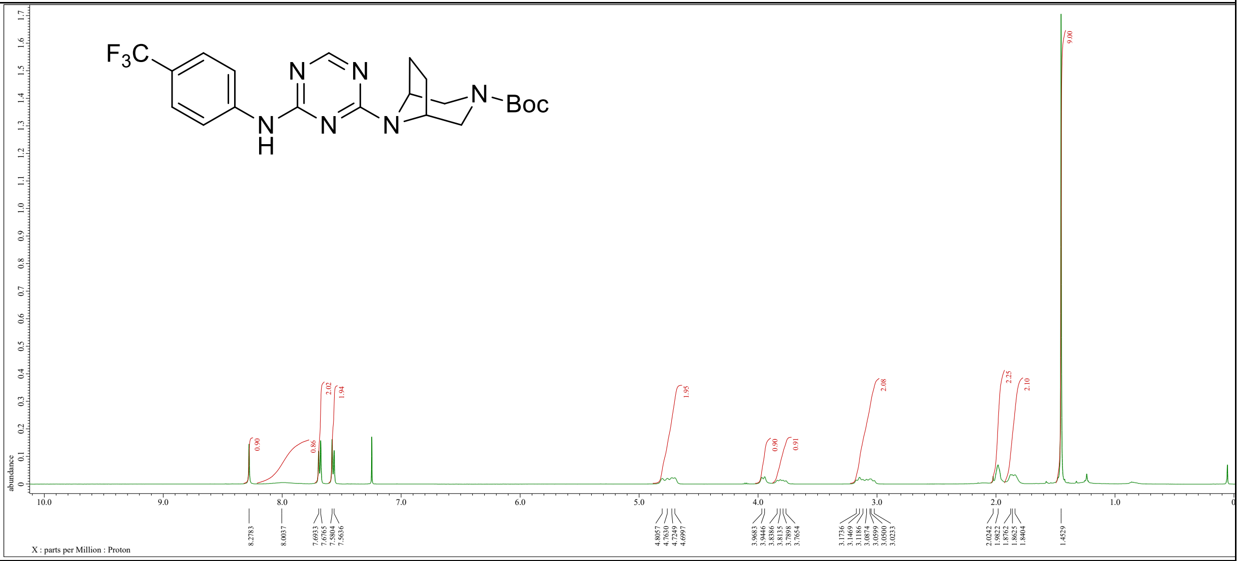

^{1}H-NMR (500 MHz, $CDCl_3$) δ 8.28 (s, 1H, 1,3,5-triazine-H), 8.00 (bs, 1H, NH), 7.68 (d, J = 8.4 Hz, 2H, ArH), 7.57 (d, J = 8.4 Hz, 2H, ArH), 4.81 – 4.70 (m, 2H, NCH), 3.96 (d, J = 11.8 Hz, 1H, NCH_2), 3.80 (q, J = 12.2 Hz, 1H, NCH_2), 3.17 – 3.02 (m, 2H, NCH_2), 2.02 – 1.98 (m, 2H, CCH_2), 1.88 – 1.84 (m, 2H, CCH_2), 1.45 (s, 9H, Boc)

^{13}C-NMR

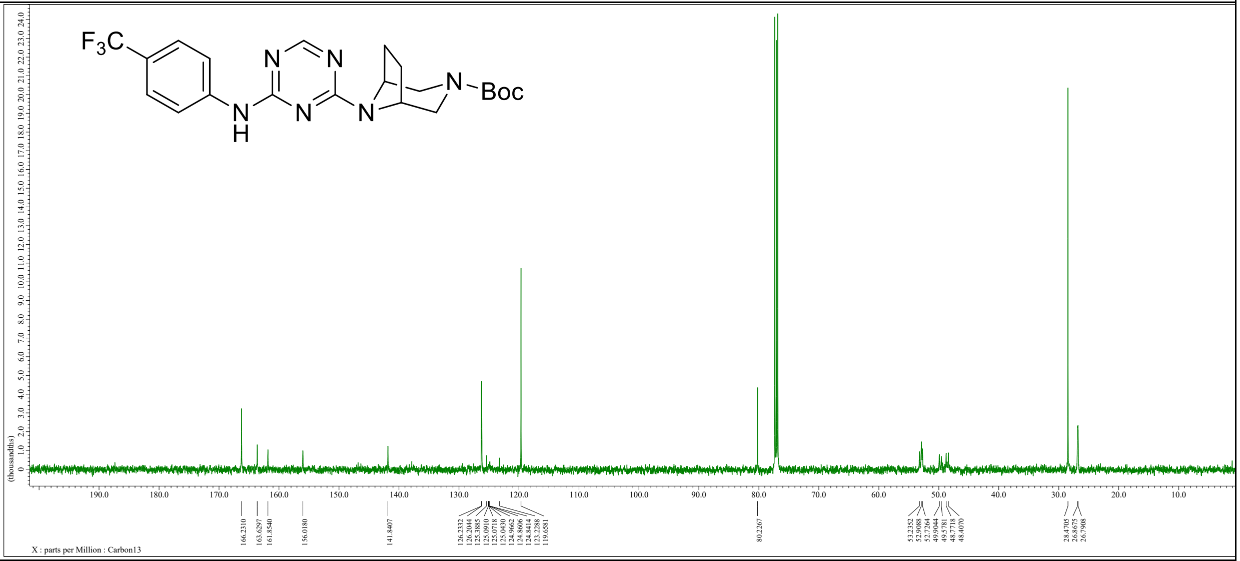

^{13}C-NMR (126 MHz, $CDCl_3$) (mixture of rotamers) δ 166.2 (s, 1C, 1,3,5-triazine), 163.6 (s, 1C, 1,3,5-triazine), 161.9 (s, 1C, 1,3,5-triazine), 156.0 (s, 1C, Boc), 141.8 (s, 1C, Ar), 126.2 (d, J = 2.4 Hz, 2C, Ar), 125.1 – 124.8 (m, 1C, Ar, C-CF_3), 124.3 (d, J = 272.2 Hz, 1C, CF_3), 119.7 (s, 2C, Ar), 80.2 (s, 1C, Boc), 53.2 – 52.7 (m, 2C, NCH), 49.9 – 48.4 (m, 2C, NCH_2), 28.5 (s, 3C, Boc), 26.9 – 26.8 (m, 2C, CCH_2)

31. ^{1}H and ^{13}C-NMR spectrum of (1*R*,5*S*)-*tert*-butyl 3-(4-((4-(trifluoromethyl)phenyl)amino)-1,3,5-triazin-2-yl)-3,8-diazabicyclo [3.2.1]octane-8-carboxylate (**4k**)

^{1}H-NMR

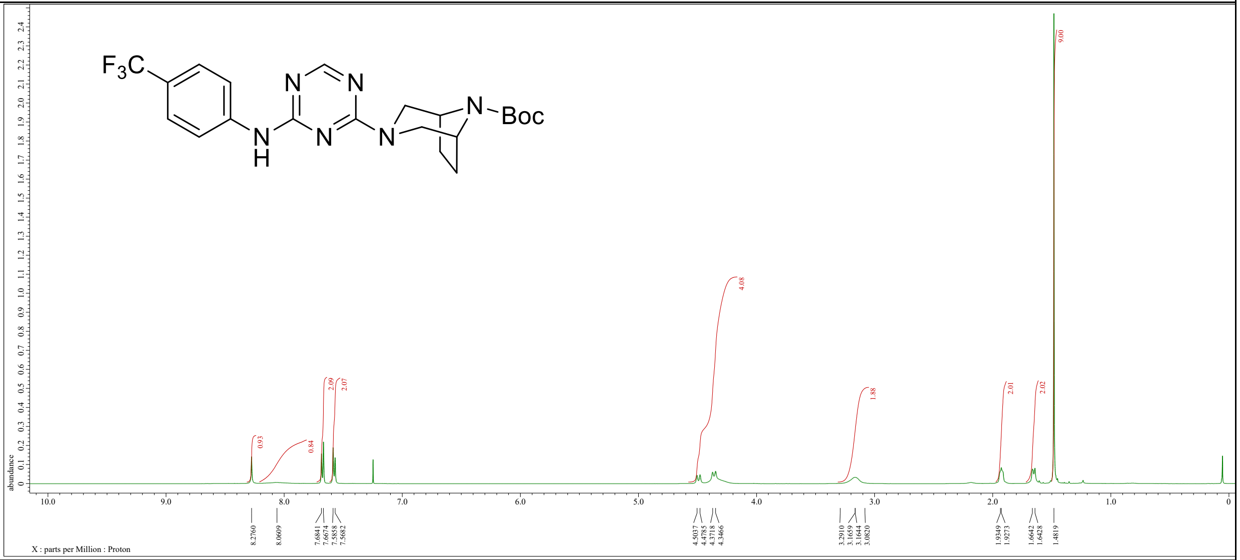

^{1}H-NMR (500 MHz, $CDCl_3$) δ 8.28 (s, 1H, 1,3,5-triazine-H), 8.06 (bs, 1H, NH), 7.68 (d, J = 8.4 Hz, 2H, ArH), 7.58 (d, J = 8.8 Hz, 2H, ArH), 4.50 – 4.35 (m, 4H, NCH, NCH_2), 3.29 – 3.08 (m, 2H, NCH_2), 1.93 (d, J = 3.8 Hz, 2H, CCH_2), 1.65 (d, J = 10.7 Hz, 2H, CCH_2), 1.48 (s, 9H, Boc)

^{13}C-NMR

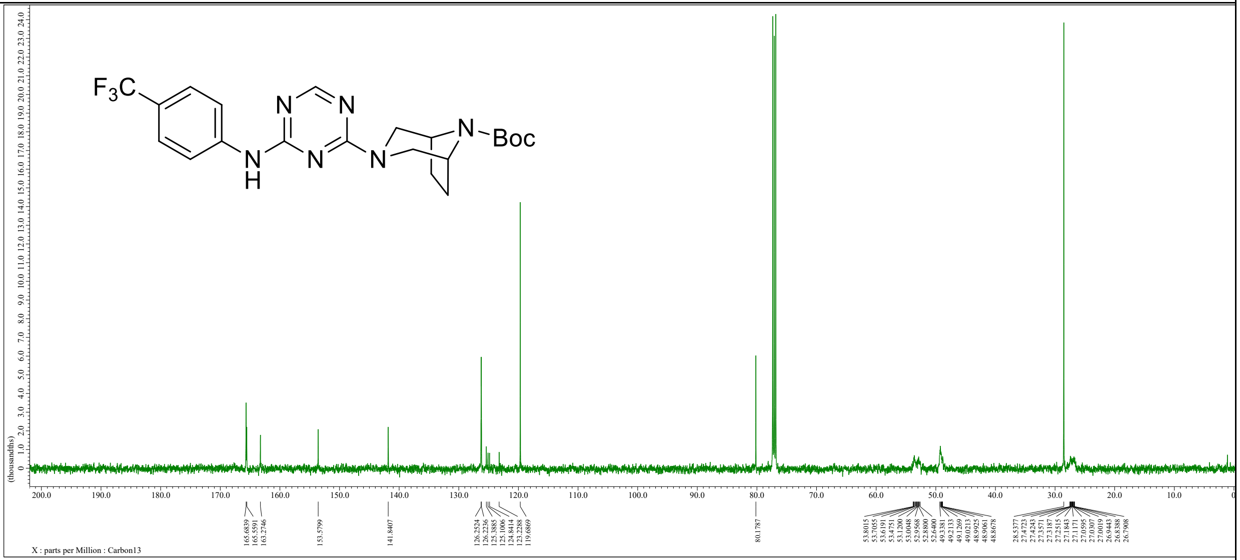

^{13}C-NMR (126 MHz, $CDCl_3$) (mixture of rotamers) δ 165.7 (s, 1C, 1,3,5-triazine), 165.6 (s, 1C, 1,3,5-triazine), 163.3 (s, 1C, 1,3,5-triazine), 153.6 (s, 1C, Boc), 141.8 (s, 1C, Ar), 126.3 (d, J = 3.5 Hz, 2C, Ar, C *ortho*-C-CF_3), 125.0 (d, J = 32.3 Hz, 1C, Ar, C-CF_3), 124.3 (d, J = 272.2 Hz, 1C, CF_3), 119.7 (s, 2C, Ar), 80.2 (s, 1C, Boc), 53.8 – 52.6 (m, 2C, NCH), 49.3 – 48.9 (m, 2C, NCH_2), 28.5 (s, 3C, Boc), 27.5 – 26.7 (m, 1C, CCH_2)

32. ^{1}H and ^{13}C-NMR spectrum of *tert*-butyl 4-((4-((4-(trifluoromethyl)phenyl)amino)-1,3,5-triazin-2-yl)amino)piperidine-1-carboxylate (**4l**)

^{1}H-NMR

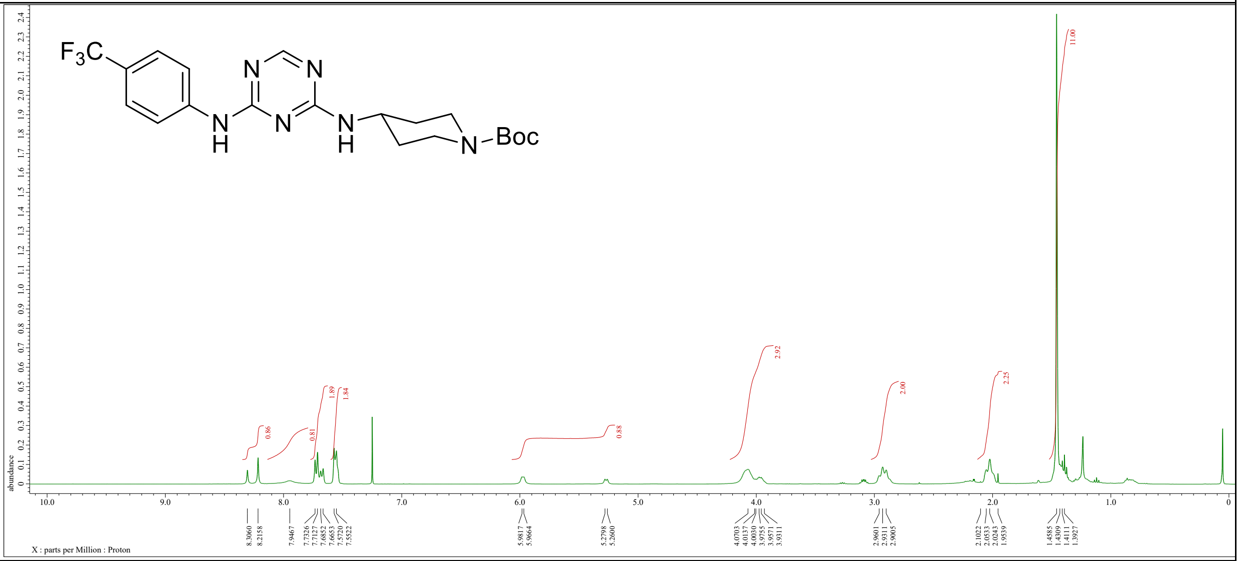

^{1}H-NMR (400 MHz, $CDCl_3$) (mixture of rotamers) δ 8.31 – 8.22 (m, 1H, 1,3,5-triazine-H), 7.95 (bs, 1H, NH), 7.73 – 7.67 (m, 2H, ArH), 7.56 (d, J = 7.9 Hz, 2H, ArH), 5.98 – 5.26 (m, 1H, NH piperidine), 4.07 – 3.93 (m, 3H, NCH, NCH_2), 2.93 (t, J = 11.9 Hz, 2H, NCH_2), 2.10 – 1.95 (m, 2H, CCH_2), 1.46 – 1.39 (m, 11H, CCH_2, Boc)

^{13}C-NMR

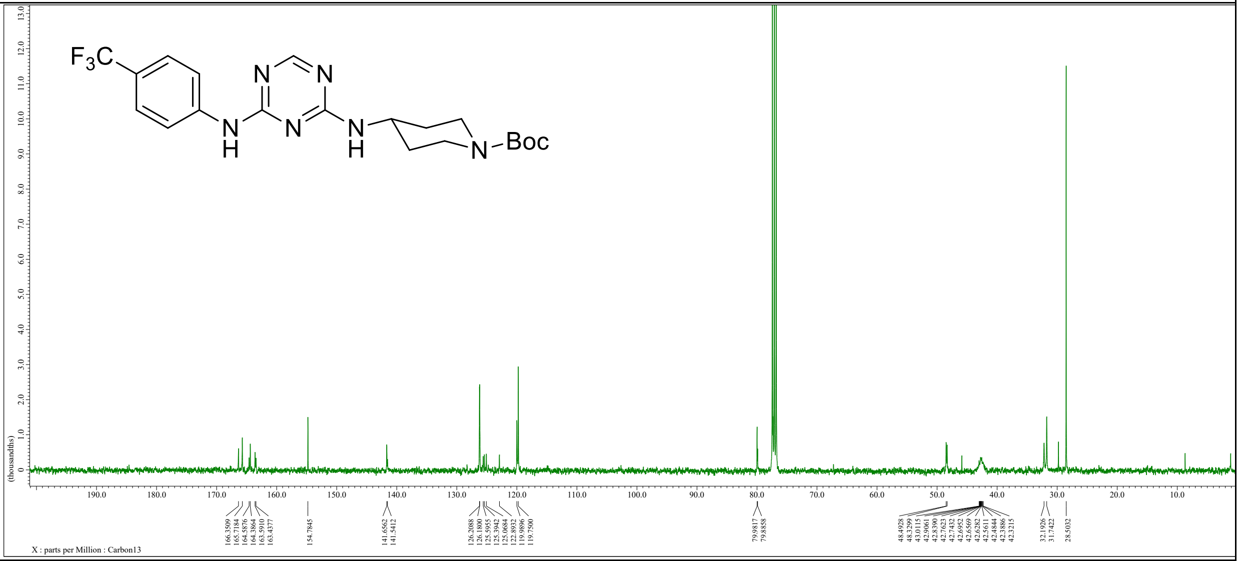

^{13}C-NMR (101 MHz, $CDCl_3$) (mixture of rotamers) δ 166.4 – 165.7 (m, 1C, 1,3,5-triazine), 164.6 – 164.4 (m, 1C, 1,3,5-triazine), 163.6 – 163.4 (s, 1C, 1,3,5-triazine), 154.8 (s, 1C, Boc), 141.7 – 141.5 (m, 1C, Ar), 126.2 (d, J = 2.9 Hz, 2C, Ar, C ortho-C-CF_3), 125.2 (d, J = 32.9 Hz, 1C, Ar, C-CF_3), 124.2 (d, J = 272.9 Hz, 1C, CF_3), 120.0 – 119.8 (m, 2C, Ar), 80.0 – 79.9 (m, 1C, Boc), 48.5 – 48.3 (m, 2C, NCH_2), 43.0 – 42.3 (m, 1C, NCH), 32.2 – 31.7 (m, 2C, CCH_2), 28.5 (s, 3C, Boc)

33. ^{1}H and ^{13}C-NMR spectrum of *tert*-butyl 4-(4-((4-(trifluoromethyl)phenyl)amino)-1,3,5-triazin-2-yl)piperazine-1-carboxylate (**4m**)

^{1}H-NMR

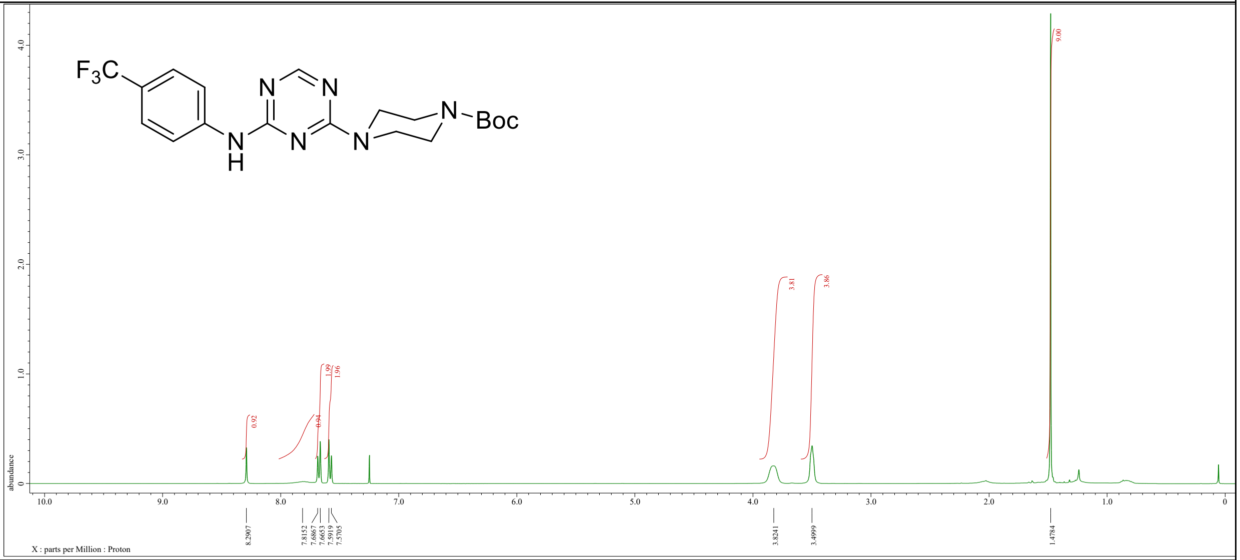

^{1}H-NMR (400 MHz, $CDCl_3$) δ 8.29 (s, 1H, 1,3,5-triazine-H), 7.82 (bs, 1H, NH), 7.68 (d, J = 8.6 Hz, 2H, ArH), 7.58 (d, J = 8.6 Hz, 2H, ArH), 3.82 (s, 4H, NCH_2), 3.50 (s, 4H, NCH_2), 1.48 (s, 9H, Boc)

^{13}C-NMR

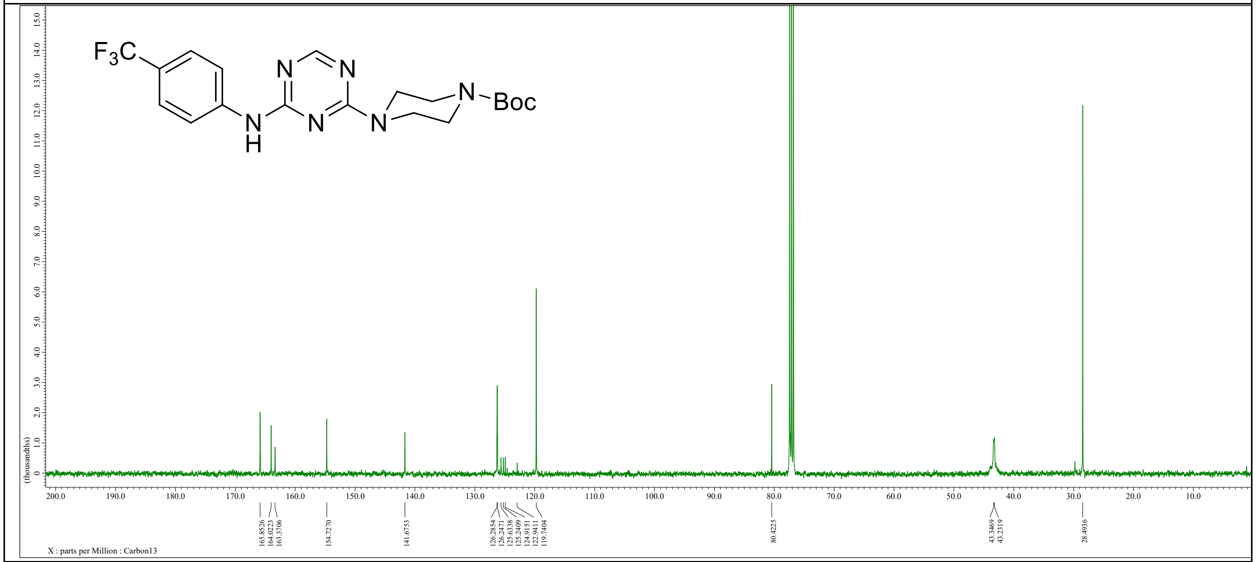

^{13}C-NMR (101 MHz, $CDCl_3$) δ 165.9 (s, 1C, 1,3,5-triazine), 164.0 (s, 1C, 1,3,5-triazine), 163.4 (s, 1C, 1,3,5-triazine), 154.7 (s, 1C, Boc), 141.7 (s, 1C, Ar), 126.3 (d, J = 3.8 Hz, 2C, Ar, C *ortho*-C-CF_3), 125.1 (d, J = 32.9 Hz, 1C, Ar, C-CF_3), 124.3 (d, J = 274.7 Hz, 1C, CF_3), 119.7 (s, 2C, Ar), 80.4 (s, 1C, Boc), 43.3 (s, 2C, NCH_2), 43.2 (s, 2C, NCH_2), 28.5 (s, 3C, Boc)

34. ^{1}H and ^{13}C-NMR spectrum of (1*R,*5*S*)-*tert*-butyl 8-(4-((2-fluoro-4-(trifluoromethyl)phenyl)amino)-1,3,5-triazin-2-yl)-3,8-diazabicyclo[3.2.1]octane-3-carboxylate (**4n**)

^{1}H-NMR

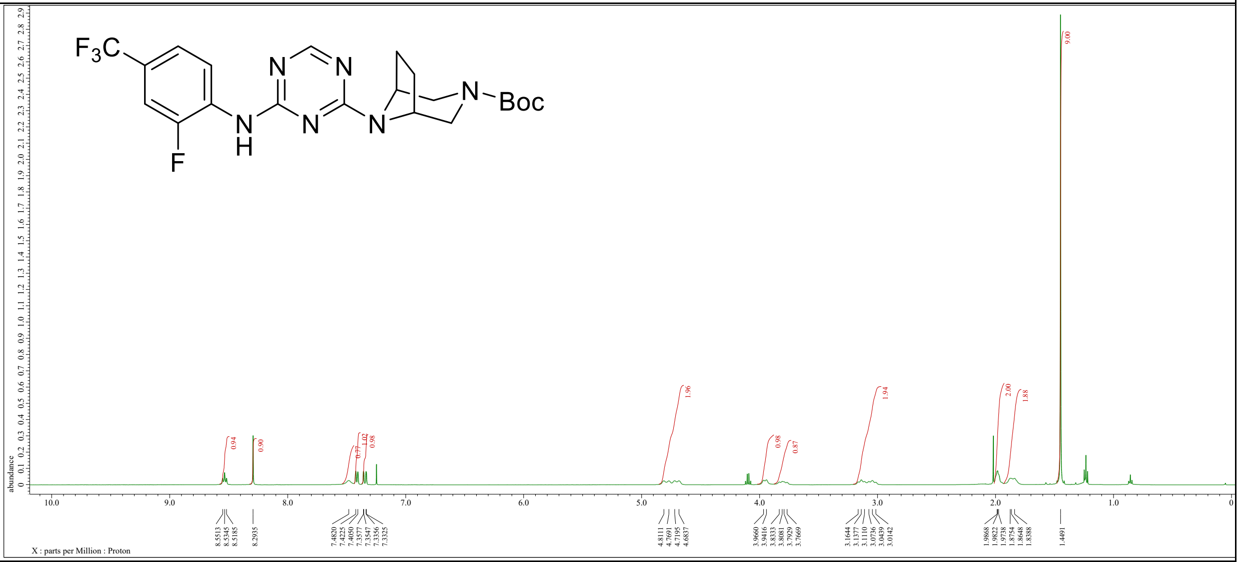

^{1}H-NMR (500 MHz, $CDCl_3$) δ 8.53 (t, J = 8.2 Hz, 1H, ArH), 8.29 (s, 1H, 1,3,5-triazine-H), 7.48 (bs, 1H, NH), 7.41 (d, J = 8.8 Hz, 1H, ArH), 7.35 (dd, J = 11.1, 1.5 Hz, 1H, ArH, H *ortho*-CF), 4.81 – 4.68 (m, 2H, NCH), 3.95 (d, J = 12.2 Hz, 1H, NCH_2), 3.83 – 3.77 (m, 1H, NCH_2), 3.16 – 3.01 (m, 2H, NCH_2), 1.99 – 1.97 (m, 2H, CCH_2), 1.88 – 1.84 (m, 2H, CCH_2), 1.45 (s, 9H, Boc)

^{13}C-NMR

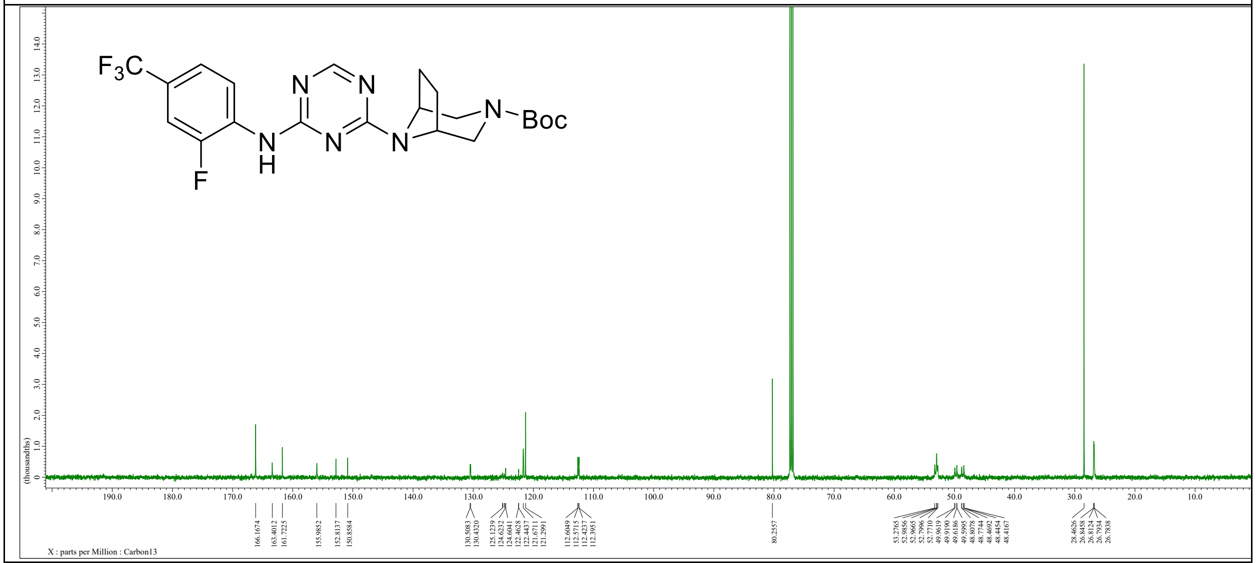

^{13}C-NMR (126 MHz, $CDCl_3$) (mixture of rotamers) δ 166.2 (s, 1C, 1,3,5-triazine), 163.4 (s, 1C, 1,3,5-triazine), 161.7 (s, 1C, 1,3,5-triazine), 156.0 (s, 1C, Boc), 151.8 (d, J = 246.5 Hz, Ar, C-F), 130.5 (d, J = 9.6 Hz, 1C, Ar, C *meta*-CF), 125.1 – 124.8 (m, 1C, Ar), 123.5 (dd, J = 272.2, 2.4 Hz, 1C, CF_3), 121.7 (s, 1C, Ar), 121.3 (s, 1C, Ar), 112.50 (dd, J = 22.8, 4.2 Hz, 1C, Ar, C *ortho*-CF), 80.3 (s, 1C, Boc), 53.3 – 52.8 (m, 2C, NCH_2), 50.0 – 48.4 (m, 2C, NCH), 28.5 (s, 3C, Boc), 26.8 – 26.8 (m, 2C, CCH_2)

35. ^{1}H and ^{13}C-NMR spectrum of (1*R*,5*S*)-*tert*-butyl 3-(4-((2-fluoro-4-(trifluoromethyl)phenyl) amino)-1,3,5-triazin-2-yl)-3,8-diazabicyclo[3.2.1]octane-8-carboxylate (**4o**)

^{1}H-NMR

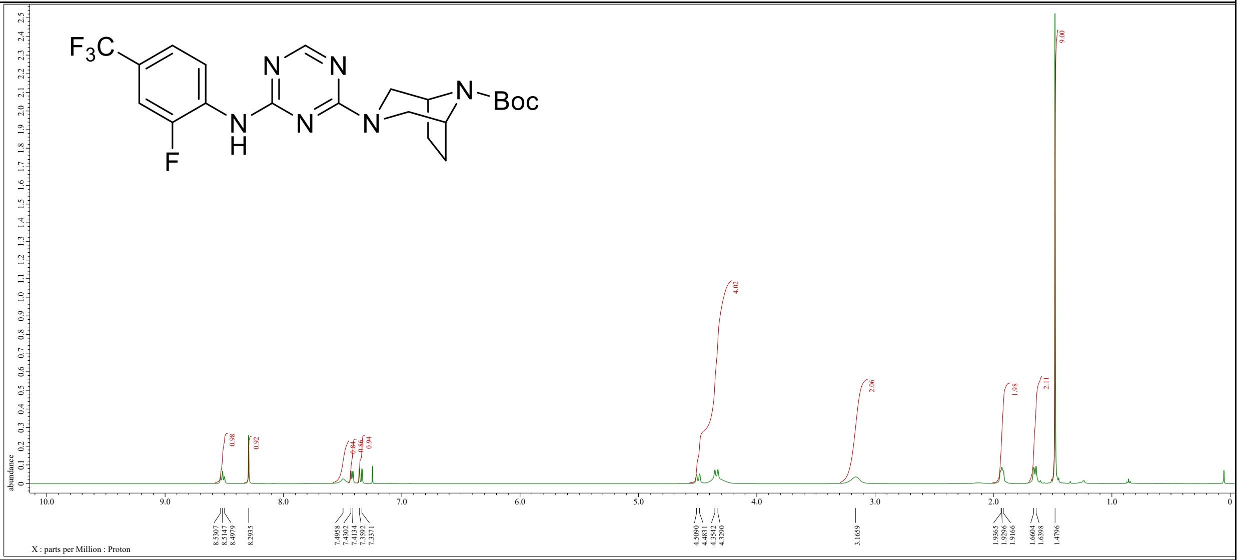

^{1}H-NMR (500 MHz, $CDCl_3$) δ 8.51 (t, J = 8.2 Hz, 1H, ArH), 8.29 (s, 1H, 1,3,5-triazine), 7.50 (bs, 1H, NH), 7.42 (d, J = 8.4 Hz, 1H, ArH), 7.35 (d, J = 11.1 Hz, 1H, ArH, H *ortho*-CF), 4.51 – 4.33 (m, 4H, NCH, NCH_2), 3.17 (s, 2H, NCH_2), 1.94 – 1.92 (m, 2H, CCH_2), 1.65 (d, J = 10.3 Hz, 2H, CCH_2), 1.48 (s, 9H, Boc)

^{13}C-NMR

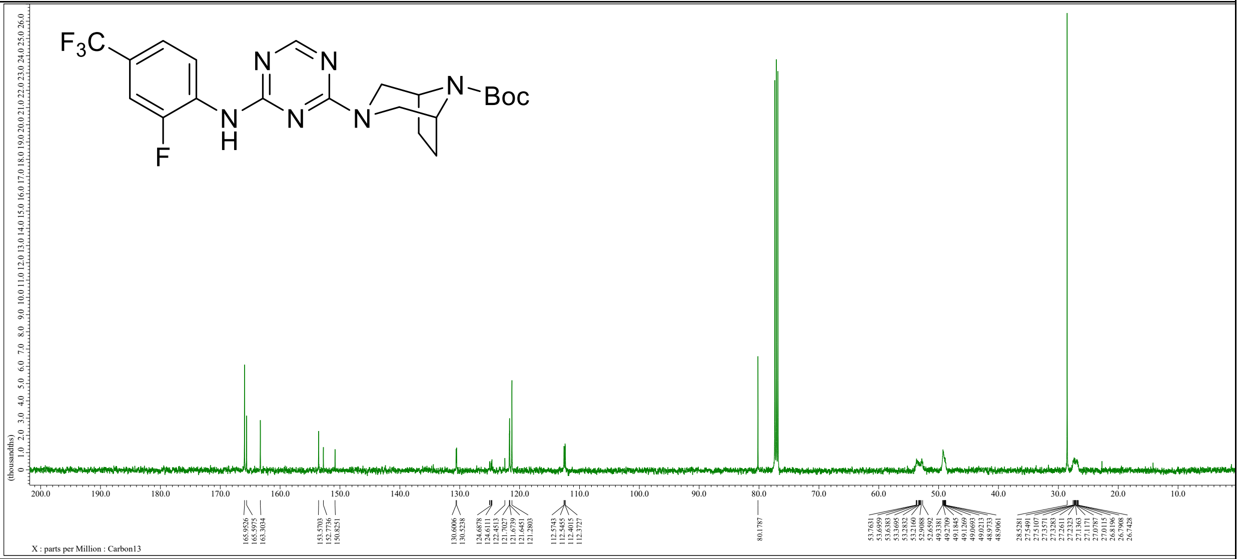

^{13}C-NMR (126 MHz, $CDCl_3$) (mixture of rotamers) δ 166.0 (s, 1C, 1,3,5-triazine), 165.6 (s, 1C, 1,3,5-triazine), 163.3 (s, 1C, 1,3,5-triazine), 153.6 (s, 1C, Boc), 151.8 (d, J = 245.6 Hz, 1C, Ar, C-F), 130.6 (d, J = 9.7 Hz, 1C, Ar, C *meta*-CF), 124.9 (dd, J = 33.9, 7.2 Hz, 1C, Ar, C-CF_3 *meta*-CF), 123.5 (d, J = 272.2 Hz, 1C, CF_3), 121.7 (t, J = 3.7 Hz, Ar), 121.3 (s, 1C, Ar), 112.5 (dd, J = 21.7, 3.7 Hz, Ar, C *ortho*-CF), 80.2 (s, 1C, Boc), 53.8 – 52.7 (m, 2C, NCH), 49.3 – 48.8 (m, 2C, NCH_2), 28.5 (s, 3C, Boc), 27.5 – 26.6 (m, 2C, CCH_2)

36. ^{1}H and ^{13}C-NMR spectrum of *tert*-butyl 4-((4-((2-fluoro-4-(trifluoromethyl)phenyl)amino)-1,3,5-triazin-2-yl)amino)piperidine-1-carboxylate (**4p**)

^{1}H-NMR

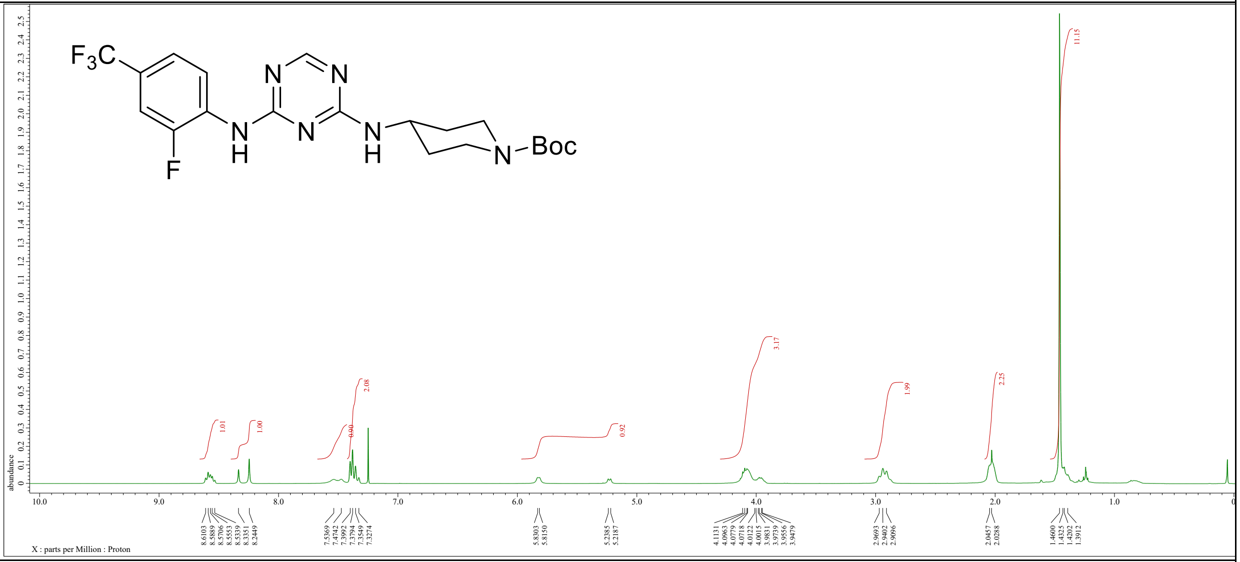

^{1}H-NMR (400 MHz, CDCl$_3$) (mixture of rotamers) δ 8.61 – 8.53 (m, 1H, ArH), 8.34 – 8.24 (m, 1H, 1,3,5-triazine-H), 7.54 – 7.47 (m, 1H, NH), 7.40 – 7.33 (m, 2H, ArH), 5.83 – 5.22 (m, 1H, NH piperidine), 4.11 – 3.95 (m, 3H, NCH), 2.97 – 2.91 (m, 2H, NCH$_2$), 2.05 – 2.03 (m, 2H, CCH$_2$), 1.46 – 1.39 (m, 11H, CCH$_2$, Boc)

^{13}C-NMR

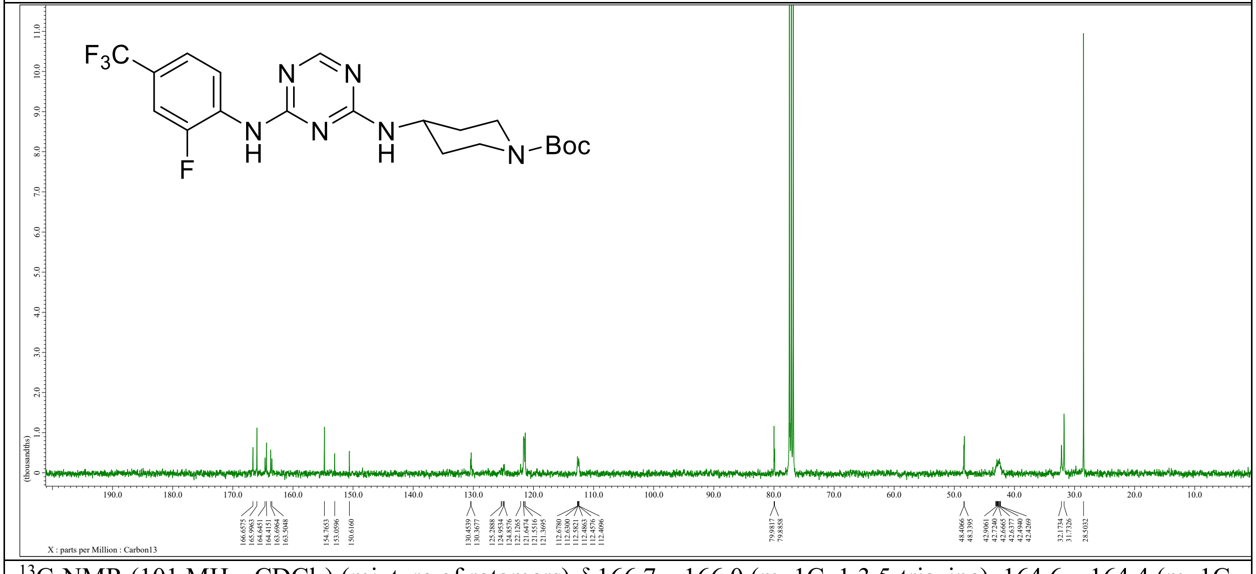

^{13}C-NMR (101 MHz, CDCl$_3$) (mixture of rotamers) δ 166.7 – 166.0 (m, 1C, 1,3,5-triazine), 164.6 – 164.4 (m, 1C, 1,3,5-triazine), 163.7 – 163.5 (m, 1C, 1,3,5-triazine), 154.8 (s, 1C, Boc), 151.8 (d, J = 246.8 Hz, 1C, Ar, C-F), 130.4 (d, J = 8.7 Hz, 1C, Ar, C *meta*-CF), 125.2 (dd, J = 33.9, 7.8 Hz, 1C, Ar, C-CF$_3$ *meta*-CF), 123.5 (d, J = 275.8 Hz, 1C, CF$_3$), 121.6 (d, J = 9.6 Hz, 1C, Ar), 121.4 (s, 1C, Ar), 112.7 – 112.4 (m, 1C, Ar), 80.0 – 79.9 (m, 1C, Boc), 48.4 – 48.3 (m, 2C, NCH$_2$), 43.1 – 42.3 (m, 1C, NCH), 32.2 – 31.7 (m, 2C, CCH$_2$), 28.5 (s, 3C, Boc)

37. ^{1}H and ^{13}C-NMR spectrum of *tert*-butyl 4-(4-((2-fluoro-4-(trifluoromethyl)phenyl)amino)-1,3,5-triazin-2-yl)piperazine-1-carboxylate (**4q**)

^{1}H-NMR

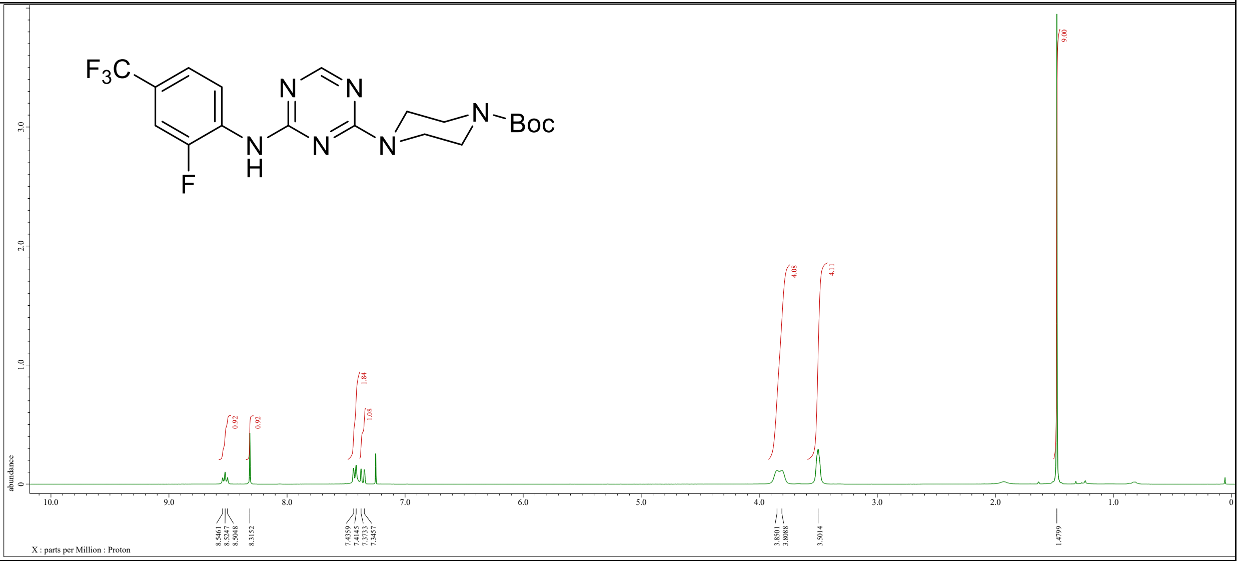

^{1}H-NMR (400 MHz, $CDCl_3$) δ 8.53 (t, J = 8.3 Hz, 1H, ArH), 8.32 (s, 1H, 1,3,5-triazine-H), 7.43 (d, J = 8.6 Hz, 2H, ArH, NH), 7.36 (d, J = 11.0 Hz, 1H, ArH, H *ortho*-CF), 3.85 – 3.81 (m, 4H, NCH_2), 3.50 (s, 4H, NCH_2), 1.48 (s, 9H, Boc)

^{13}C-NMR

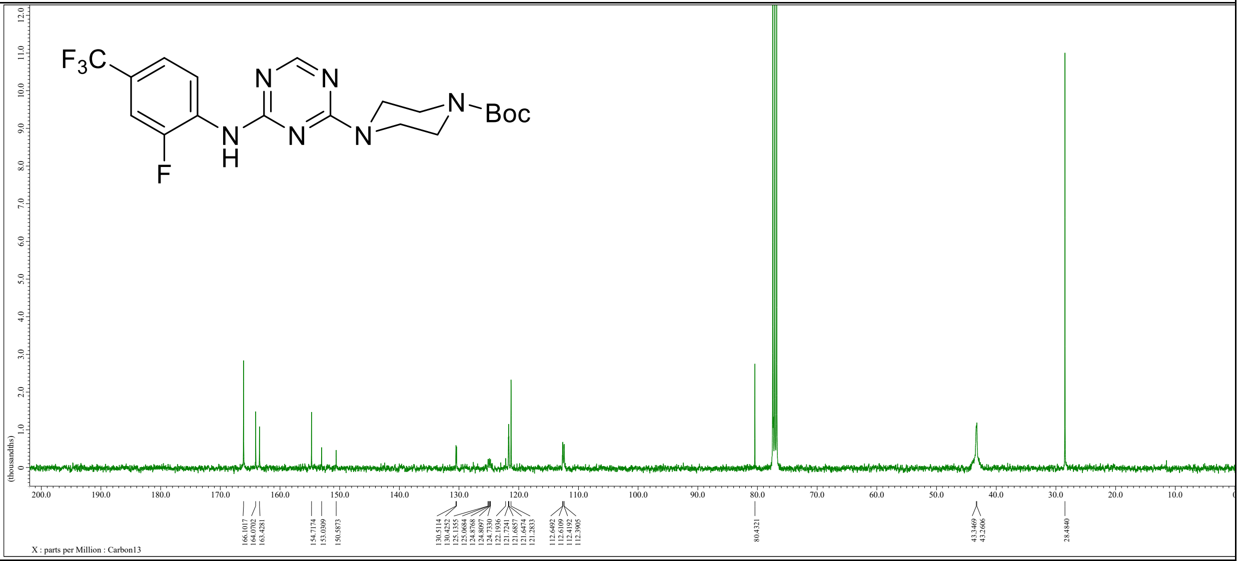

^{13}C-NMR (101 MHz, $CDCl_3$) δ 166.1 (s, 1C, 1,3,5-triazine), 164.1 (s, 1C, 1,3,5-triazine), 163.4 (s, 1C, 1,3,5-triazine), 154.7 (s, 1C, Boc), 151.8 (d, J = 246.8 Hz, Ar, C-F), 130.5 (d, J = 8.7 Hz, 1C, Ar, C *meta*-CF), 125.0 (dd, J = 26.2, 6.7 Hz, 1C, Ar, C-CF_3 *meta*-CF), 123.5 (d, J = 256.4 Hz, 1C, CF_3), 121.7 (t, J = 3.9 Hz, Ar), 121.3 (s, 1C, Ar), 112.5 (dd, J = 23.2, 3.8 Hz, 1C, Ar, C *ortho*-CF), 80.4 (s, 1C, Boc), 43.3 (s, 2C, NCH_2), 43.3 (s, 2C, NCH_2), 28.5 (s, 3C, Boc)

II. HRMS spectra of compounds 3c, 3d, and 3g

1. HRMS spectra of compound **3c**

[Mass Spectrum]
Data : Compound 5_HR 1 Date : 24-Aug-2026 15:05
RT : 0.77 min Scan# : 21
Elements : C 20/0, H 41/0, 35Cl 1/0, 37Cl 1/0, F 1/0, N 7/0, O 2/0
Mass Tolerance : 1000ppm, 5mmu if m/z < 5, 10mmu if m/z > 10
Unsaturation (U.S.) : 10.0 - 20.0

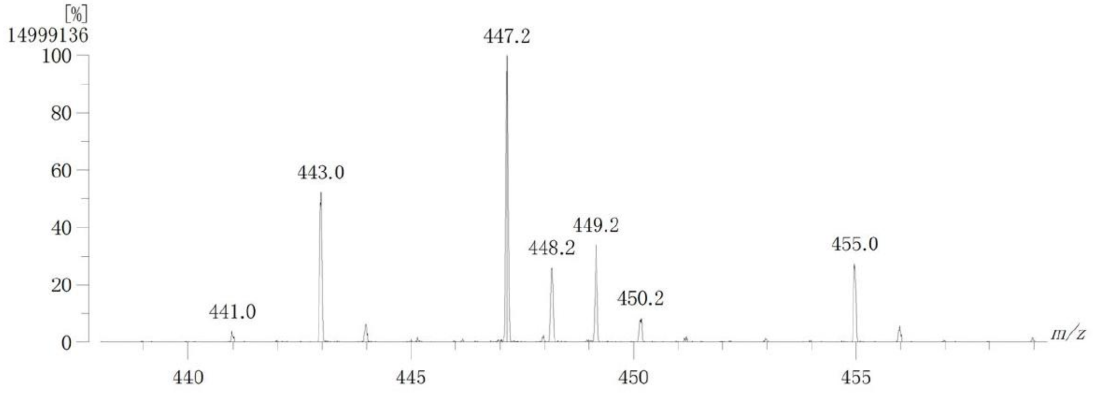

	Observed m/z	Int%	Err[ppm / mmu]	U.S.	Composition
1	447.1585	100.00	-0.2 / -0.1	12.0	C20 H23 35Cl F N7 O2

2. HRMS spectra of compound **3d**

[Mass Spectrum]
Data : Compound 7_HR 2 Date : 24-Aug-2026 14:58
RT : 0.77 min Scan# : 21
Elements : C 19/0, H 39/0, 35Cl 1/0, 37Cl 1/0, F 1/0, N 7/0, O 2/0
Mass Tolerance : 1000ppm, 5mmu if m/z < 5, 10mmu if m/z > 10
Unsaturation (U.S.) : 10.0 - 20.0

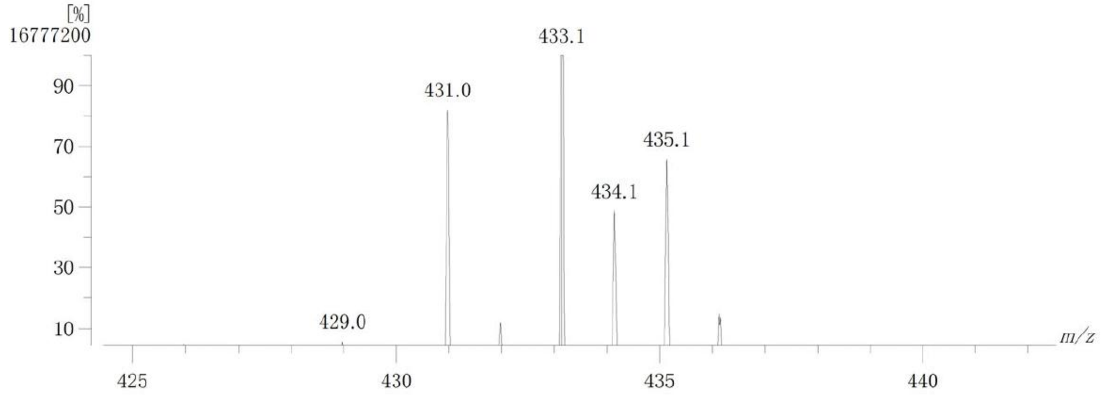

	Observed m/z	Int%	Err[ppm / mmu]	U.S.	Composition
1	433.1430	100.00	+0.2 / +0.1	12.0	C19 H21 35Cl F N7 O2

3. HRMS spectra of compound **3g**

Spectrum

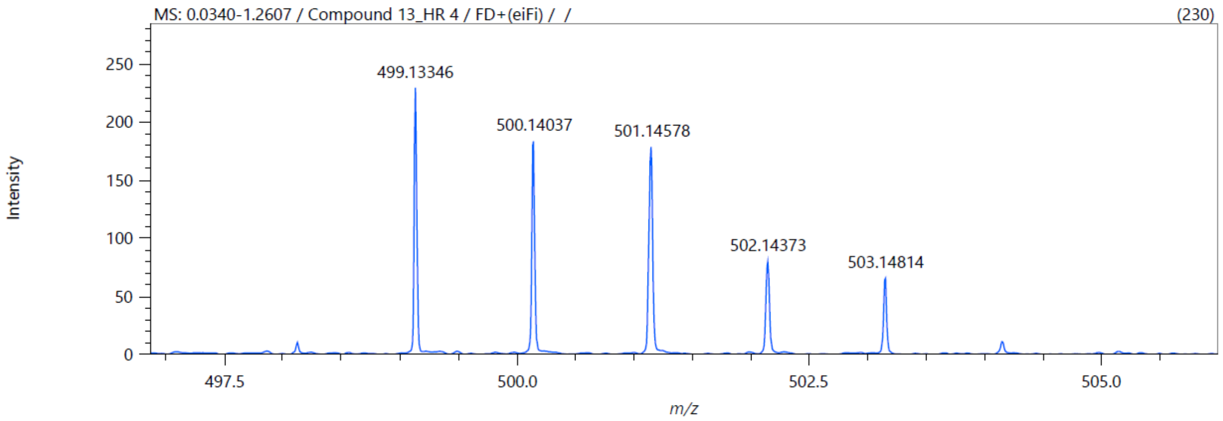

Elemental Composition

Parameters

Tolerance:	±20.00 mDa
Electron:	Odd/Even
Charge:	+1
DBE:	-1.5 - 20.0

Elements Set 1:

Symbol	C	H	O	Cl	F	N	S
Min	0	0	0	0	0	0	0
Max	20	41	4	1	1	6	1

Results

Mass	Intensity	Formula	Calculated Mass	Mass Difference [mDa]	Mass Difference [ppm]	DBE
500.14037	183.57	C20 H26 N6 O4 F S Cl	500.14033	0.04	0.08	10.0

III. Cytotoxicity validation of compounds 3d and 3g

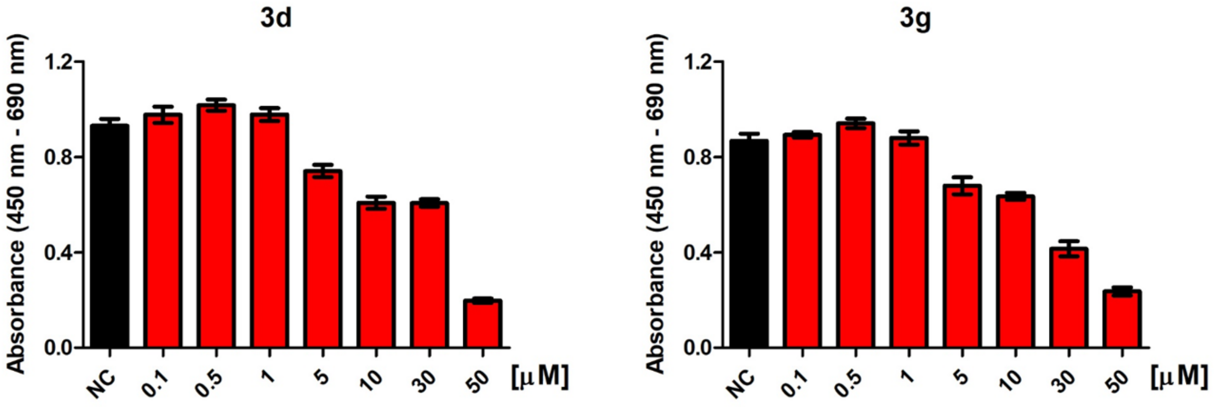

Figure S1. Cytotoxicity of compounds 3d and 3g in HEK293T cells.

HEK293T cells were seeded at a density of 3×10^4 cells per well in Poly-D-lysine-precoated 96-well plates and cultured in DMEM supplemented with 10% fetal bovine serum (FBS) and 1% penicillin/streptomycin (P/S). On the following day, compounds **3d** and **3g** were treated in a concentration-dependent manner over a range of 0.1–50 μM in fresh DMEM containing 10% FBS and 1% P/S. After 24 h of treatment, cell viability was assessed using WST-1 reagent (Roche, Mannheim, German). The absorbance was measured at 450 nm and then at 690 nm, which was used as the background reference wavelength, using a VICTOR3 spectrophotometer (PerkinElmer Inc., Foster City, CA, USA). All experiments were independently repeated at least three times. Compounds **3d** and **3g** showed a reduction in cell viability starting at approximately 5 μM, with pronounced cytotoxic effects observed at concentrations of 10 μM and above.
